# Chemical Synthesis of *Burkholderia* Lipid A Modified with Glycosyl Phosphodiester-Linked 4-Amino-4-deoxy-β-l-arabinose and Its Immunomodulatory Potential

**DOI:** 10.1002/chem.201406058

**Published:** 2015-01-28

**Authors:** Ralph Hollaus, Simon Ittig, Andreas Hofinger, Mira Haegman, Rudi Beyaert, Paul Kosma, Alla Zamyatina

**Affiliations:** [a] Department of Chemistry, University of Natural Resources and Life Sciences Muthgasse 18, 1190 Vienna (Austria) E-mail: alla.zamyatina@boku.ac.at; [b] Biozentrum, University of Basel Klingelbergstrasse 50/70, 4056 Basel (Switzerland); [c] Department for Biomedical Molecular Biology, Unit of Molecular Signal Transduction in Inflammation, Ghent University, Inflammation Research Center VIB, Technologiepark 927, 9052 Ghent (Belgium)

**Keywords:** carbohydrates, glycolipids, glycosyl phosphates, lipopolysaccharide, structure–activity relationships

## Abstract

Modification of the Lipid A phosphates by positively charged appendages is a part of the survival strategy of numerous opportunistic Gram-negative bacteria. The phosphate groups of the cystic fibrosis adapted *Burkholderia* Lipid A are abundantly esterified by 4-amino-4-deoxy-β-l-arabinose (β-l-Ara4N), which imposes resistance to antibiotic treatment and contributes to bacterial virulence. To establish structural features accounting for the unique pro-inflammatory activity of *Burkholderia* LPS we have synthesised Lipid A substituted by β-l-Ara4N at the anomeric phosphate and its Ara4N-free counterpart. The double glycosyl phosphodiester was assembled by triazolyl-tris-(pyrrolidinyl)phosphonium-assisted coupling of the β-l-Ara4N H-phosphonate to α-lactol of β(1→6) diglucosamine, pentaacylated with (*R*)-(3)-acyloxyacyl- and Alloc-protected (*R*)-(3)-hydroxyacyl residues. The intermediate 1,1′-glycosyl-H-phosphonate diester was oxidised in anhydrous conditions to provide, after total deprotection, β-l-Ara4N-substituted *Burkholderia* Lipid A. The β-l-Ara4N modification significantly enhanced the pro-inflammatory innate immune signaling of otherwise non-endotoxic *Burkholderia* Lipid A.

## Introduction

The *B. cepacia* complex (BCC) is a group of opportunistic bacterial species that can cause severe lung infections and overwhelming septicaemia in patients with cystic fibrosis (CF) resulting in extremely high mortality (the “cepacia syndrome”).[1] Lipopolysaccharide (LPS) belongs to the major virulence factors of BCC species.[2] The pro-inflammatory activity of lipooligosaccharides (LOS) from BCC isolates has been extensively studied. Heterogeneous tetra- and pentaacylated LOS/Lipid A isolates from *B. mallei*,[3] *B. multivorans*,[4] *B. cenocepacia*,[5, 6] *B. cepacia*[2] and *B. dolosa*[7] were reported to be very potent activators of human (h) LPS-sensing innate immune receptor, Toll-like Receptor 4 (TLR4)–myeloid differentiation-2 (MD-2) complex. Furthermore, it has been previously established that the terminal membrane-bound portion of LPS, glycophospholipid Lipid A, is primarily responsible for induction of pro-inflammatory signalling, which was shown both with isolated[8] and synthetic Lipid A derivatives.[9, 10] The structural basis of the LPS-triggered activation of innate immune response was revealed in the seminal work deciphering the co-crystal structure of *E.coli Re*-LPS with human MD-2**⋅**TLR4 complex.[11]

**Figure 1:**
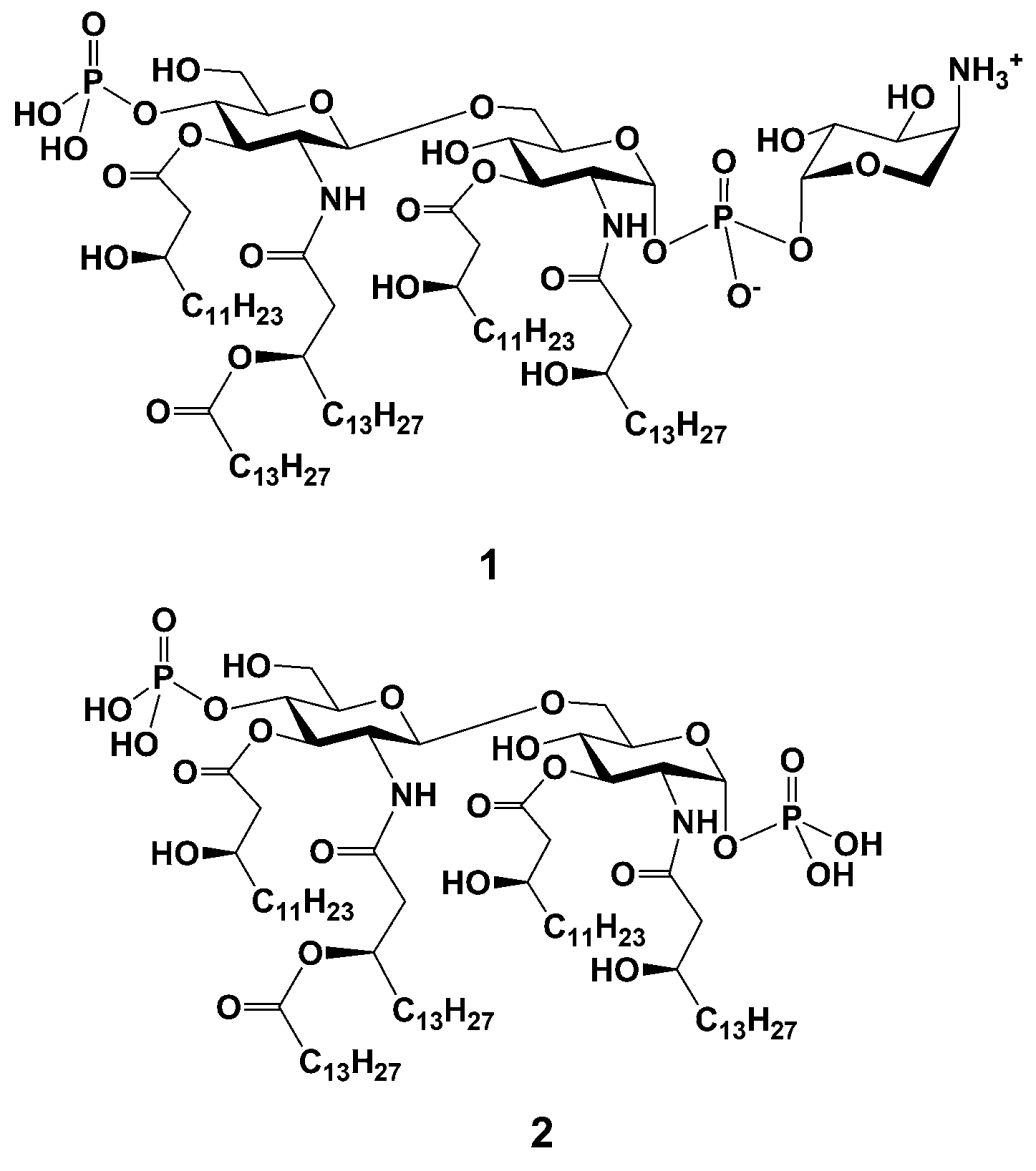
Major *Burkholderia* Lipid A 1 substituted by β-l-Ara4N at the anomerically linked phosphate and unmodified *Burkholderia* Lipid A 2.

Lipid A possesses a rather conserved structure that is characterized by a β(1→6)-linked diglucosamine backbone substituted by the long chain (*R*)-3-acyloxyacyl- and/or (*R*)-3-hydroxyacyl residues at positions 2, 3, 2′ and 3′ and the phosphate groups attached at positions 1 and 4′.[12] In some LPS the Lipid A phosphates are further substituted by compounds that reduce its net negative charge, such as ethanolamine,[13, 14] 4-amino-4-deoxy-β-l-arabinose (β-l-Ara4N)[2, 15] or galactosamine in *Francisella*[13] and glucosamine in *Bordetella* species.[16] These covalent modifications confer resistance to the endogenous cationic antimicrobial peptides (CAMPs) and aminoglycosides and are associated with increased bacterial virulence.[17, 18] Unlike *E. coli* Lipid A, which encounters a substitution by β-l-Ara4N only under particular laboratory stress conditions,[19] *Burkholderia* species express a very intricate β-l-Ara4N-modified Lipid A when isolated from the specimens of CF patients (Figure 1).[2] Progressive antibiotic treatment imposes selective pressure on BCC in the airways of immunocompromised patients, leading to substitution of the Lipid A phosphates with Ara4N, which results in reduction of ionic attraction and, as a consequence, in an amplified resistance to CAMPs and aminoglycosides.[1] Thus, covalent modification of the Lipid A phosphates with Ara4N, which is associated with chronic inflammation and decreased survival, is considered as a crucial attribute for the virulence of the CF adapted BCC species.[2]

Interestingly, β-l-Ara4N-1-phosphate residues were found to be characteristic also for pentaacylated Lipid A structures of an opportunistic pathogen *Serratia marcescens*[20] as well as of *B. thailandensis* and *B. pseudomallei*, a causative agent of melioidosis (responsible for chronic abscesses and acute septicaemia),[21] contributing to pathogenesis and endotoxicity. Similarly, *P. aeruginosa* Lipid A isolated from the airways of CF patients entails a unique Ara4N modification exclusively at the 1-phosphate, promoting acute inflammation of the airways.[22]

Although it is generally believed that only hexaacylated Lipid A patterns (such as from *E. coli*) are capable of eliciting a robust TLR4-mediated innate immune response,[23, 24] underacylated Ara4N-modified *Burkholderia* Lipid A/LOS isolates efficiently induce the innate immune signalling, which is comparable to that produced by hexaacylated *E. coli* LPS.[5] A high degree of heterogeneity of LOS/Lipid A obtained from bacterial isolates with regard to the lipid chain content and the degree of Ara4N substitution at the Lipid A phosphates, which is usually given like “non-stoichiometric”, makes it difficult to assess which structural features of *Burkholderia* Lipid A, such as acylation pattern, the length of (*R*)-3-hydroxyacyl chains (C_16_–C_14_)[25, 26] (compared with C_12_–C_14_ in *E. coli* LPS) or modification of the phosphates with Ara4N, are responsible for the atypical pro-inflammatory activity. Of particular interest are the Lipid A structures corresponding to highly pro-inflammatory *B. cenocepacia*[5] and *B. caryophilli*[27] LPS, which are esterified by Ara4N exclusively at the anomerically-linked 1-phosphate. Since the 1-phosphate of *E. coli* Lipid A is directly involved in the homodimerization of MD-2**⋅**TLR4-LPS complexes, which results in the downstream inflammatory signalling,[11] the appendage of Ara4N possessing a positively charged amino group could enhance ionic attraction at the dimerization interface, thereby tightening the ligand–protein binding.

To establish the structural aspects accounting for the unique immuno-stimulating potential of *Burkholderia* Lipid A and to clarify the functional outcome of the Ara4N modification, the chemical synthesis of homogeneous structurally defined Lipid A corresponding to native *Burkholderia* LPS is required. The availability of purely synthetic Ara4N-modified *Burkholderia* Lipid A would also provide a reliable tool for extensive biosynthetic studies. Herein, we report on the total synthesis of pentaacyl *Burkholderia* Lipid A esterified by β-l-Ara4N at the anomeric phosphate **1** and its Ara4N-free counterpart **2** and the biological activity of synthetic compounds at human TLR4**⋅**MD-2 complex.

## Results and Discussion

**Scheme 1:**
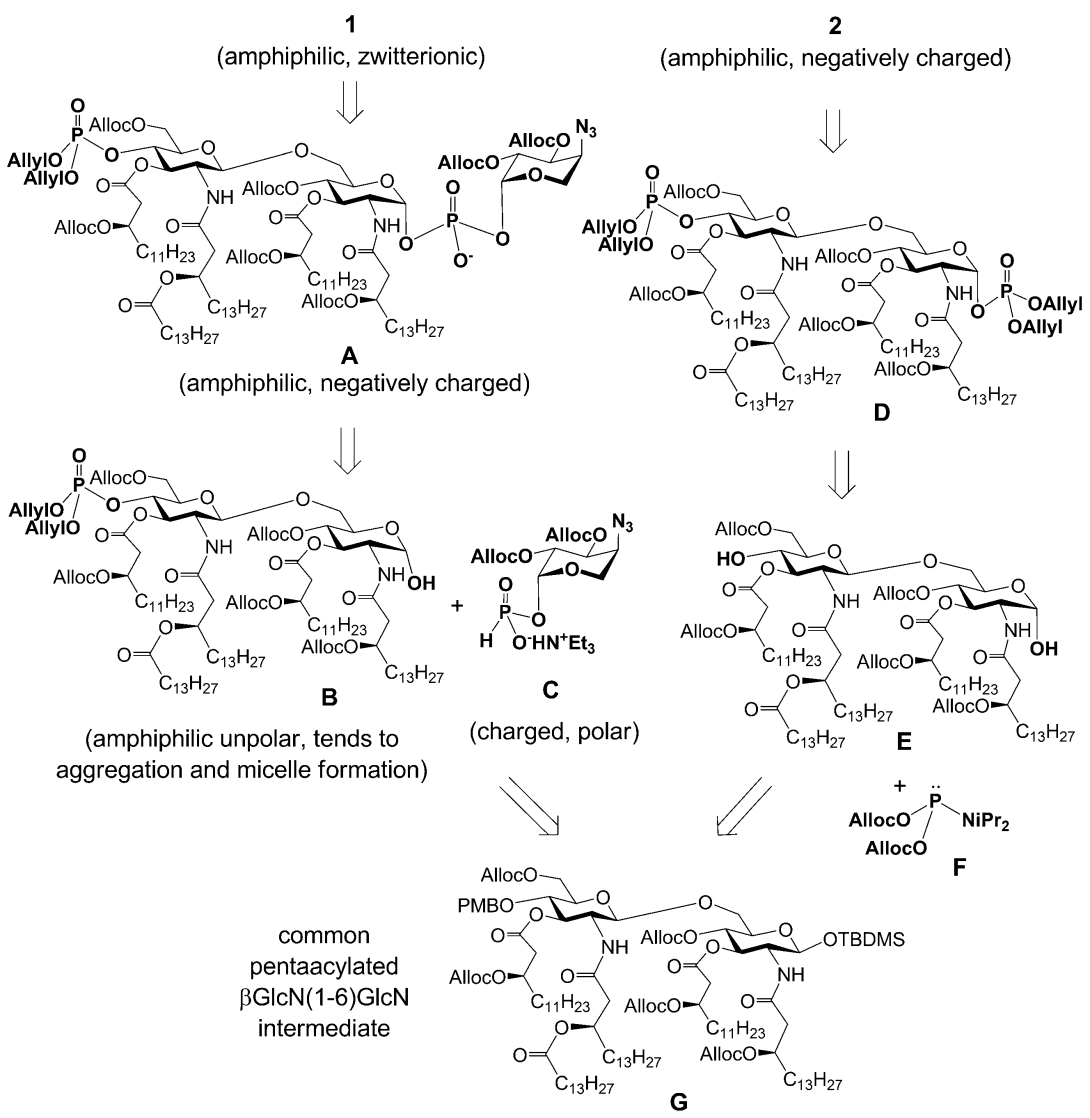
Retrosynthetic analysis of *Burkholderia* Lipid A 1 and 2.

The major challenge in the chemical synthesis of Lipid A substituted by glycosidically linked β-l-Ara4N at 1-phosphate resides in the assembly of an intrinsically labile double glycosyl phosphodiester under simultaneous stereocontrol at two glycosidic centres. We have previously established that the H-phosphonate approach is more advantageous for this purpose compared with the phosphoramidite methodology in regard to higher stereoselectivity and sufficient stability of the intermediate phosphite derivatives (H-phosphonates).[28] Therefore, in our synthetic approach we relied on the initial preparation of the anomerically pure H-phosphonate of 4-azido-protected β-l-Ara4N (**C**), which would then be coupled to the pentaacylated β(1→6) diglucosamine lactol (**B**) (Scheme 1). Because of the presence of a masked amino group in the key trisaccharide phosphodiester (**A**) the application of benzyl protecting groups, which could be cleaved either by hydrogenolysis on [Pd(OH)_2_] in acidic conditions[29, 30] (incompatible with the presence of an acid-labile double glycosyl phosphodiester linkage), or by reduction in Birch conditions (Na/NH_3_),[31] which would lead to the loss of β-acyloxyacyl chains, was avoided. Furthermore, benzyl protections of β-hydroxyl groups in fatty acids were reported to be highly susceptible to air oxidation and easily transformed to benzoyl groups.[32] Allyloxycarbonyl (Alloc) protection, which is decently stable and can be removed under mild neutral conditions was chosen as permanent protecting group both for the Lipid A backbone and for (*R*)-3-hydroxy-fatty acids[33] (Scheme 1). To minimize the number of deprotection steps, an allyl-protected phosphoramidite[34] **F** was used both for the phosphorylation of the diol **E** at O-4′ and O-1 to furnish bisphosphate **D** and for the phosphate instalment at position 4′ in the route to compound **1**. A common orthogonally protected, pentaacylated (by long-chain (*R*)-3-(allyloxycarbonyl)oxyacyl- and (*R*)-3-acyloxyacyl residues) disaccharide **G** served as precursor for both target Lipid A **1** and **2**. High amphiphilicity of the intermediates **A**, **B** and **E** and a combination of peculiar physico-chemical properties in the target amphiphilic zwitterionic Lipid A **1** possessing inherently labile 1,1′-glycosyl phosphodiester linkage imposed additional challenges with respect to the synthesis and final purification.

### Synthesis of β-l-Ara4N H-phosphonate

First, we focused on the stereoselective preparation of the H-phosphonate of β-l-Ara4N. Generally, glycosyl phosphites (H-phosphonates) can be easily obtained from the corresponding hemiacetals by reaction with the activated P^III^ reagents, such as salicylchlorophosphite.[35, 36] It has been previously shown that the anomeric ratio of glycosidic H-phosphonates obtained by reaction with the activated phosphites commonly reflects the configuration of the starting lactol.[37–39] In-situ anomerisation attempts of the 4-azido-protected l-Ara4N lactols provided variable β/α ratios,[40] therefore, we had to rely on a careful attenuation of the protecting group pattern at positions 2 and 3, which would allow fast and uncomplicated isolation of the anomerically pure β-l-Ara4N H-phosphonate. Luckily, among a series of 2,3-di-*O*-protecting groups tested, the Alloc group, which was also applied as a permanent protection for the diglucosamine backbone, provided a very satisfactory outcome.

**Scheme 2:**
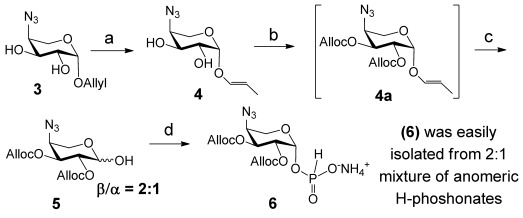
Synthesis of β-l-Ara4N H-phosphonate. a) [Ir^I^(MePPh_2_)_2_(cod)]PF_6_, H_2_, THF, 99 %; b) AllocCl, TMEDA, CH_2_Cl_2_; c) I_2_, H_2_O, 0 °C, 75 % for two steps; d) SalPCl, Py, THF, then Et_3_N, H_2_O, 55 %.

The anomeric allyl group of the known diol **3**[41] was first isomerised into a propenyl group by the action of [Ir^I^(MePPh_2_)_2_(cod)]PF_6_ (cod=1,5-cyclooctadiene) catalyst to give 1-*O*-propenyl glycoside **4** in quantitative yield (Scheme 2). The latter was treated with allyloxycarbonyl chloride in the presence of *N*,*N*,*N′*,*N′*-tetramethylethylenediamine (TMEDA)[42] to furnish **4 a**, which was deprotected at the anomeric position by aqueous iodine-promoted hydrolysis[43] to provide anomeric lactol **5** (β/α=2:1) in 75 % yield. Lactol **5** was subsequently treated with 2-chloro-1,3,2-benzodioxaphosphorin-4-one (salicyl chlorophosphite, SalPCl) in the presence of pyridine to furnish an anomeric mixture (β/α=2:1) of the H-phosphonates. The axial H-phosphonate **6** was readily isolated by a single chromatography on silica gel as ammonium salt in 55 % yield (Scheme 2).

### Synthesis of the common orthogonally protected pentaacylated βGlcN(1→6)αGlcN intermediate

**Scheme 3:**
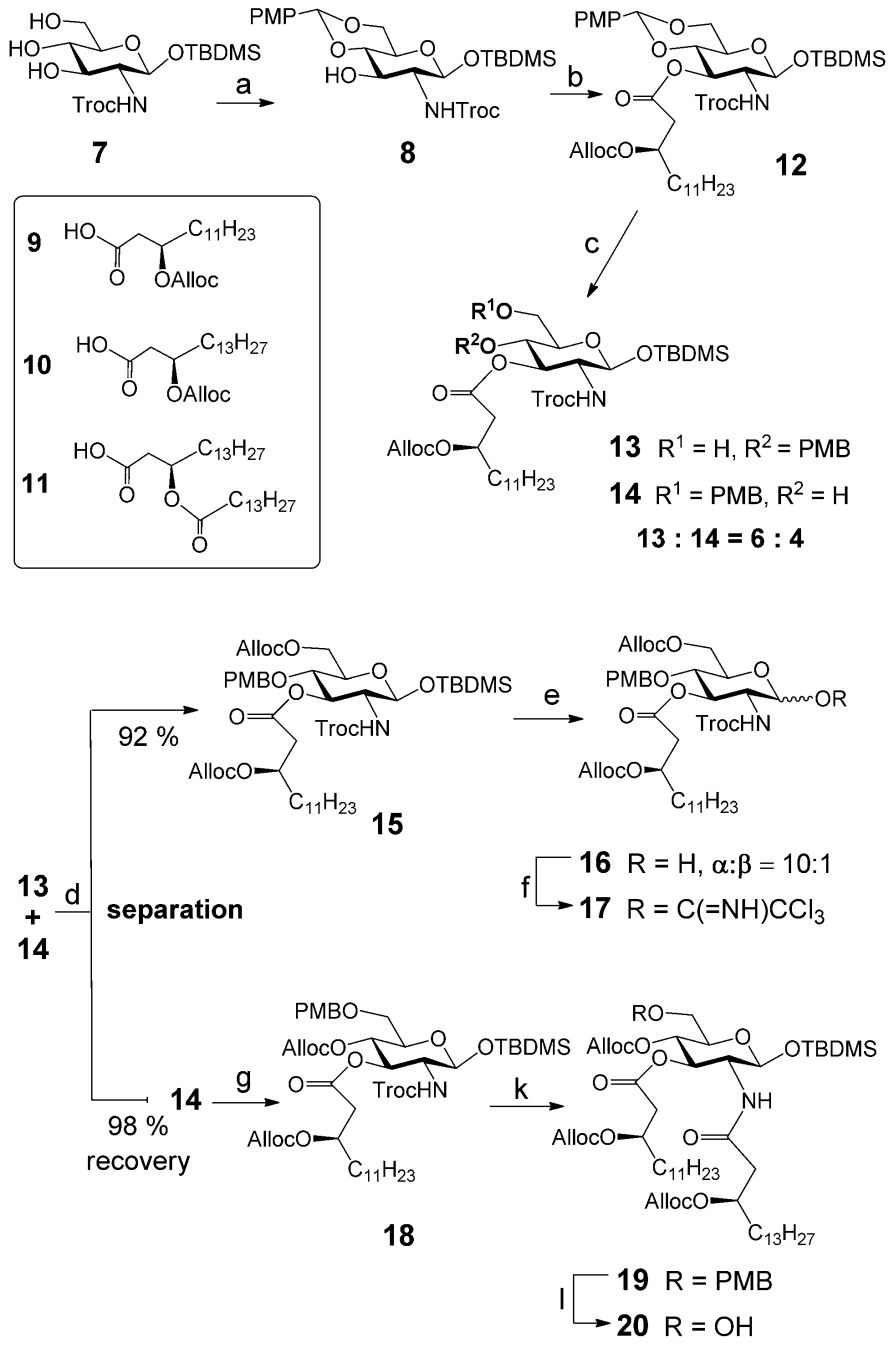
Synthesis of GlcN donor and acceptor molecules. a) Anisaldehyde dimethyl acetal, CSA, CH_3_CN, 75 %; b) 9, DIC, cat. DMAP, CH_2_Cl_2_, 0 °C, 97 %; c) NaCNBH_3_, TMSCl, MeCN, 90 %; d) AllocCl, collidine, CH_2_Cl_2_, 45 °C, 96 %; e) TREAT-HF, NEt_3_, THF, 98 %; f) Cl_3_CCN, Cs_2_CO_3_, CH_2_Cl_2_, 80 %; g) AllocCl, TMEDA, CH_2_Cl_2_, 95 %, k) Zn, dioxane/AcOH, 2:1, then 10, DIC, CH_2_Cl_2_, 0 °C, 82 %; l) TFA, CH_2_Cl_2_, 92 %.

The synthesis of the pentaacylated β(1→6) diglucosamine backbone commenced with the preparation of the GlcN-based donor and acceptor molecules (Scheme 3). To this end, the *p*-methoxybenzylidene acetal **8** was acylated by (*R*)-3-(allyloxycarbonyloxy)tetradecanoic acid **9** in the presence of strictly equimolar amount of diisopropylcarbodiimide (DIC) and catalytic amount of 4-*N*,*N*-dimethylaminopyridine (DMAP) at 0 °C (to avoid a formation of α,β-elimination product at fatty acid),[44] which afforded **12** in nearly quantitative yield. Reductive opening of 4,6-*p*-methoxybenzylidene acetal with sodium cyanoborohydride and trimethylsilyl chloride in acetonitrile furnished a mixture of two isomeric products, 6-OH derivative **13** and its 4-OH regio-isomer **14**. To avoid tedious separation of the co-migrating products, the mixture was subjected to regioselective 6-*O*-protection with allyloxycarbonyl group by the action of AllocCl in the presence of a mild base *sym-*collidine, which transformed compound **13** into 6-*O*-Alloc-protected **15**, whereas the 4-OH derivative **14** was unaffected. The resulting mixture (**14**+**15**) was separated by chromatography to provide **15** in 92 % yield and quantitatively recovered **14**. Orthogonally protected **15** was desilylated at the anomeric position by treatment with triethylamine tris(hydrogenfluoride) (TREAT-HF) buffered by addition of Et_3_N (until pH 6.5) to keep the acid-labile 6-*O*-*p*-methoxybenzyl (PMB) group unaffected. The resulting lactol **16** was transformed into trichloroacetimidate (TCA) donor **17**. The secondary hydroxyl group at C-4 in **14** was treated with AllocCl in the presence of TMEDA to afford fully protected monosaccharide **18**. The trichloroethoxycarbonyl (Troc) group in **18** was reductively cleaved by treatment with Zn in acetic acid/dioxane and the resulting amine was acylated by DIC-activated 3-*O*-Alloc-protected fatty acid **10** to provide **19**. The acceptor **20** was obtained by acidic hydrolysis of the PMB group in **19** with trifluoroacetic acid (TFA) at 0 °C.

**Scheme 4:**
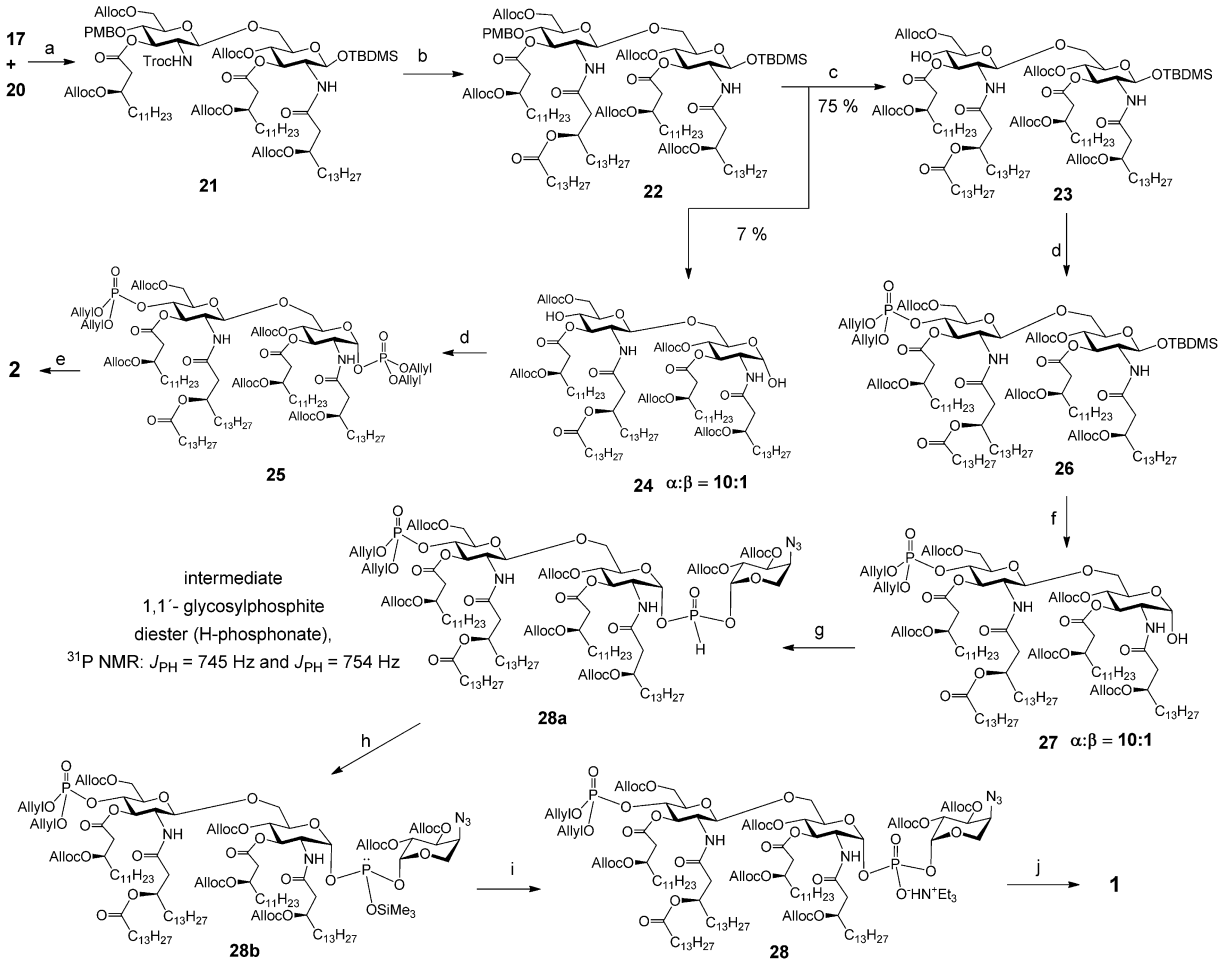
Synthesis of β-l-Ara4N-modified *Burkholderia* Lipid A. a) TMSOTf, CH_2_Cl_2_, −65 °C, MS 4 Å, 90 %; b) Zn, dioxane/AcOH, 2:1 then 11, EDC⋅HCl, CHCl_3_, 0 °C, 67 %; c) TFA, CH_2_Cl_2_, 75 % for 23, 7 % for 24; d) 1) *i*Pr_2_NP(OAllyl)_2_, 1 *H*-tetrazole, CH_3_CN; 2) PNO; 84 % for 25, 93 % for 26; e) [CpRu^IV^(π-C_3_H_5_)(2-quinolinecarboxylato)]PF_6_, CHCl_3_/MeOH, 4:1, 50 %; f) TREAT-HF, THF, 87 %; g) 6, PyNTP, 2,6-lutidine, CH_3_CN/CH_2_Cl_2_, 4:1; h) BSA, NEt_3_; i) CSO, 61 % for three steps; j) 1. PtO_2_, H_2_, toluene/MeOH, 4:1, 2. [CpRu^IV^(π-C_3_H_5_)(2-quinolinecarboxylato)]PF_6_, CHCl_3_/MeOH, 4:1, 57 % for two steps.

Trifluoromethanesulfonic acid (TMSOTf)-promoted glycosylation of **20** by TCA donor **17** at −65 °C furnished β(1→6) disaccharide **21** in 90 % yield (Scheme 4). Reduction of the 2′*N*-Troc group by Zn/AcOH in dioxane followed by *N*-acylation with (*R*)-3-acyloxyacyl fatty acid **11** in the presence of 1-ethyl-3-(3-dimethylaminopropyl) carbodiimide hydrochloride (EDC**⋅**HCl) resulted in orthogonally protected common intermediate **22**.

### Synthesis of Ara4N-modified Lipid A 1 and its non-modified counterpart 2

To differentiate between the PMB group at C-4′ and TBDMS group at C-1 in **22**, a single deprotection step consisting of a treatment with TFA in CH_2_Cl_2_ was applied, which resulted in the fast hydrolysis of 4′-*O*-PMB group and partial acid-catalysed deprotection of the anomeric TBDMS group, to afford, after separation by chromatography on silica gel, the 4′-OH derivative **23** (75 %) and α-lactol **24** (α/β=10:1) having a free OH group at C-4′ (7 %) (Scheme 4). Compound **24** was phosphorylated by reaction with diallyl(*N*,*N-*diisopropyl)phosphoramidite in the presence of a mild acid catalyst 1 *H*-tetrazole and successive oxidation of the intermediate phosphites with 2-(phenylsulfonyl)-3-(3-nitrophenyl)oxaziridine (PNO)[45, 46] to provide anomerically pure 4′,1-bisphosphotriester **25**. A small proportion of the thermodynamically less stable β-GlcN 1-phosphate derivative, which was apparently formed in a phosphitylation reaction of the 10:1 mixture of the anomeric lactols **24**, had obviously hydrolysed upon purification on silica gel.[47] 4′-Hydroxy compound **23** was similarly phosphorylated at O-4′, which resulted in a protected monophosphate **26** in 93 % yield. The TBDMS group at C-1 was cleaved by reaction with TREAT-HF to give anomeric lactol **27** with a high preponderance of α-anomer (α/β=10:1), which was rationalized by a possible stabilization of the axial orientation of the 1-OH group by intramolecular hydrogen bonding with the 2-NH group.[28, 39]

Coupling of lactol **27** (α/β=10:1) to the anomerically pure glycosyl H-phosphonate **6** promoted by 3-nitro-1,2,4-triazol-1-yl-tris(pyrrolidin-1-yl)phosphonium hexafluorophosphate (PyNTP)[48] in the presence of 2,6-lutidine afforded intermediate 1,1′-glycosyl H-phosphonate diester **28 a** (Supporting Information, Figure 1). The progression of the reaction was followed by ^31^P NMR spectroscopy either by performing the H-phosphonate coupling in the NMR tube (CD_3_CN) or by NMR spectroscopic analysis of a small aliquot of the reaction mixture. The reaction is supposed to proceed through formation of the reactive tetracoordinated P^III^-intermediates such as H-pyrophosphonates[49] and nitrotriazol-1-yl-phosphites,[50] which can be detected by ^31^P NMR spectroscopy in the absence of hydroxylic component. Upon shortage of nucleophilic component **27**, activation of Ara4N H-phosphonate **6** resulted in eventual formation of nitrotriazol-1-yl-H-phosphonate **6 a** (Supporting Information, Figure 2), which was detected by ^31^P NMR spectroscopy (*δ*=13.1 and 14.1 ppm, *J*_PH_=652 and 656 Hz, respectively). The formation of the intermediate double anomeric H-phosphonate diester was confirmed by the appearance of the characteristic PH-coupled signals corresponding to (*R_p_*/*S_p_*)-diastereomeric mixture **28 a** (*δ*=7.6 and 8.0 ppm, *J*_P-H_=745 and 754 Hz, respectively; Supporting Information, Figure 3). For the full conversion of lactol **27** into the H-phosphonate diester **28 a**, application of a large excess (up to 10 equivalents, compared with **27**) of the polar, charged H-phosphonate **6** was necessary, which might be explained by the propensity of highly hydrophobic pentaacylated lactol **27** to undergo aggregation in a polar reaction solvent (acetonitrile), which could render the 1-OH group less accessible, necessitating a higher concentration of the activated H-phosphonate **6** for completion of the coupling. The oxidation of **28 a** was performed in anhydrous conditions by first transformation of the H-phosphonate **28 a** (tetra-coordinated) into the phosphite **28 b** (three-coordinated) by treatment with *N*,*O*-bis(trimethylsilyl)acetamide (BSA)[51, 52] in the presence of Et_3_N, followed by oxidation with (1S)-(+)-(10-camphorsulfonyl)- oxaziridine (CSO)[53] to furnish 1,1′-glycosyl phosphodiester **28**. The anomeric configuration about αGlcN(1→P←1)βAra4N linkage was confirmed by the corresponding coupling constants *J*_H1,H2_=3.3 and *J*_H1,P_=7.1 Hz for α-D-*gluco*- and *J*_H1,H2_=3.5 and *J*_H1,P_=7.4 Hz for β-l-*arabino*-anomers, respectively.

Initial attempts to perform the H-phosphonate coupling between **27** and **6** under standard conditions, which were successfully employed for the preparation of glycosyl phosphodiesters (activation with pivaloyl chloride followed by oxidation with aqueous I_2_ at reduced temperature),[38, 39, 54] did not result in the formation of the target trisaccharide **28**. This could be explained by both a predisposition of the *N*-acylated GlcN derivatives to formation of oxazolines in the presence of an excess of chloroanhydrides[55] and the instability of 1,1′-glycosylphosphite diester in the harsh conditions of aqueous I_2_-oxidation.

Total cleavage of Alloc-[56] and Allyl-protecting groups[57] in **25** and **28** was smoothly performed with a catalytic amount of [CpRu^IV^(π-C_3_H_5_)(2-quinolinecarboxylato)]PF_6_ (Cp=cyclopentadienyl) complex[58] in MeOH at RT under neutral pH. This procedure was perfectly compatible with the presence of a labile double glycosyl phosphodiester linkage, which was not affected under applied conditions and no hydrolysis products were observed (according to ^1^H NMR spectroscopy and HRMS of the reaction mixture, data not shown). Finally, the azido group at Ara4N moiety was reduced by hydrogenation on PtO_2_ in toluene/methanol, 4:1. The target amphiphilic Lipid A **1** and **2** were purified by using size exclusion chromatography on Sephadex SX1 and Sephadex LH-20 supports, which allowed for separation from ruthenium oxide anion contaminations (RuO_4_^−^: yellowish, RuO_4_^2−^: pink) and by anion-exchange chromatography on DEAE cellulose by elution with CHCl_3_-MeOH-aqueous triethylammonium acetate (TEAA) buffer, which simultaneously afforded a single salt form at the phosphates (triethylammonium salt).[59] The zwitterionic phosphodiester **1** was easily hydrolysed at higher concentrations of TEAA buffer (pH 7) giving rise to the phosphate **2** and free Ara4N, which could be explained by the intramolecular nucleophilic attack of the amino group at C-4′′ of Ara4N on the phosphorus atom.

### Biological evaluation

**Figure 2:**
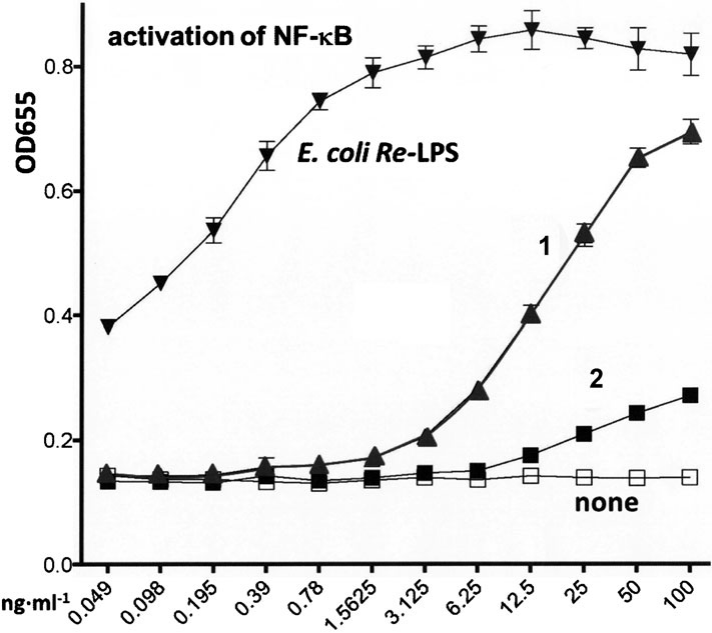
Dose-dependent activation of TLR4 signalling in hTLR4/hMD-2/hCD14 transfected HEK293 cells (HEK-Blue) by Lipid A 1 and 2 compared to *E. coli* Re-LPS.

The ability of synthetic Ara4N-modified *Burkholderia* Lipid A **1** and its non-modified counterpart **2** to initiate the pro-inflammatory signaling was first assessed in the hTLR4/hMD-2/CD14 transfected human embryonic kidney (HEK)293 cells (HEK-Blue) through monitoring the activation of NF-κB signaling by measuring the induction of secreted alkaline phosphatase (SEAP) whose expression is under control of an NF-κB-responsive promoter. The Ara4N-modified Lipid A **1** was considerably less efficient than *E. coli Re*-LPS, but showed a significantly more potent NF-κB stimulation profile reaching 80 % of the activity of *E. coli Re*-LPS at a concentration 100 ng mL^−1^, compared with the 1,4′-bisphosphate Lipid A **2**, which was largely inactive (Figure 2).

**Figure 3:**
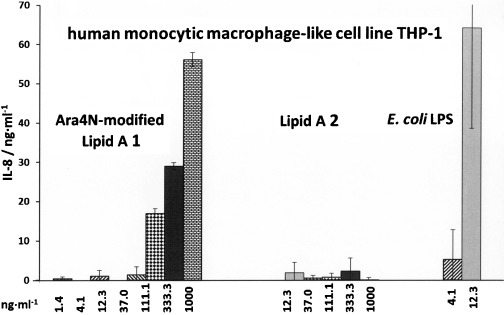
Dose-dependent expression of IL-8 induced by synthetic *Burkholderia* Lipid A 2 and Ara4N-modified Lipid A 1 in human macrophage cell line THP-1 compared to *E. coli* O111:B4 LPS.

Next, Lipid A **1** and **2** were examined for the propensity to initiate the expression of interleukin-8 (IL-8) in the human monocytic macrophage cell line THP-1, which expresses cell surface receptors MD-2, CD14 and TLR4. Although the pentaacyl bisphosphate Lipid A **2** revealed no activity at all, the Ara4N-modified Lipid A **1** was efficient in induction of the expression of IL-8, though again at significantly higher doses than *E. coli* LPS (Figure 3).

Evidently, positively charged Ara4N modification at the anomerically linked phosphate of *Burkholderia* Lipid A **1** plays an important role in enhancement of the pro-inflammatory signalling of otherwise inactive long-chain pentaacylated Lipid A **2**. Ara4N might actively participate in the dimerization interface of two TLR4/MD-2/Lipid A complexes, perhaps, by establishing additional ionic contacts between its 4-amino group and specific amino acid residues on the second TLR4*, which results in the augmented activation. Furthermore, dampened pro-inflammatory signaling induced by pentaacylated Ara4N-Lipid A **1** compared with hexaacyl *E. coli* LPS/Re-LPS is presumably related to its underacylated structure. High *E. coli* LPS-like activity of the BCC bacterial LOS isolates could be also associated with the presence of the core-sugar region attached at position 6′ of Lipid A,[7] leading to enhanced TLR4 activation, which has been already shown for the underacylated LOS variants of other bacterial species.[60] Moreover, glycosylation of Lipid A by negatively charged 3-deoxy-D-*manno*-octulosonic acid (Kdo) residues (as in LOS isolates) can also result in the boosted pro-inflammatory signaling.[10, 61, 62]

## Conclusion

The first synthesis of 4-amino-4-deoxy-β-l-arabinose-modified Lipid A corresponding to a partial structure of pentaacylated *Burkholderia cepacia* complex LPS has been achieved. The H-phosphonate approach has been applied for the assembly of a double glycosyl phosphodiester linkage involving the anomeric centres of α-D-GlcN and β-l-Ara4N. Phosphonium reagent (PyNTP)-mediated coupling and non-aqueous oxaziridine (CSO)-promoted oxidation of three-coordinated 1,1′-glycosyl phosphite-triester intermediate were found superior to conventional coupling conditions (PivCl, aqueous I_2_ oxidation). Permanent Alloc protecting groups both on a trisaccharide backbone of Lipid A **1** and on the β-hydroxyacyl chains were efficiently cleaved simultaneously with allyl-protecting groups on the phosphates by application of an Ru^IV^ complex, preserving the labile 1,1′-glycosylphosphodiester linkage intact. This convergent approach could be effectively employed for the synthesis of other relevant Lipid A structures entailing glycosyl phosphate-linked aminosugar substitution. We have shown that the long-chain pentaacyl *Burkholderia* Lipid A **2** was largely inactive in eliciting the pro-inflammatory cytokines in human cells, whereas Ara4N-modified Lipid A **1** induced considerable pro-inflammatory innate immune signalling, though at higher (micro-molar) concentrations compared with *E. coli* LPS. Thus, biological assays revealed immuno-modulatory potential of the Ara4N modification and showed boosted pro-inflammatory activity of *Burkholderia* Lipid A esterified by β-l-Ara4N at the anomerically-linked phosphate. This finding is of importance for understanding of the pro-virulent strategy of the CF adapted BCC species and for the development of advanced CF-related therapeutics.

## Experimental Section

### General methods

Reagents and solvents were purchased from commercial suppliers and used without further purification unless otherwise stated. Dichloromethane was distilled from CaH_2_ and stored over activated 4 Å molecular sieves. THF was distilled over Na/benzophenone directly before use. Other solvents were dried by storage over activated molecular sieves for at least 48 h prior to use (toluene (4 Å), acetonitrile (3 Å) and DMF (3 Å)). Residual moisture was determined by colorimetric titration on a Mitsubishi CA-21 Karl Fischer apparatus and did not exceed 20 ppm for dry solvents. Reactions were monitored by TLC performed on silica gel 60 F254 HPTLC pre-coated glass plates with a 25 mm concentration zone (Merck). Spots were visualized by UV light followed by dipping into a H_2_SO_4_/*p*-anisaldehyde solution or a ninhydrin/EtOH solution and subsequent charring at 250 °C. Solvents were removed under reduced pressure at <40 °C. Preparative MPLC was performed on silica gel 60 (230–400 mesh, Merck). Size exclusion chromatography was performed on Sephadex LH20 (BioRad) or Sephadex SX1 (BioRad) supports. NMR spectra were recorded at 25 °C on a Bruker Avance III 600 spectrometer (^1^H at 600.22 MHz; ^13^C at 150.92 MHz; ^31^P at 242.97 MHz) or on Bruker DPX 400 spectrometer (^1^H at 400.13 MHz; ^13^C at 100.61 MHz; ^31^P at 161.68 MHz) or on a Bruker Avance 300 spectrometer (^31^P at 161.68 MHz) using standard Bruker NMR software. Chemical shifts are reported in ppm; ^13^C NMR spectra are referenced to the residual solvent signal (77.00 ppm for CDCl_3_, 49.00 ppm for MeOD). ^31^P NMR Spectra in CDCl_3_ and CD_3_CN are referenced to external triphenylphosphine. For the trisaccharides the NMR signals of the distal GlcN moiety are indicated by primes, the signals of Ara4N moiety are indicated by double primes. HPLC-LRMS was performed by injections of 0.01–0.1 % CH_3_CN solutions into a Shimadzu LC-10AD VP system equipped with two gradient pumps, degasser, a Shimadzu LCMS 2020 detector and an AllTech 3300 ELSD detector. Analytes were eluted over a Phenomenex Jupiter 5μ C4 300A column using linear gradients H_2_O (0.1 % HCOOH)→CH_3_CN (0.1 % HCOOH). High-resolution mass spectrometry (HRMS) was carried out from acetonitrile solutions (1–10 mg L^−1^) on LC-TOF MS (Agilent 1200SL HPLC and Agilent 6210 ESI-TOF, Agilent Technologies). The data were analysed by using Agilent Mass Hunter Software. MALDI-TOF was performed using a Bruker Autoflex Speed TOF-TOF instrument with 6-aza-2-thiothymine (ATT) as matrix. ESI-MS was performed on a maXis Q-TOF mass spectrometer (Bruker, Bremen, Germany) using ionization in positive ion mode on an ESI source with standard spray settings and 400–3500 *m*/*z* scan range, 100 μs transfer time and 10 μs pre-pulse storage. The data were processed by using standard Results Bruker Data Analysis 4.0 software. Optical rotation was measured on a PerkinElmer 243 B polarimeter, equipped with a Haake water circulation bath and a Haake D1 immersion circulator for temperature control of the measuring cell. 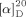 Values are given in units of deg dm^−1^ cm^3^ g^−1^.

**Ammonium 2,3-di-*O*-allyloxycarbonyl-4-azido-4-deoxy-β-l-arabino-pyranosyl hydrogenphosphonate (6)**: A solution of **5** (170 mg, 0.50 mmol) in dry THF (10 mL) was added to a stirred solution of salicylchlorophosphite (304 mg; 1.5 mmol) and pyridine (0.48 mL; 6 mmol) in dry THF (5 mL) at 0 °C under Ar. The reaction mixture was stirred for 4 h at RT. Then, triethylamine (0.5 mL) and water (0.5 mL) were added and the mixture was stirred for 15 min. The reaction mixture was diluted with chloroform (100 mL) and washed with 1 m aqueous TEAB buffer (2×30 mL). The aqueous phase was extracted with chloroform (2×50 mL) and the combined organic phases were dried by filtration over cotton. The solution was diluted with toluene (50 mL) and concentrated. The residue was purified by chromatography on silica gel (chloroform/methanol/25 % aq. NH_4_OH, 16:4:0.5) to give **6** as a solid. Yield: 112 mg (55 %). *R*_f_=0.18 (CHCl_3_/MeOH/25 % aq.NH_4_OH, 16:4:0.5); 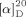
=+108 (*c*=0.85 in chloroform); ^1^H NMR (600 MHz, CDCl_3_, TMS): *δ*=6.83 (d, *J*_P−H_=649 Hz, 1 H; H-P), 5.96–5.87 (m, 2 H; 2×=CH, Alloc), 5.78 (dd, *J*_P,1_=8.8 Hz, *J*_1,2_=3.2 Hz, 1 H; H-1), 5.38–5.32 (m, 2 H; =CH_2_ Alloc *trans*), 5.30–5.25 (m, 3 H; =CH_2_ Alloc *cis*, H-3), 5.04 (dd, *J*_3,2_=10.3 Hz, 1 H; H-2), 4.70–4.60 (m, 4 H; O-CH_2_-Alloc), 4.25 (m, 1 H; H-4), 4.21 (ad, *J*_5a,b_=12.0 Hz, 1 H; H-5a), 3.84 ppm (ad, 1 H; H-5b); ^13^C NMR (151 MHz, CDCl_3_): *δ*=154.05, 153.80 (C=O Alloc), 131.21, 131.07 (=CH Alloc), 119.38, 119.19 (=CH_2_ Alloc), 91.67 (d, *J*=4.4 Hz, C-1), 72.51 (C-3), 71.67 (d, *J*=6.8 Hz, C-2), 69.27, 69.16 (OCH_2_ Alloc), 61.46 (C-5), 59.69 ppm (C-4); ^31^P NMR (243 MHz, CDCl_3_): *δ*=2.28 ppm; HRMS (−ESI-TOF): *m*/*z* calcd for C_13_H_17_N_3_O_10_P: 406.0657 [*M*−H]^−^; found: 406.0656.

***tert-*****Butyldimethylsilyl 4-*O*-allyloxycarbonyl-6-*O*-[6-*O*-allyloxycarbonyl-3-*O*-[(*R*)-3-(allyloxycarbonyloxy)tetradecanoyl]-2-deoxy-4-*O*-*p*-methoxybenzyl-2-(2,2,2-trichloroethoxycarbonylamino)-β-D-glucopyranosyl]2-[(*R*)-3-(allyloxycarbonyloxy)hexadecanoylamino]-3-*O*-[(*R*)-3-(allyloxycarbonyloxy)tetradecanoyl]-2-deoxy-β-D-glucopyranoside (21)**: A solution of **17** (535 mg, 0.53 mmol) and **20** (461 mg, 0.45 mmol) in dry dichloromethane (40 mL) was stirred with powdered activated molecular sieves (4 Å, 50 mg) for 2 h under Ar. The reaction mixture was cooled to −65 °C and a solution of TMSOTf (0.055 m, 1 mL, 0.055 mmol) in dry CH_2_Cl_2_ was added dropwise. The reaction mixture was stirred for 1.5 h at −65 °C, the temperature was allowed to rise to −30 °C and a solution of triethylamine (50 μL) in dry CH_2_Cl_2_ (0.5 mL) was added. The mixture was stirred for 10 min, warmed up to RT, diluted with dichloromethane (100 mL) and filtered over a pad of Celite. The filtrate was diluted with CH_2_Cl_2_ (100 mL) and washed with sat. aq. NaHCO_3_ (2×50 mL) and water (50 mL). The organic phase was dried over Na_2_SO_4_, filtered and concentrated. The residue was purified by MPLC (toluene/ethyl acetate, 9:1→7:1) to give **21** (765 mg, 90 %) as a syrup. *R*_f_=0.40 (toluene/ethyl acetate, 5:1); 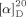
=+10 (*c*=1.2 in chloroform); ^1^H NMR (600 MHz, CDCl_3_, TMS): *δ*=7.19 (m, 2 H; *m*-CH Ar), 6.86 (m, 2 H; *o*-CH Ar), 5.98–5.86 (m, 5 H; 5×=CH Alloc), 5.84 (d, *J*_2,NH_=8.4 Hz, 1 H; NH), 5.44 (d, *J*=8.8 Hz, 1 H; N′H), 5.41–5.21 (m, 12 H; 5×=CH_2_ Alloc, H-3, H-3′), 5.07 (m, 1 H; β-CH^acyl^), 4.99 (m, 1 H; β-CH^acyl^), 4.96–4.92 (m, 2 H; β-CH^acyl^, H-1), 4.80 (t, *J*_3,4_=*J*_5,4_=9.5 Hz, 1 H; H-4), 4.77 (d, *J*_gem_=12.0 Hz, 1 H; OCH_2a_ Troc), 4.70 (d, 1 H; OCH_2b_ Troc), 4.66–4.53 (m, 11 H; 5×OCH_2_ Alloc, OCH_2a_ PMB), 4.49 (d, *J*_2′,1′_=8.3 Hz, 1 H; H-1′), 4.45 (d, *J*_gem_=10.9 Hz, 1 H; OCH_2b_ PMB), 4.40 (dd, *J*_6a′,b′_=11.5 Hz, *J*_5′,6a′_=1.9 Hz, 1 H; H-6a′), 4.22 (dd, *J*_5′,6b′_=4.9 Hz, 1 H; H-6b′), 3.95 (dd, *J*_6a,b_=11.5 Hz, *J*_5,6a_=2.1 Hz, 1 H; H-6a), 3.79 (s, 3 H; OMe), 3.68–3.63 (m, 2 H; H-2, H-5), 3.62 (at, *J*_5′,4′_=*J*_3′,4′_=9.3 Hz, 1 H; H-4′), 3.59–3.51 (m, 3 H; H-6b, H-2′, H-5′), 2.62 (dd, *J*_gem_=16.4 Hz, *J*_vic_=8.1 Hz, 1 H; α-CH_a_^acyl^), 2.56 (d, *J*=6.2 Hz, 2 H; α-CH_2_^acyl^), 2.54 (dd, *J*_vic_=4.6 Hz, 1 H; α-CH_b_^acyl^), 2.48 (dd, *J*_gem_=15.2 Hz, *J*_vic_=6.4 Hz, 1 H; α-CH_a_^acyl^), 2.34 (dd, *J*_vic_=5.6 Hz, 1 H; α-CH_b_^acyl^), 1.70–1.54 (m, 6 H; γ-CH_2_^acyl^), 1.38–1.18 (m, 58 H; CH_2_^acyl^), 0.90–0.84 (m, 18 H; ω-CH_3_^acyl^, *t*Bu), 0.10, 0.08 ppm (2 s, 6 H; 2×Si-Me); ^13^C NMR (150 MHz, CDCl_3_): *δ*=169.97, 169.87, 169.29 (2×CO, CONH^acyl^), 159.71 (C_q_ PMB), 154.82, 154.78, 154.64, 154.50, 154.28 (C_q_ Alloc, C_q_ Troc), 131.86, 131.75, 131.73, 131.65, 131.51 (5×=CH Alloc), 130.06 (*m*-CH PMB), 129.50 (*p*-C_q_ PMB), 119.50, 119.25, 119.14, 119.06, 118.92 (5×=CH_2_ Alloc), 114.16 (o-CH PMB), 101.05 (C-1′), 95.82 (C-1), 94.14 (CCl_3_ Troc), 75.48 (β-CH^acyl^), 75.24 (C-4′), 75.09 (C-3′), 74.70 (CH_2_ Troc), 74.61, 74.57 (β-CH^acyl^), 74.40 (CH_2_ PMB), 73.22 (C-5′), 72.98 (C-4), 72.82 (C-5), 72.47 (C-3), 69.24, 68.85, 68.60 (OCH_2_ Alloc), 68.08 (C-6), 66.12 (C-6′), 57.17 (C-2), 56.65 (C-2′), 55.41 (OMe), 41.48, 38.95, 38.63 (α-CH_2_^acyl^), 34.28, 34.13, 33.99 (γ-CH_2_^acyl^), 32.07, 29.85, 29.84, 29.81, 29.79, 29.74, 29.70, 29.69, 29.64, 29.57, 29.55, 29.51 (CH_2_^acyl^), 25.79 (CH_3_ *t*Bu), 25.28, 25.17, 25.10, 22.83 (CH_2_^acyl^), 17.98 (C_q_ *t*Bu), 14.25 (ω-CH_3_^acyl^), −3.82, −4.87 ppm (Si-CH_3_); HRMS (^+^ESI-TOF): *m*/*z* calcd for C_93_H_149_Cl_3_N_2_O_28_Si: 1875.9204 [*M*+H]^+^; found: 1875.9241.

***tert*****-Butyldimethylsilyl 4-*O*-allyloxycarbonyl-6-*O*-(6-*O*-allyloxycarbonyl-3-*O*-[(*R*)-3-(allyloxycarbonyloxy)tetradecanoyl]-2-deoxy-4-*O*-*p*-methoxybenzyl-2-[(*R*)-3-(tetradecanoyloxy)hexadecanoylamino]-β-D-glucopyranosyl)-2-[(*R*)-3-(allyloxycarbonyloxy)hexadecanoylamino]-3-[(*R*)-3-(allyloxycarbonyloxy)-tetradecanoyl]-2-deoxy-β-D-glucopyranoside (22)**: Zn dust (10 μm, 1.28 g, 19.55 mmol) was added to a stirred solution of **21** (367 mg, 0.20 mmol) in dioxane/acetic acid (2:1, 5 mL) and dispersed by ultrasonic treatment every 20 min. The reaction was stirred for 90 min and filtered over a pad of Celite. The filtrate was diluted with ethyl acetate (150 mL) and washed with water (50 mL) and sat. aq. NaHCO_3_ (3×50 mL) until the pH of organic phase reached 7.5. The organic phase was dried over Na_2_SO_4_, filtered and concentrated. The residue was dried by co-evaporation with dry toluene (3×15 mL) and re-dissolved in dry chloroform (2 mL). A solution of fatty acid **11** (188 mg, 0.39 mmol) in dry chloroform (2 mL) and a solution EDC**⋅**HCl (75 mg, 0.39 mmol) in dry chloroform (1 mL) were added to this stirred solution at 0 °C under Ar. The stirring was continued for 48 h at 4 °C, the reaction mixture was diluted with chloroform (150 mL) and washed with 10 % aq. citric acid (25 mL) and sat. aq. NaHCO_3_ (25 mL).The organic phase was dried over Na_2_SO_4_, filtered and concentrated. The residue was purified by MPLC (toluene/ethyl acetate, 1:0→8:1) to give **22** (283 mg, 67 %) as a syrup. *R*_f_=0.39 (toluene/ethyl, acetate 5:1); 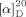
=+12 (*c*=1 in chloroform); ^1^H NMR (600 MHz, CDCl_3_, TMS): *δ*=7.19 (m, 2 H; *m*-CH-Ar), 6.85 (m, 2 H; *o*-CH-Ar), 6.04 (d, *J*=8.5 Hz, 1 H; N′H), 5.98–5.85 (m, 6 H; 5×=CH Alloc, NH), 5.40–5.22 (m, 11 H; 5×=CH_2_ Alloc, H-3), 5.22 (dd, *J*_4′,3′_=8.6 Hz, *J*_2′_,_3′_=10.3 Hz, 1 H; H-3′), 5.14–5.06 (m, 2 H; 2×β-CH^acyl^), 5.02–4.93 (m, 2 H; 2×β-CH^acyl^), 4.96 (d, *J*_2,1_=7.7 Hz, 1 H; H-1), 4.78 (t, *J*_3,4_=*J*_5,4_=9.5 Hz, 1 H; H-4), 4.67–4.49 (m, 11 H; 5×OCH_2_ Alloc, OCH_2a_ PMB), 4.51 (d, *J*_2′,1′_=8.3 Hz, 1 H; H-1′), 4.42 (d, *J*_gem_=10.7 Hz, 1 H; OCH_2b_ PMB), 4.39 (dd, *J*_6a′,b′_=11.6 Hz, *J*_5′,6a′_=2.0 Hz, 1 H; H-6a′), 4.19 (dd, *J*_5′,6b′_=5.0 Hz, 1 H; H-6b′), 3.91 (dd, *J*_6a,b_=11.8 Hz, *J*_5,6a_=2.3 Hz, 1 H; H-6a), 3.79 (s, 3 H; OMe), 3.74 (ddd, 1 H; H-2′), 3.67 (ddd, *J*_6b,5_=4.9 Hz, 1 H; H-5), 3.62 (ddd, *J*_3,2_=10.5 Hz, 1 H; H-2), 3.58 (t, *J*_5′,4′_=9.6 Hz, 1 H; H-4′), 3.57 (dd, 1 H; H-6 b), 3.53 (ddd, 1 H; H-5′), 2.62 (d, *J*=6.3 Hz, 2 H; α-CH_2_^acyl^), 2.56 (d, *J*=6.1 Hz, 2 H; α-CH_2_^acyl^), 2.49–2.42 (m, 2 H; α-CH_2_^acyl^), 2.40–2.27 (m, 4 H; α-CH_2_^acyl^), 1.71–1.53 (m, 10 H; 4×γ-CH_2_^acyl^, β-CH_2_^acyl^), 1.38–1.11 (m, 100 H; CH_2_^acyl^), 0.92–0.81 (m, 24 H; ω-CH_3_^acyl^, *t*Bu), 0.10, 0.07 ppm (2s, 6 H; 2×Si–Me); ^13^C NMR (150 MHz, CDCl_3_, TMS): *δ*=173.88, 170.02, 169.95, 169.80, 169.27 (CO, CONH), 159.67 (C_q_ PMB), 154.84, 154.76, 154.59, 154.50, 154.39 (C_q_ Alloc), 131.88, 131.78, 131.75, 131.70, 131.51 (5×=CH Alloc), 130.09 (*m*-CH PMB), 129.60 (*p*-C_q_ PMB), 119.54, 119.16, 119.13, 119.03, 118.87 (5×=CH_2_ Alloc), 114.13 (*o*-CH PMB), 101.11 (C-1′), 95.71 (C-1), 75.48 (β-CH^acyl^), 75.32 (C-3′), 75.27 (C-4′), 74.71, 74.59 (β-CH^acyl^), 74.29 (OCH_2_ PMB), 73.24 (C-4), 73.19 (C-5′), 72.72 (C-5), 72.66 (C-3), 70.85 (β-CH^acyl^), 69.26, 68.81, 68.58, 68.56, 65.53 (5×OCH_2_ Alloc), 68.30 (C-6), 66.29 (C-6′), 57.23 (C-2), 55.39 (OMe), 54.85 (C-2′), 41.43, 38.92, 38.62, 34.66 (α-CH_2_^acyl^), 34.42, 34.26, 34.17, 32.07 (γ-CH_2_^acyl^, β-CH_2_^acyl^), 32.07, 29.91, 29.88, 29.87, 29.85, 29.83, 29.81, 29.77, 29.75, 29.73, 29.72, 29.70, 29.63, 29.62, 29.59, 29.53, 29.51, 29.43 (CH_2_^acyl^), 25.79 (CH_3_ *t*Bu), 25.48, 25.28, 25.24, 25.15, 25.12, 22.83 (CH_2_^acyl^),17.98 (C_q_ *t*Bu), 14.24 (ω-CH_3_^acyl^), −3.84, −4.91 ppm (Si-CH_3_); MALDI-TOF: *m*/*z* calcd for C_120_H_204_N_2_O_29_Si+C_4_H_5_N_3_OS (ATT): 2308.447 [*M*+ATT]^−^; found: 2308.392 (ATT=6-Aza-2-thiothymine).

***tert-*****Butyldimethylsilyl 4-*O*-allyloxycarbonyl-6-*O*-(6-*O*-allyloxycarbonyl-3-*O*-[(*R*)-3-(allyloxycarbonyloxy)tetradecanoyl]-2-deoxy-2-[(*R*)-3-(tetradecanoyloxy)hexadecanoylamino]-β-D-glucopyranosyl)-2-[(*R*)-3-*O*-(allyloxycarbonyloxy)hexadecanoylamino]-3-*O*-[(*R*)-3-(allyloxy-carbonyloxy)tetradecanoyl]-2-deoxy-β-D-glucopyranoside (23) and 4-*O*-allyloxycarbonyl-6-*O*-(6-*O*-allyloxycarbonyl-3-*O*-[(*R*)-3-(allyloxycarbonyloxy)tetradecanoyl]-2-deoxy-2-[(*R*)-3-(tetradecanoyloxy)hexadecanoylamino]-β-D-glucopyranosyl)-2-[(*R*)-3-*O*-(allyloxycarbonyloxy)hexadecanoylamino]-3-*O*-[(*R*)-3-(allyloxycarbonyloxy)tetradecanoyl]-2-deoxy-D-glucopyranose (24)**: Trifluoroacetic acid (2.17 mL, 28.20 mmol) was added to a stirred solution of **22** (679 mg, 0.31 mmol) in dry CH_2_Cl_2_ (20 mL). The reaction was stirred for 15 min at RT, diluted with CH_2_Cl_2_ (200 mL) and washed with sat. aq. NaHCO_3_ (2×50 mL). The organic phase was dried over Na_2_SO_4_, filtered and concentrated. The residue was purified by MPLC (toluene/ethyl acetate, 5:1→3:2) to afford **23** (480 mg, 75 %) as a syrup and **24** (43 mg, 7 %) as a syrup. Compound **23**: *R*_f_=0.45 (toluene/ethyl acetate 3:1); 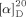
=+4 (*c*=0.5 in chloroform); ^1^H NMR (600 MHz, CDCl_3_, TMS): *δ*=6.01 (d, *J*=8.2 Hz, 1 H; N′H), 5.97–5.88 (m, 5 H; 5×=CH Alloc), 5.87 (d, *J*=8.5 Hz, 1 H; NH), 5.39–5.24 (m, 11 H; 5×=CH_2_ Alloc, H-3), 5.13–5.06 (m, 1 H; β-CH^acyl^), 5.09 (dd, *J*_4′,3′_=8.3 Hz, *J*_2′_,_3′_=10.4 Hz, 1 H; H-3′), 5.03–4.93 (m, 3 H; 3×β-CH^acyl^), 4.97 (d, *J*_2,1_=7.8 Hz, 1 H; H-1), 4.80 (at, *J*_3,4_–*J*_5,4_=9.5 Hz, 1 H; H-4), 4.66–4.57 (m, 10 H; 5×OCH_2_ Alloc), 4.57 (d, *J*_2′,1′_=8.2 Hz, 1 H; H-1′), 4.48 (dd, *J*_6a′,b′_=11.7 Hz, *J*_5′,6a′_=1.9 Hz, 1 H; H-6a′), 4.35 (dd, *J*_5′,6b′_=5.0 Hz, 1 H; H-6b′), 3.94 (dd, *J*_6a,b_=11.6 Hz, *J*_5,6a_=2.3 Hz, 1 H; H-6a), 3.71 (ddd, 1 H; H-2′), 3.68 (ddd, *J*_6b,5_=4.9 Hz, 1 H; H-5), 3.62 (ddd, *J*_3,2_=10.5 Hz, 1 H; H-2), 3.58 (dd, 1 H; H-6b), 3.59–3.51 (m, 2 H; H-4′, H-5′), 3.16 (d, *J*=3.7 Hz, 1 H; OH), 2.66 (dd, *J*_vic_=8.3 Hz, *J*_gem_=15.3 Hz, 1 H; α-CH_2a_^acyl^), 2.58 (dd, *J*_vic_=3.4 Hz, 1 H; α-CH_2b_^acyl^), 2.57 (ad, *J*_vic_=6.2 Hz, 2 H; α-CH_2_^acyl^), 2.49–2.25 (m, 6 H; α-CH_2_^acyl^, α-CH_2_^acyl^), 1.74–1.51 (m, 10 H; 4×γ-CH_2_^acyl^, β-CH_2_^acyl^), 1.40–1.11 (m, 100 H; CH_2_^acyl^, CH_2_^acyl^), 0.91–0.81 (m, 24 H; ω-CH_3_^acyl^, ω-CH_3_^acyl^, *t*Bu), 0.11, 0.08 ppm (2 s, 6 H; 2×Si-Me); ^13^C NMR (151 MHz, CDCl_3_): *δ*=173.72, 171.20, 169.94, 169.78, 169.28 (CO, CONH), 155.21, 155.12, 154.76, 154.49, 154.40 (C_q_ Alloc), 131.87, 131.72, 131.63, 131.49, 131.42 (5×=CH Alloc), 119.63, 119.43, 119.25, 119.18, 118.89 (5×=CH_2_ Alloc), 101.10 (C-1′), 95.69 (C-1), 76.26 (C-3′), 75.76, 75.45, 74.60 (3×β-CH^acyl^), 73.87 (C-5′), 73.22 (C-4), 72.64, 72.62 (C-3, C-5), 70.94 (β-CH^acyl^), 69.29 (OCH_2_ Alloc), 69.06 (C-4′), 68.89, 68.85, 68.60, 68.57 (4×OCH_2_ Alloc), 68.23 (C-6), 66.63 (C-6′), 57.27 (C-2), 54.46 (C-2′), 41.51, 41.44, 39.99, 38.61 (4×α-CH_2_^acyl^), 34.81 (α-CH_2_^acyl^), 34.64, 34.29, 34.27, 32.02 (γ-CH_2_^acyl^, β-CH_2_^acyl^), 32.08, 29.90, 29.88, 29.85, 29.82, 29.81, 29.80, 29.76, 29.72, 29.66, 29.59, 29.53, 29.52, 29.42 (CH_2_^acyl^), 25.80 (CH_3_ *t*Bu), 25.52, 25.28, 25.24, 25.16, 25.14, 22.84 (CH_2_^acyl^), 17.99 (C_q_ *t*Bu), 14.26 (ω-CH_3_^acyl^), −3.83, −4.90 ppm (Si-CH_3_); HRMS (+ESI-TOF): *m*/*z* calcd for C_112_H_198_N_2_O_28_Si: 1023.6945 [*M*+2 H]^2+^; found: 1023.6978.

Compound **24**: *R*_f_=0.48 (toluene/ethyl acetate, 3:2); 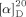
=+13 (*c*=0.3 in chloroform); ^1^H NMR (600 MHz, CDCl_3_, TMS, α/β=10:1, α-anomer): *δ*=6.03 (d, *J*=8.0 Hz, 1 H; N′H), 5.97–5.85 (m, 6 H; 5×=CH Alloc, NH), 5.40–5.23 (m, 11 H; 5×=CH_2_ Alloc, H-3), 5.18 (t, *J*_2,1_=*J*_1−OH,1_=3.3 Hz, 1 H; H-1), 5.12 (dd, *J*_4′,3′_=8.6 Hz, *J*_2′_,_3′_=10.6 Hz, 1 H; H-3′), 5.06–4.97 (m, 4 H; β-CH^acyl^), 4.96 (d, *J*_2′,1′_=8.2 Hz, 1 H; H-1′), 4.81 (m, 1 H; 1-OH), 4.67–4.55 (m, 11 H; 5×OCH_2_ Alloc, H-4), 4.49 (dd, *J*_5′,6a′_=2.0 Hz, *J*_6a′,6b′_=11.9 Hz, 1 H; H-6a′), 4.40 (dd, *J*_5′,6b′_=4.3 Hz, 1 H; H-6b′), 4.23–4.16 (m, 2 H; H-2, H-5), 3.80 (dd, *J*_6a,b_=12.2 Hz, *J*_5,6a_=1.5 Hz, 1 H; H-6a), 3.69 (dd, *J*_5,6b_=7.5 Hz, 1 H; H-6b), 3.64–3.51 (m, 3 H; H-4′, H-5′, H-2′), 3.09 (d, *J*_OH,4′_=4.1 Hz, 1 H; 4′-OH), 2.70–2.16 (m, 10 H; α-CH_2_^acyl^), 1.75–1.51 (m, 10 H; 4×γ-CH_2_^acyl^, β-CH_2_^acyl^), 1.47–1.11 (m, 100 H; CH_2_^acyl^), 0.88 ppm (t, *J*=7.0 Hz, 15 H; ω-CH_3_^acyl^); ^13^C NMR (151 MHz, CDCl_3_): *δ*=174.58, 171.04, 170.52, 170.49, 169.56 (CO, CONH), 155.36, 155.04, 154.64, 154.58, 154.34 (5×C_q_ Alloc), 131.87, 131.85, 131.52, 131.42, 131.39 (5×=CH Alloc), 119.51, 119.41, 119.33, 119.01, 118.82 (5×=CH_2_ Alloc), 100.62 (C-1′), 91.32 (C-1), 75.63, 75.57, 74.42 (β-CH^acyl^, C-3′), 73.99 (C-4′), 73.05 (C-4), 71.45, 71.42 (β-CH^acyl^, C-3), 69.60 (C-5), 69.20, 68.95, 68.91 (OCH_2_ Alloc), 68.84 (C-5′), 68.56, 68.52 (OCH_2_ Alloc), 67.91 (C-6), 66.41 (C-6′), 55.13 (C-2′), 52.48 (C-2), 41.93, 41.33, 39.84, 38.77 (4×α-CH_2_^acyl^), 34.69 (α-CH_2_^acyl^), 34.67, 34.48, 34.43, 34.17 (γ-CH_2_^acyl^, β-CH_2_^acyl^), 32.08, 29.84, 29.82, 29.79, 29.75, 29.72, 29.68, 29.65, 29.60, 29.53, 29.51, 29.47, 29.38, 25.43, 25.20, 25.18, 25.16, 25.06, 22.84 (CH_2_^acyl^), 14.26 ppm (ω-CH_3_^acyl^); HRMS (ESI) *m*/*z* calcd for C_106_H_184_N_2_O_28_ [*M*+2 H]^2+^: 966.6512; found: 966.6514.

**4-*O*-Allyloxycarbonyl-6-*O*-(6-*O*-allyloxycarbonyl-4-*O*-(bisallyloxy)-phosphoryl-3-*O*-[(*R*)-3-(allyloxycarbonyloxy)tetradecanoyl]-2-deoxy-2-[(*R*)-3-(tetradecanoyloxy)hexadecanoylamino]-β-D-glucopyranosyl)-1-*O*-(bis-allyloxy)phosphoryl-2-[(*R*)-3-(allyloxycarbonyloxy)-hexadecanoylamino]-3-*O*-[(*R*)-3-(allyloxycarbonyloxy)tetradecanoyl]-2-deoxy-α-D-glucopyranose (25)**: A solution of 1 *H*-tetrazole in dry acetonitrile (0.45 m, 336 μL, 0.15 mmol) was added to a stirred solution of **24** (39 mg, 0.02 mmol) and diallyl *N*,*N*-diisopropylphosphoramidite (40 μL, 0.15 mmol) in dry CH_2_Cl_2_ (3 mL) under Ar. The reaction mixture was stirred for 1 h at RT and PNO (46 mg, 0.15 mmol) was added under Ar at RT. The stirring was continued for 15 min. The mixture was then diluted with CH_2_Cl_2_ (100 mL), washed with satd. aq. Na_2_S_2_O_3_/sat. aq. NaHCO_3_ (1:1, 30 mL), sat. aq. NaHCO_3_ (20 mL) and water (20 mL). The organic phase was dried over Na_2_SO_4_, filtered and concentrated. The residue was purified by column chromatography on silica gel (toluene/ethyl acetate, 3:1 supplemented with 0.5 % NEt_3_), the residue was additionally purified by precipitation as follows. The residue was dissolved in CH_2_Cl_2_/acetone (1:1, 2 mL) and then petroleum ether (8 mL) was added. Then the volume was reduced to 5 mL by evaporation, the suspension was cooled to 0 °C and the solids were separated on the glass filter. The filtrate was concentrated to dryness and re-purified by precipitation. The combined precipitates were purified by chromatography on silica gel (toluene/ethyl acetate, 3:1 supplemented by 0.5 % NEt_3_) to give **25** (38 mg, 84 %) as a syrup. *R*_f_=0.30 (toluene/ethyl acetate, 2:1); 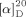
=+29 (*c*=0.6 in chloroform); ^1^H NMR (600 MHz, CDCl_3_, TMS): *δ*=6.73 (d, *J*=8.3 Hz, 1 H; N′H), 6.12 (d, *J*=8.2 Hz, 1 H; NH), 5.99–5.85 (m, 9 H; 5×=CH Alloc, 4×=CH Allyl), 5.69 (dd, *J*_2,1_=3.3 Hz, *J*_P,1_=5.3 Hz, 1 H; H-1), 5.44–5.21 (m, 20 H; 5×=CH_2_ Alloc, 4×=CH_2_ Allyl, H-3′, H-3), 5.21–5.15 (m, 1 H; β-CH^acyl^), 5.10–5.05 (m, 1 H; β-CH^acyl^), 5.02–4.96 (m, 2 H; 2×β-CH^acyl^), 4.93 (d, *J*_2′,1′_=8.3 Hz, 1 H; H-1′), 4.83 (dd, *J*_3,4_=9.6 Hz, *J*_5,4_=10.2 Hz, 1 H; H-4), 4.66–4.44 (m, 19 H; 5×–OCH_2_ Alloc, 4×OCH_2_ Allyl, H-6a′), 4.34–4.25 (m, 3 H; H-4′, H-6b′, H-2), 4.18 (ddd, *J*_5,6a_=1.5 Hz, *J*_5,6b_=5.4 Hz, 1 H; H-5), 3.86 (dd, *J*_6a,b_=12.3 Hz, 1 H; H-6a), 3.71 (dd, 1 H; H-6b), 3.68–3.61 (m, 2 H; H-5′, H-2′), 2.72 (dd, *J*_vic_=7.6 Hz, *J*_gem_=16.9 Hz, 1 H; α-CH_2a_^acyl^), 2.68 (dd, *J*_vic_=4.8 Hz, 1 H; α-CH_2b_^acyl^), 2.60–2.53 (m, 2 H; α-CH_2_^acyl^), 2.51–2.37 (m, 4 H; α-CH_2_^acyl^), 2.35–2.23 (m, 2 H; α-CH_2_^acyl^), 1.70–1.50 (m, 10 H; 4×γ-CH_2_^acyl^, β-CH_2_^acyl^), 1.37–1.20 (m, 100 H; CH_2_^acyl^), 0.88 ppm (t, *J*=7.0 Hz, 15 H; ω-CH_3_^acyl^); ^13^C NMR (151 MHz, CDCl_3_): *δ*=173.55, 170.71, 170.47, 169.84, 169.65 (CO, CONH), 154.79, 154.74, 154.68, 154.61, 154.06 (5×C_q_ Alloc), 132.60, 132.56, 132.48, 132.44, 132.29, 132.25, 131.85, 131.83, 131.73, 131.25 (=CH Allyl, Alloc), 119.66, 119.35, 119.19, 119.08, 118.97, 118.95, 118.73, 118.63 (=CH_2_ Alloc, Allyl), 99.76 (C-1′), 95.57 (d, *J*=6.5 Hz, C-1), 75.19, 74.63, 74.42 (3×β-CH^acyl^), 73.68 (d, *J*=6.5 Hz, C-4′), 73.01 (C-3′), 72.64 (d, *J*=5.4 Hz, C-5′), 71.92 (C-5), 71.65 (C-4), 70.58 (β-CH^acyl^), 70.32 (C-3), 69.43, 69.17, 69.14, 68.90, 68.86, 68.83, 68.77, 68.65, 68.59, 68.46 (OCH_2_ Alloc, Allyl), 66.38 (C-6), 65.99 (C-6′), 54.89 (C-2′), 52.24 (d, *J*=8.8 Hz, C-2), 40.99, 38.90, 38.65 (α-CH_2_^acyl^), 34.56, 34.34, 34.27, 34.21 (α-CH_2_^acyl^, γ-CH_2_^acyl^, β-CH_2_^acyl^), 32.09, 29.92, 29.89, 29.85, 29.82, 29.80, 29.75, 29.72, 29.68, 29.64, 29.61, 29.58, 29.53, 29.51, 29.44, 25.49, 25.25, 25.17, 25.12, 22.84 (CH_2_^acyl^), 14.26 ppm (ω-CH_3_^acyl^); ^31^P NMR (243 MHz, CDCl_3_): *δ*=0.37, −1.56 ppm.

**2-Deoxy-6-*O*-(2-deoxy-3-*O*-[(*R*)-3-hydroxytetradecanoyl]-4-*O*-phosphoryl-2-[(*R*)-3-(tetradecanoyloxy)hexadecanoylamino]-β-D-glucopyranosyl)-2-deoxy-2-[(*R*)-3-hydroxyhexadecanoylamino]-3-*O*-[(*R*)-3-hydroxytetradecanoyl]-α-D-glucopyranose 1,4′-bisphosphate (triethylammonium salt) (2)**: To a stirred solution of **25** (14.5 mg, 6.43 μmol) in dry CHCl_3_/MeOH (4:1, 4 mL) was added [CpRu^IV^(π-C_3_H_5_)(2-quinolinecarboxylato)]PF_6_ complex[63] (4 mg, 6.6 μmol) and the reaction was stirred for 2 h at RT. The crude reaction mixture was applied to a DEAE cellulose column (CH_3_COO^−^ form, 1×10 cm) equilibrated with CHCl_3_/MeOH/H_2_O (2:3:1, v/v/v). The column was washed with CHCl_3_/MeOH/H_2_O (2:3:1, 25 mL) and then developed with a stepwise gradient (30 mL each) of 2:3:1 CHCl_3_/MeOH/aq. CH_3_COO^−^HNEt_3_^+^ (0.06 m→0.08 m→0.12 m→0.2 m). Appropriate fractions were collected, the total volume was adjusted to 240 mL by addition of CHCl_3_/MeOH/H_2_O (2:3:1, v/v/v). The solution was transferred to an extraction funnel and converted to a two-phase Bligh–Dyer system by changing the solvent proportions to 2:2:1.8 by addition of CHCl_3_ (40 mL) and water (68 mL). The phases were resolved, the lower phase was concentrated, the residue was desalted by dissolution in CHCl_3_/MeOH/H_2_O (2:3:1, v/v/v, 180 mL) and rendering to a Bligh–Dyer mixture by addition of CHCl_3_ (40 mL), methanol (10 mL) and water (60 mL). The phases were resolved, and the lower phase was separated and concentrated. The residue was purified by gel permeation chromatography on Sephadex SX1 (toluene/CH_2_Cl_2_/MeOH, 2:2:1) to afford **2** (5.3 mg, 50 %) as a solid. *R*_f_=0.6 (chloroform/pyridine/formic acid/methanol/water, 50:50:14:2:5) or *R*_f_=0.45 (CHCl_3_/MeOH/H_2_O, 100:75:15 supplemented with 0.5 % of 33 % aq. NH_4_OH,); ^1^H NMR (600 MHz, CDCl_3_/MeOD, 4:1, TMS): *δ*=5.52 (dd, 1 H; *J*_1,2_=3.3 Hz, *J*_P,1_=6.6 Hz, H-1), 5.18 (t, 1 H; *J*_2′,3′_=*J*_3′,4′_=9.6 Hz, H-3′), 5.14 (t, 1 H; *J*_2,3_=*J*_3,4_=10.2 Hz, H-3), 5.09 (m, 1 H; β-CH^acyl^), 4.68 (d, *J*_2′,1′_=8.2 Hz, 1 H; H-1′), 4.28 (m, 1 H; H-4′), 4.19 (m, 1 H; H-2, under pre-saturated OH signal), 4.06 (m, 1 H; H-5), 4.04–3.94 (m, 3 H; H-6a, 2×β-CH^acyl^), 3.91–3.80 (m, 5 H; β-CH^acyl^, H-2′, H-6′a, H-6′b, H-6b), 3.49–3.42 (m, 2 H; H-4, H-5′), 3.13 (q, 4 H; CH_2_, Et_3_NH^+^-salt), 2.50–2.27 (m, 10 H; 5×α-CH_2_^acyl^), 1.73–1.39 (m, 10 H; 4×γ-CH_2_^acyl^, β-CH_2_^acyl^), 1.34 (t, 6 H; CH_3_, Et_3_NH^+^-salt), 1.28–1.23 (m, 100 H; -CH_2_^acyl^), 0.86 ppm (t, *J*=7.0 Hz, 15 H; ω-CH_3_^acyl^); negative MALDI-TOF: *m*/*z* calcd for C_86_H_163_NO_24_P_2_: 1670.108 [*M*−H]^−^; found: 1670.051; positive ESI-MS: *m*/*z* calcd for C_86_H_164_N_2_NaO_24_P_2_: 1694.12 [*M*+H]^+^; found: 1694.10; *m*/*z* calcd for C_86_H_164_N_2_Na_2_O_24_P_2_: 1716.06 [*M*+H]^+^; found: 1716.08.

***tert*****-Butyldimethylsilyl 4-*O*-allyloxycarbonyl-6-*O*-(6-*O*-allyloxycarbonyl-4-*O*-(bis-allyloxy)phosphoryl-3-*O*-[(*R*)-3-(allyloxycarbonyloxy)-tetradecanoyl]-2-deoxy-2-[(*R*)-3-(tetradecanoyloxy)hexadecanoyl-amino]-β-D-glucopyranosyl)-2-[(*R*)-3-(allyloxycarbonyloxy)hexadecanoylamino]-3-*O*-[(*R*)-3-(allyloxycarbonyloxy)tetradecanoyl]-2-deoxy-β-D-glucopyranoside (26)**: A solution of 1 *H*-tetrazole in dry acetonitrile (0.45 m, 500 μL, 0.22 mmol) was added to a stirred solution of **23** (272 mg, 0.14 mmol) and diallyl *N*,*N*-diisopropylphosphoramidite (60 μL, 0.22 mmol) in dry CH_2_Cl_2_ (5 mL) under Ar. The reaction mixture was stirred for 30 min at RT and then PNO (70 mg, 0.22 mmol) was added. The stirring was continued for 10 min., the mixture was diluted with CH_2_Cl_2_ (100 mL), washed with 1 n HCl (20 mL), sat. aq. Na_2_S_2_O_3_ (20 mL) and sat. aq. NaHCO_3_ (10 mL). The organic phase was dried over Na_2_SO_4_, filtered and concentrated. The residue was purified by column chromatography on silica gel (toluene/ethyl acetate, 4:1) to give **26** (272 mg, 93 %) as a syrup. *R*_f_=0.31 (toluene/ethyl acetate, 4:1); 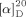
=+13 (*c*=0.89 in chloroform); ^1^H NMR (600 MHz, CDCl_3_, TMS): *δ*=6.08 (d, *J*=7.5 Hz, 1 H; N′H), 5.97–5.86 (m, 7 H; 5×=CH Alloc, 2×=CH Allyl), 5.85 (d, *J*=8.8 Hz, 1 H; NH), 5.53 (dd, *J*_4′,3′_=8.8 Hz, *J*_2′_,_3′_=10.4 Hz, 1 H; H-3′), 5.39–5.21 (m, 15 H; 5×=CH_2_ Alloc, 2×=CH_2_ Allyl, H-3), 5.18–5.12 (m, 1 H; β-CH^acyl^), 5.08–5.03 (m, 1 H; β-CH^acyl^),), 5.01–4.93 (m, 2 H; β-CH^acyl^), 4.99 (d, *J*_2′,1′_=8.2 Hz, 1 H; H-1′), 4.92 (d, *J*_2,1_=7.7 Hz, 1 H; H-1), 4.76 (t, *J*_3,4_=*J*_5,4_=9.5 Hz, 1 H; H-4), 4.68–4.57 (m, 10 H; 5×OCH_2_ Alloc), 4.53 (dd, *J*_6a′,b′_=11.6 Hz, *J*_5′,6a′_=2.0 Hz, 1 H; H-6a′), 4.53–4.45 (m, 4 H; 2×-OCH_2_ Allyl), 4.31 (t, *J*=9.2 Hz, 1 H; H-4′), 4.26 (dd, *J*_5′,6b′_=5.6 Hz, 1 H; H-6b′), 3.88 (dd, *J*_6a,b_=11.5 Hz, *J*_5,6a_=2.8 Hz, 1 H; H-6a), 3.75–3.68 (m, 3 H; H-2, H-5, H-5′), 3.64 (dd, *J*_5,6b_=5.4 Hz, 1 H; H-6b), 3.32 (ddd, 1 H; H-2′), 2.73 (dd, *J*=4.5 Hz, *J*=16.1 Hz, 1 H; α-CH_2a_^acyl^), 2.65 (dd, *J*=7.4 Hz, 1 H; α-CH_2b_^acyl^), 2.60–2.25 (m, 8 H; α-CH_2_^acyl^), 1.73–1.50 (m, 10 H; 4×γ-CH_2_^acyl^, β-CH_2_^acyl^), 1.39–1.10 (m, 100 H; CH_2_^acyl^), 0.88 (t, 15 H; 5×ω-CH_3_^acyl^), 0.87 (s, 9 H; *t*Bu), 0.10, 0.08 ppm (2 s, 6 H; 2×Si-Me); ^13^C NMR (150 MHz, CDCl_3_): *δ*=173.81, 170.34, 169.87, 169.55, 169.22 (CO, CONH), 154.75, 154.54, 154.51, 154.20 (C_q_ Alloc), 132.47, 132.38 (2 ×=CH Allyl), 131.87, 131.75, 131.74, 131.61, 131.54 (5×=CH Alloc), 119.50, 119.45, 119.12, 119.10, 118.89, 118.74 (=CH_2_ Alloc, =CH_2_ Allyl), 100.05 (C-1′), 95.84 (C-1), 75.46, 74.92, 74.57 (3×β-CH^acyl^), 73.81 (d, *J*=6.9 Hz, C-4′), 73.45 (C-4), 72.78 (C-3), 72.51 (C-5), 72.41 (d, *J*=5.7 Hz, C-5′), 72.26 (C-3′), 70.53 (β-CH^acyl^), 69.22, 68.89, 68.85, 68.81, 68.65, 68.56 (OCH_2_ Alloc, Allyl, C-6), 65.99 (C-6′), 57.03 (C-2), 56.22 (C-2′), 41.44, 41.21, 38.89, 38.63, 34.60 (5×α-CH_2_^acyl^), 34.49, 34.27, 34.12, 34.03 (γ-CH_2_^acyl^, β-CH_2_^acyl^), 32.08, 29.91, 29.88, 29.87, 29.84, 29.83, 29.78, 29.76, 29.73, 29.61, 29.58, 29.52, 29.44 (CH_2_^acyl^), 25.79 (CH_3_ *t*Bu), 25.42, 25.28, 25.19, 25.12, 22.84 (CH_2_^acyl^), 17.98 (C_q_ *t*Bu), 14.25 (ω-CH_3_^acyl^), −3.84, −4.94 ppm (Si-CH_3_); ^31^P NMR (243 MHz, CDCl_3_): *δ*=−1.65 ppm; HRMS (+ESI-TOF): *m*/*z* calcd for C_118_H_207_N_2_O_31_PSi: 1103.7089 [*M*+2 H]^2+^; found: 1103.7073.

**4-*O*-Allyloxycarbonyl-6-*O*-(6-*O*-allyloxycarbonyl-4-*O*-(bis-allyloxy)-phosphoryl-3-*O*-[(*R*)-3-(allyloxycarbonyloxy)tetradecanoyl]-2-deoxy-2-[(*R*)-3-(tetradecanoyloxy)hexadecanoylamino]-β-D-glucopyranosyl)-2-[(*R*)-3-(allyloxycarbonyloxy)hexadecanoylamino]-3-*O*-[(*R*)-3-(allyloxycarbonyloxy)tetradecanoyl]-2-deoxy-D-glucopyranose (27)**: Triethylamine hydrofluoride (TREAT, 263 μL, 1.61 mmol) was added to a solution of **26** (145 mg, 0.07 mmol) in dry THF (3 mL) in a PTFE flask, and the reaction was stirred under Ar at RT for 48 h. Then the mixture was diluted with ethyl acetate (200 mL) and washed sat. NaHCO_3_ (50 mL), 1 m HCl (20 mL) and sat. aq. NaHCO_3_ (50 mL). The organic phase was dried over Na_2_SO_4_, filtered and concentrated. The residue was purified by MPLC (toluene/ethyl acetate 3:1→1:1) to afford **27** (120 mg, 87 %) as a syrup. *R*_f_=0.45 (toluene/ethyl acetate 1:1); 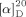
=+21 (*c*=0.66 in chloroform); ^1^H NMR (600 MHz, CDCl_3_, TMS): α/β=10:1, for α-anomer: *δ*)=6.20 (d, *J*=7.3 Hz, 1 H; N′H), 5.98–5.84 (m, 8 H; 5×=CH Alloc, 2×=CH Allyl, NH), 5.48 (dd, *J*_4′,3′_=8.6 Hz, *J*_2′,3′_=10.7 Hz, 1 H; H-3′), 5.45 (d, *J*_2′,1′_=8.2 Hz, 1 H; H-1′), 5.39–5.21 (m, 15 H; 5×=CH_2_ Alloc, 2×=CH_2_ Allyl, H-3), 5.19 (at, *J*_2,1_ ∼*J*_OH,1_=3.1 Hz, 1 H; H-1), 5.14–4.96 (m, 4 H; β-CH^acyl^), 4.96–4.94 (m, 1 H; -OH), 4.66–4.55 (m, 11 H; 5×-OCH_2_ Alloc, H-4), 4.55–4.44 (m, 5 H; 2×-OCH_2_ Allyl, H-6a′), 4.36 (t, *J*=9.2 Hz, 1 H; H-4′), 4.31 (dd, *J*_5′,6b′_=4.7 Hz, *J*_6a′,6b′_=12.1 Hz, 1 H; H-6b′), 4.23–4.18 (m, 1 H; H-2), 4.18–4.14 (m, 1 H; H-5), 3.78 (dd, *J*_6a,b_=12.8 Hz, *J*_5,6a_=7.6 Hz, 1 H; H-6a), 3.74–3.68 (m, 2 H; H-6b, H-5′), 3.23 (ddd, 1 H; H-2′), 2.76 (dd, *J*=4.6 Hz, *J*=15.8 Hz, 1 H; α-CH_2a_^acyl^), 2.62 (dd, *J*=7.1 Hz, 1 H; α-CH_2b_^acyl^), 2.59 (dd, *J*=7.7 Hz, *J*=16.3 Hz, 1 H; α-CH_2a_^acyl^), 2.53 (dd, *J*=5.1 Hz, 1 H; α-CH_2b_^acyl^), 2.50–2.26 (m, 6 H; α-CH_2_^acyl^), 1.74–1.52 (m, 10 H; 4×γ-CH_2_^acyl^, β-CH_2_^acyl^), 1.44–1.11 (m, 100 H; CH_2_^acyl^), 0.88 (t, *J*=7.0 Hz, 15 H; ω-CH_3_^acyl^); ^13^C NMR (150 MHz, CDCl_3_): *δ*=174.37, 171.02, 170.48, 169.59, 169.53 (CO, CONH), 154.87, 154.63, 154.60, 154.50, 154.28 (5×C_q_ Alloc), 132.43, 132.38, 132.33, 131.90, 131.88, 131.60, 131.53, 131.41 (=CH Allyl, Alloc), 119.68, 119.38, 119.19, 118.94, 118.80, 118.74 (=CH_2_ Alloc, Allyl), 99.28 (C-1′), 91.36 (C-1), 75.56, 75.07, 74.42 (3×β-CH^acyl^), 73.60 (d, *J*=5.5 Hz, C-4′), 73.02 (C-4), 72.66 (d, *J*=6.1 Hz, C-5′), 72.12 (C-3′), 71.57 (C-3), 70.81 (β-CH^acyl^), 70.23 (C-5), 69.18, 68.88, 68.85, 68.79, 68.54, 68.51 (OCH_2_ Alloc, Allyl), 67.39 (C-6), 65.70 (C-6′), 56.49 (C-2′), 52.36 (C-2), 41.68, 41.29, 39.03, 38.75, 34.59 (5×α-CH_2_^acyl^), 34.51, 34.46, 34.16, 34.07 (γ-CH_2_^acyl^, β-CH_2_^acyl^), 32.08, 29.88, 29.86, 29.85, 29.83, 29.77, 29.71, 29.69, 29.61, 29.60, 29.58, 29.52, 25.40, 25.42, 25.33, 25.22, 25.14, 25.06, 22.84 (CH_2_^acyl^), 14.26 ppm (ω-CH_3_^acyl^); ^31^P NMR (243 MHz, CDCl_3_): *δ*=−1.75 ppm; HRMS (+ESI-TOF): *m*/*z* calcd for C_112_H_195_N_3_O_31_P: 2109.3507 [*M*+NH_4_]^+^; found: 2109.3538.

**2,3-Di-*O*-allyloxycarbonyl-4-azido-4-deoxy-β-l-arabinopyranosyl-1-*O*-phosphoryl 4-*O*-allyloxycarbonyl-6-*O*-(6-*O*-allyloxycarbonyl-4-*O*-(bis-allyloxy)phosphoryl-3-*O*-[(*R*)-3-(allyloxycarbonyloxy)tetradecanoyl]-2-deoxy-2-[(*R*)-3-(tetradecanoyloxy)hexadecanoylamino]-β-D-glucopyranosyl)-2-[(*R*)-3-(allyloxycarbonyloxy)hexadecanoylamino]-3-*O*-[(*R*)-3-(allyloxycarbonyloxy)tetradecanoyl]-2-deoxy-α-D-glucopyranose triethylammonium salt (28)**: A solution of PyNTP (196 mg, 0.393 mmol) in CH_2_Cl_2_ (0.5 mL) and a solution of 2,6-lutidine (130 μL, 1.12 mmol) in CH_2_Cl_2_ (0.5 mL) were added successively to a stirred solution of **27** (α/β 10:1; 25 mg, 0.012 mmol) and H-phosphonate **6** (47.6 mg, 0.112 mmol) in dry CH_3_CN/CH_2_Cl_2_ (4:1, 3 mL) and the reaction was stirred for 5 h at RT under Ar. *N*,*O*-Bis(trimethylsilyl)acetamide (137 μL, 0.56 mmol) followed by triethylamine (78 μL, 0.73 mmol) were added under Ar and the stirring was continued for 15 min. Then, (1*S*)-(+)-(10-camphorsulfonyl)oxaziridine (CSO) (232 mg, 1.01 mmol) was added and the reaction mixture was stirred for 15 min at RT under Ar. The mixture was diluted with chloroform (30 mL) and washed with a mixture of a 7 % Na_2_S_2_O_3_ solution/1 m TEAB buffer (1:1, 10 mL). The aqueous phase was re-extracted with chloroform (2×50 mL), the combined organic phases were dried by filtration over cotton, diluted with toluene (10 mL) and concentrated. The residue was purified by chromatography on silica gel (ethyl acetate/acetone, 3:1 supplemented with 0.5 % triethylamine) to give **28** (19 mg, 61 %) as a transparent solid. *R*_f_=0.36 (ethyl acetate/acetone 2:1); 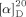
=+43 (*c*=0.73 in chloroform); ^1^H NMR (600 MHz, CDCl_3_/MeOD, 4:1, TMS): *δ*=5.89–5.79 (m, 9 H; 7×=CH Alloc, 2×=CH Allyl), 5.82 (dd, *J*_2,1_=3.5 Hz, *J*_P,1_=7.4 Hz, 1 H; H-1′′ Ara4N), 5.53 (dd, *J*_2,1_=3.3 Hz, *J*_P,1_=7.1 Hz, 1 H; H-1), 5.39–5.32 (m, 9 H; 2×=CH_2_ Alloc, 2×=CH_2_ Allyl, H-3′), 5.30–5.24 (m, 12 H; 5×=CH_2_ Alloc, H-3, H-3′′Ara4N), 5.20 (m, 1 H; β-CH^acyl^), 5.15 (m, 1 H; H-2′′Ara4N), 5.10 (m, 1 H; β-CH^acyl^), 5.03–4.98 (m, 2 H; 2×β-CH^acyl^), 4.80–4.88 (d, 1 H; *J*_2′,1′_=10.3 Hz, H-1′), 4.86 (t, *J*_3,4_=*J*_4,5_=8.3 Hz, 1 H; H-4), 4.71–4.48 (m, 19 H; 7×OCH_2_ Alloc, 2×OCH_2_ Allyl, H-6′a), 4.25 (t, *J*=9.2 Hz, 1 H; H-4′), 4.23–4.15 (m, 4 H; H-6′b, H-4′′Ara4N, H-5a′′ Ara4N, H-2), 4.15–4.10 (m, 1 H; H-5 under water signal), 3.94 (dd, *J*_5a,b_=12.9 Hz, *J*_4,5a_=1.8 Hz, 1 H; H-5a′′ Ara4N), 3.90 (dd, *J*_6a,b_=12.1 Hz, *J*_5,6a_=2.0 Hz, 1 H; H-6a), 3.67 (ddd, *J*_4′,5′_=9.8 Hz, *J*_6a′,5′_=1.5 Hz, *J*_6b′,5′_=5.0 Hz, 1 H; H-5′), 3.65 (dd, *J*_1′,2′_=8.5 Hz, *J*_3′,2′_=10.5 Hz, 1 H; H-2′), 3.56 (dd, *J*_5,6b_=4.2 Hz, 1 H; H-6b), 5.74 (q, *J*=9.2 Hz, 6 H; CH_2_ NEt_3_), 2.66 (dd, *J*=7.6 Hz, *J*=17.3 Hz, 1 H; α-CH_2a_^acyl^), 2.62 (dd, *J*=4.8 Hz, 1 H; α-CH_2b_^acyl^), 2.52–2.09 (m, 8 H; α-CH_2_^acyl^), 1.62–1.44 (m, 10 H; 4×γ-CH_2_^acyl^, β-CH_2_^acyl^), 1.36–1.04 (m, 109 H; CH_2_^acyl^, CH_3_ NEt_3_), 0.80 ppm (t, *J*=7.0 Hz, 15 H; ω-CH_3_^acyl^); ^13^C NMR (150 MHz, CDCl_3_/MeOD 4:1): *δ*=173.74, 170.97, 170.16, 169.96, 169.82 (CO, CONH), 154.55, 154.45, 154.40, 154.00, 153.83, 153.74 (C_q_ Alloc), 131.64, 131.53, 131.48, 131.29, 131.19, 130.93 (=CH Allyl, Alloc), 118.91, 118.86, 118.82, 118.70, 118.63, 118.44, 118.39, 118.26 (=CH_2_ Alloc, Allyl), 99.44 (C-1′), 94.19 (d, *J*=5.8 Hz, C-1 GlcN), 93.02 (d, *J*=6.8 Hz, C-1′′), 75.18, 74.28, 74.13 (3×β-CH^acyl^), 73.63 (d, *J*=6.4 Hz, C-4′), 72.66 (C-3′), 72.44 (C-3′′), 72.00 (d, *J*=5.3 Hz, C-5′), 71.81 (C-4), 71.22, 71.17, 71.12 (C-3, C-2′′Ara4N), 70.43 (β-CH^acyl^), 69.97 (C-5), 68.98, 68.91, 68.86, 68.83, 68.75, 68.71, 68.53, 68.13 (OCH_2_ Alloc, Allyl), 66.28 (C-6), 65.59 (C-6′), 61.02 (C-5′′), 59.39 (C-4′′), 54.41 (C-2′), 51.32 (d, *J*=7.5 Hz, C-2), 45.95 (CH_2_ NEt_3_), 40.62, 39.95, 38.19, 38.07 (α-CH_2_^acyl^), 34.25, 34.13, 33.91, 33.86, 33.81, (α-CH_2_^acyl^, γ-CH_2_^acyl^, β-CH_2_^acyl^), 31.73, 29.59, 29.54, 29.51, 29.48, 29.46, 29.43, 29.38, 29.36, 29.35, 29.30, 29.17, 29.08, 25.20, 24.94, 24.88, 24.81, 24.69, 22.46 (CH_2_^acyl^), 13.73 (ω-CH_3_^acyl^), 8.28 ppm (CH_3_ NEt_3_); ^31^P NMR (243 MHz, CDCl_3_): *δ*=1.97, −0.48 ppm; HRMS (+ESI-TOF): *m*/*z* calcd for C_125_H_207_N_5_O_41_P_2_: 1249.1944 [*M*+2 H]^2+^; found: 1249.1943.

**4-Amino-β-l-arabinopyranosyl-1-*O*-phosphoryl 2-deoxy-6-*O*-(2-deoxy-3-*O*-[(*R*)-3-hydroxytetradecanoyl]-4-*O*-phosphoryl-2-[(*R*)-3-(tetradecanoyloxy)hexadecanoylamino]-β-D-glucopyranosyl)-2-deoxy-2-[(*R*)-3-hydroxyhexadecanoylamino]-3-*O*-[(*R*)-3-hydroxy-tetradecanoyl]-α-D-glucopyranose (1)**: The complex [CpRu^IV^(π-C_3_H_5_)(2-quinolinecarboxylato)]PF_6_ (2 mg, 3.3 μmol) was added to a stirred solution of **28** (6 mg, 2.3 μmol) in dry CHCl_3_/MeOH (4:1, 2 mL) and the reaction was stirred for 2 h at RT. The reaction mixture was directly applied onto a DEAE cellulose column (HCOO^−^ form, 1×5 cm). The column was washed with CHCl_3_/MeOH (4:1, 20 mL) and then developed with CHCl_3_/MeOH/0.2 m methanolic CH_3_COO^−^HNEt_3_^+^, 2:3:1). Appropriate fractions were collected and the total volume was adjusted to 240 mL by addition of 2:3:1 CHCl_3_/MeOH/H_2_O. The solution was transferred to an extraction funnel and converted to a two-phase Bligh–Dyer system by changing the solvent proportions to 2:2:1.8 by addition of CHCl_3_ (40 mL) and water (68 mL). The phases were resolved, the lower phase was filtered over cotton, diluted with toluene (10 mL) and concentrated to give an intermediate azide 4-azido-4-deoxy-β-l-arabinopyranosyl-1-*O*-phosphoryl 2-deoxy-6-*O*-(2-deoxy-3-*O*-[(*R*)-3-hydroxytetradecanoyl]-4-*O*-phosphoryl-2-[(*R*)-3-(tetradecanoyloxy)hexadecanoyl-amino]-β-D-glucopyranosyl)-2-deoxy-2-[(*R*)-3-hydroxyhexadecanoylamino]-3-*O*-[(*R*)-3-hydroxytetradeca-noyl]-α-D-glucopyranose (5 mg, 99 %) as a syrup; *R*_f_=0.60 (chloroform/methanol/water 100:75:15); (negative MALDI-TOF: *m*/*z* calcd for C_91_H_170_N_5_O_27_P_2_: 1827.156 [*M*−H]^−^; found: 1827.230). PtO_2_ (2 mg, 9 μmol) was added to a stirred solution of the intermediate azide (5.0 mg, 2.6 μmol) in dry toluene/MeOH (4:1, 1.2 mL). The atmosphere was exchanged to hydrogen and the reaction was stirred for 2 h at RT under H_2_. The solids were removed by filtration over a syringe filter (0.45 μm, regenerated cellulose) and washed with toluene/MeOH (4:1, 10 mL). The filtrate was concentrated, the residue was purified by gel permeation chromatography on Sephadex LH-20 (1×50 cm; elution with toluene/MeOH, 1:1). Yield: 3.4 mg (70 %) as a light-pink coloured solid (a mixture of several salt forms at phosphate: triethylammonium (white) and ruthenium (pink) salts). Alternatively, the residue (originating from the same amount of the azide: 5 mg) was purified by anion-exchange chromatography on a DEAE cellulose column (HCOO^−^-form, 10×1 cm) as follows. The column was equilibrated with 2:3:1 (v/v/v) CHCl_3_/MeOH/H_2_O. The residue was dissolved in CHCl_3_/MeOH (3:1, 2 mL) and let slowly absorbed onto a resin pad. The column was washed with CHCl_3_/MeOH/H_2_O (2:3:1, 25 mL) and then developed with the stepwise gradient (30 mL each) of 2:3:1 CHCl_3_/MeOH/aq. CH_3_COO^−^HNEt_3_^+^ (0.04 m→0.06 m→0.8 m→0.12 m). Appropriate fractions were collected, the total volume was adjusted to 240 mL by addition of CHCl_3_/MeOH/H_2_O (2:3:1, v/v/v). The solution was transferred to an extraction funnel and converted to a two-phase Bligh–Dyer system by changing the solvent proportions to 2:2:1.8 by addition of CHCl_3_ (40 mL) and water (68 mL). The phases were resolved, the lower phase was concentrated, the residue was desalted by a dissolution in CHCl_3_/MeOH/H_2_O (2:3:1, v/v/v, 180 mL) and rendering into a Bligh–Dyer mixture by addition of CHCl_3_ (40 mL), methanol (10 mL) and water (60 mL). The phases were resolved in the extraction funnel, and the lower phase was separated and concentrated. The residue was purified by chromatography on Sephadex SX1 (1×60 cm, toluene/CH_2_Cl_2_/MeOH, 2:2:1) to afford 1 (triethylammonium salt, 2.8 mg, 57 %) as a white solid. *R*_f_=0.35 (chloroform/pyridine/formic acid/methanol/water, 50:50:14:2:5) or *R*_f_=0.65 (CHCl_3_/MeOH/H_2_O, 100:75:15, supplemented with 0.5 % of 33 % aq. NH_4_OH,); ^1^H NMR (600 MHz, CDCl_3_-MeOD, 4:1): *δ*=5.56 (m, 1 H; H-1′′), 5.51 (m, 1 H; H-1), 5.10–5.25 (m, 3 H; H-3′, H-3), β-CH^acyl^), 4.77 (d, *J*_2′,1′_=8.0 Hz, 1 H; H-1′), 4.34 (m, 1 H; H-4′), 4.25 (m, 1 H; H-2, under pre-saturated OH signal), 4.15–3.80 (m, 9 H; H-5′′a, H-5, H-2′′, H6a, H-2′, H-6′a, 3×β-CH^acyl^), 3.75–3.50 (m, 4 H; H-5′′b, H-4′′, H-6b), 3.60–3.50 (m, 2 H; H-4, H-5′), 3.15 (q, CH_2_, Et_3_NH^+^-salt), 2.50–1.90 (m, 5×α-CH_2_^acyl^), 1.68–1.50 (m, 4×γ-CH_2_^acyl^, β-CH_2_^acyl^, CH_3_, Et_3_NH^+^-salt), 1.10–1.40 (m, CH_2_^acyl^), 0.83 ppm (t, 5×ω-CH_3_^acyl^); positive ESI-MS: *m*/*z* calcd for C_91_H_174_N_3_O_27_P_2_: 1803.18 [*M*+H]^+^; found: 1803.15; *m*/*z* calcd for C_91_H_173_N_3_NaO_27_P_2_: 1825.16 [*M*+H]^+^; found: 1825.14; *m*/*z* calcd for C_91_H_172_N_3_Na_2_O_27_P_2_: 1874.14 [*M*+H]^+^; found: 1874.12.

### Biological assays

**Reagents and cell cultures**: HEK293 stably expressing human TLR4, MD-2, CD14 and a secreted NF-κB dependent reporter (HEK-Blue hTLR4), *E. coli* O111:B4 LPS, *E. coli* serotype R515 *Re*-LPS were purchased from InvivoGen. The THP-1 cell line was obtained from Dr. Rene Devos (Roche Research Ghent) and originally purchased from ATCC. 12-*O*-Tetradecanoylphorbol-13-acetate (TPA) was purchased from Sigma. Lipid A **1** and **2** were reconstituted in DMSO/chloroform (2:1, v/v) to provide 1 mg mL^−1^ stock solutions. Further dilutions were made with cell medium (RPMI or DMEM) supplemented with 10 % FCS so that the final amount of DMSO/chloroform in the cell culture did not exceed 0.01 %.

**hTLR4/MD-2/CD14-Transfected HEK293 cells (Hek-Blue) activation assay**: Growth conditions and activation assay were set as recommended by InvivoGen. The cells were stimulated with the solutions of compounds **1** and **2** or Re*-*LPS in DMEM supplemented by 10 % FCS at the indicated concentrations. The compounds were added in a total volume of 20 μL to 25 000 HEK-Blue hTLR4 cells in 180 μL plates and were incubated for 20–24 h at 37 °C and 5 % CO_2_. SEAP levels were determined by incubation of 20 μL of challenged cells supernatants with 180 μL detection reagent (QUANTI-Blue) and the colour development was measured at 650 nm using a spectrophotometer (SpectraMAX 190). Data were combined from *n*=3 independent experiments, error bars indicate standard error of the mean.

**Assay in human macrophage-like cell line THP1**: THP-1 cells were grown in RPMI-1640 cell-culture medium (Life Technologies) that was supplemented with 2 mm l glutamine, 100 U mL^−1^ penicillin, 100 μg mL^−1^ streptomycin, and 10 % FCS. Cells were seeded in a 96 well plate at 10E5 cells/well in 150 μL complete medium and stimulated by 200 nm TPA for 24 h to induce the differentiation into macrophage-like cells. On the next day the cells were washed twice with complete culture medium to discard the cells that did not adhere, refreshed with 200 μL complete medium and left for 1 h to recover. Cells were stimulated with compounds **1** and **2** at the indicated concentration and with *E. coli* O111:B4 LPS, which were added as solutions in 10 μL complete medium. The total volume of the well after stimulation reached 220 μL. The cells were incubated for 18 h and the supernatants were analysed for TNF-α, IL-8 and MCP-1 by ELISA (BD Biosciences). Data are representative for two independent experiments. Error bars indicate standard error of the mean of duplicate samples.

## Supporting Information

As a service to our authors and readers, this journal provides supporting information supplied by the authors. Such materials are peer reviewed and may be re-organized for online delivery, but are not copy-edited or typeset. Technical support issues arising from supporting information (other than missing files) should be addressed to the authors.

miscellaneous_information
