# Supplementary material for: Chemical Synthesis of Burkholderia Lipid A Modified with Glycosyl Phosphodiester-Linked 4-Amino-4-deoxy-β-l-arabinose and Its Immunomodulatory Potential
Source: Chemistry. 2015 Jan 28;21(10):4102–14. doi: 10.1002/chem.201406058 (PMC4517147; doi:10.1002/chem.201406058)

CHEMISTRY
A European Journal

Supporting Information

© Copyright Wiley-VCH Verlag GmbH & Co. KGaA, 69451 Weinheim, 2015

Chemical Synthesis of *Burkholderia* Lipid A Modified with Glycosyl Phosphodiester-Linked 4-Amino-4-deoxy-β-L-arabinose and Its Immunomodulatory Potential

Ralph Hollaus,[a] Simon Ittig,[b] Andreas Hofinger,[a] Mira Haegman,[c] Rudi Beyaert,[c]
Paul Kosma,[a] and Alla Zamyatina*[a]

chem_201406058_sm_miscellaneous_information.pdf

Supplementary Information

Content

Supplementary SI-Figure 1 2
Supplementary SI-Figure 2 3
Supplementary SI-Figure 3 4
Experimental Procedures: Synthesis 5
Synthesis of 4 5
Synthesis of 5 5
Synthesis of 7 6
Synthesis of 8 6
Synthesis of fatty acids 9-11 6
Synthesis of 12 8
Synthesis of 13 and 14 8
Synthesis of 15 9
Synthesis of 16 10
Synthesis of 17 10
Synthesis of 18 11
Synthesis of 19 11
Synthesis of 20 12
NMR and Mass- spectra 12
^{1}H and ^{13}C NMR spectra; ESI-MS spectra of compounds 1 and 2 12

Supplementary SI-Figure 1

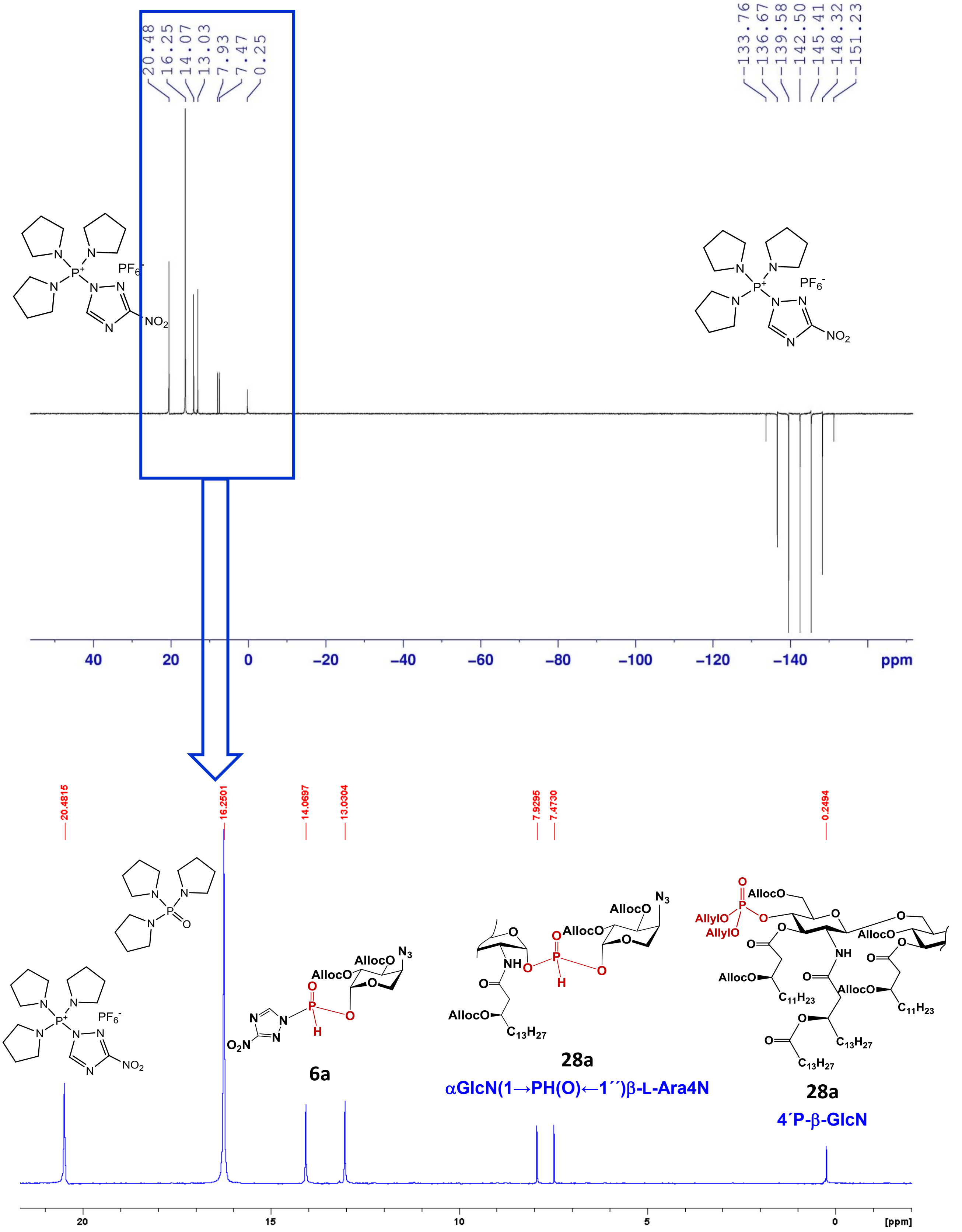

SI-Figure 1. Progress of the reaction **28** → **28a** (reaction solvent + CD_3CN) followed by ^{31}P NMR (161 MHz)

Supplementary SI-Figure 2

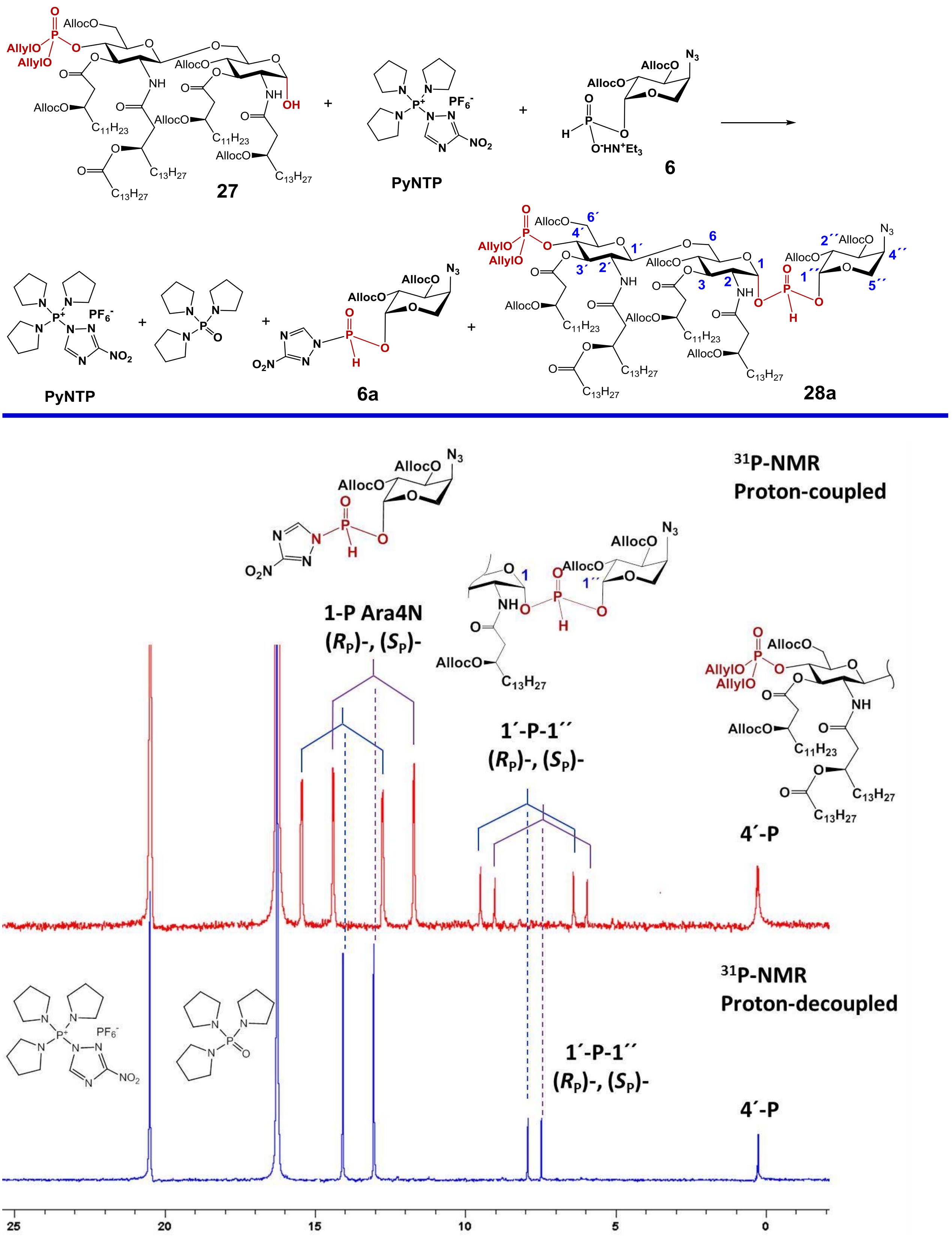

SI-Figure 2. Proton-decoupled and proton-coupled ^{31}P NMR spectra (CD_3CN, 161 MHz) of reaction mixture **28a**

Supplementary SI-Figure 3

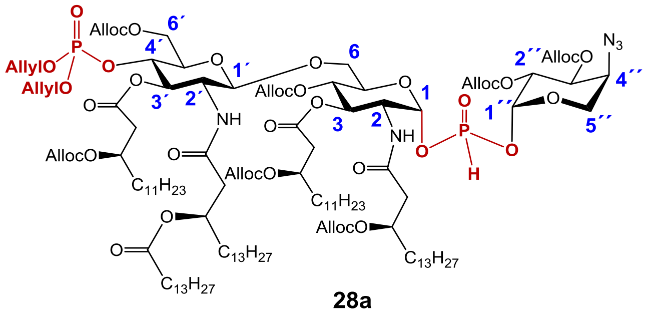

28a

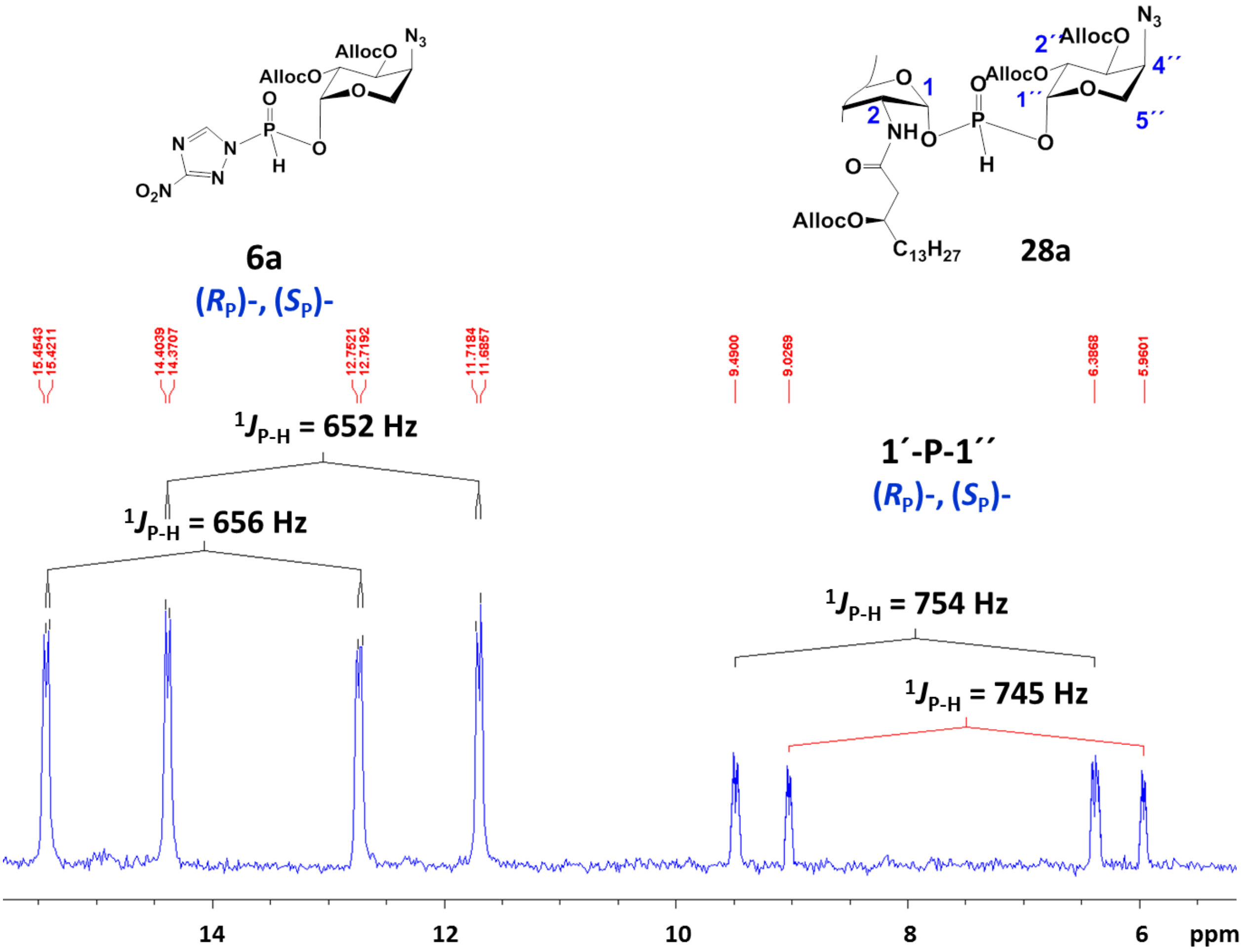

SI-Figure 3. ^{31}P NMR spectra (161 MHz, CD_3CN, proton-coupled) of H-phosphonate diester **28a** and activated Ara4N H-phosphonate **6a.**

Experimental Procedures: Synthesis

Synthesis of 4

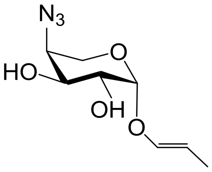

Propen-1-yl 4-azido-4-deoxy-β-L-arabinopyranoside (4)

A solution of allyl 4-azido-4-deoxy-β-L-arabinopyranoside **3** (717 mg, 3.33 mmol) in dry THF (20 ml) was degassed by repeated evacuation and flushing the flask with Ar. To this stirred solution H_2-activated $[Ir^+(COD)(PMePh_2)_2]PF_6^-$ [(1,5-cyclooctadiene)bis(methyldiphenylphosphine)iridium(I) hexa-fluorophosphate] (15 mg, 17.7 µmol) in THF (30 mL) was added and the reaction mixture was stirred for 1 h. [Activation of the catalyst was performed as follows: $Ir[(COD)_2bis(methyldiphenylphosphine)]PF_6^-$ (15 mg) was placed in a three necked flask and dissolved in dry THF (30 ml). The solution was first degassed by repeated evacuation and filling the flask with Ar, then Ar was exchanged to H_2, which was kept for 3×15 sec in the flask such that the solution turned from pink to colourless. The gaseous phase was exchanged to Ar and a solution of the activated catalyst was transferred to the reaction mixture.] The reaction mixture was stirred for 30 min at r.t. and concentrated. The residue was purified by column chromatography on silica gel (dichloromethane/acetone, 5:1) to give **4** as a syrup (710 mg, 99%). R_f = 0.25 (dichloromethane/acetone 5:1); $[\alpha]_D^{20}$ = +162 (*c* = 1.25 in methanol); ^{1}H NMR (600 MHz, MeOD, E/Z 14:1): *δ* = 6.21 (qd, J_{trans} = 12.2 Hz, J_{allyl} = 1.6 Hz, 1 H, -OCH= propenyl), 5.16 - 5.09 (m, 1 H, =CH propenyl), 5.00 (d, $J_{2,1}$ = 3.6 Hz, 1 H, H-1), 4.02 (d, $J_{4,3}$ = 4.0 Hz, $J_{2,3}$ = 9.8 Hz, 1 H, H-3), 3.89 - 3.86 (m, 1 H, H-4), 3.83 (dd, $J_{4,5a}$ = 1.7 Hz, $J_{5a,b}$ = 12.5 Hz, 1 H, H-5a), 3.74 (dd, 1 H, H-2), 3.60 (dd, $J_{4,5b}$ = 2.2 Hz, 1 H, H-5b), 1.55 (dd, J_{vic} = 6.9 Hz, 3 H, $-CH_3$ propenyl); ^{13}C NMR (150 MHz, MeOD): *δ* = 144.83 (-OCH= propenyl), 105.23 (=CH propenyl), 100.27 (C-1), 70.77 (C-3), 70.07 (C-2), 63.94 (C-4), 62.07 (C-5), 12.46 ($-CH_3$ propenyl); HRMS ($^+$ESI-TOF): calcd for $C_8H_{17}N_4O_4$ *m/z* $[M + NH_4]^+$: 233.1244; found 233.1247.

Synthesis of 5

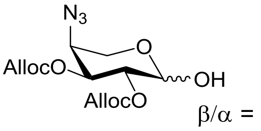

β/α = 2:1

2,3-di-*O*-Allyloxycarbonyl-4-azido-4-deoxy-α/β-L-arabinopyranose (5)

A solution of **4** (624 mg, 2.90 mmol) and TMEDA (770 µL, 5.16 mmol) in dry dichloromethane (20 mL) was cooled to 0°C and allylchloroformate (990 µL, 9.3 mmol) was added. The reaction mixture was stirred for 1 h at r.t., diluted with ethyl acetate (250 mL) and washed with 1 N aq. HCl (50 mL) followed by satd. aq. $NaHCO_3$ (50 mL). The organic phase was dried over Na_2SO_4, filtered and concentrated. The crude product was dissolved in THF (40 mL) and a solution of iodine in THF/H_2O (2:1, 12 mL) was added dropwise at 0°C. After stirring for 2.5 h at 0°C the reaction was quenched by addition of a 7% aq. $Na_2S_2O_3$ solution (50 mL). The reaction mixture was diluted with ethyl acetate (150 mL), the organic phase was washed with satd. aq. $NaHCO_3$ (50 mL), dried over Na_2SO_4, filtered and concentrated. The residue was purified by column chromatography on silica gel (dichloromethane/EtOAc, 12:1) to give **5** (745 mg, 75%, β/α = 2:1) as a syrup. R_f = 0.10 (hexane/EtOAc, 2:1); ^{1}H NMR (400 MHz, $CDCl_3$, TMS, β/α = 2:1): *δ* = 5.98 - 5.86 (m, 4 H, =CH Alloc), 5.50 (d, $J_{2\beta,1\beta}$ = 3.4 Hz, 1 H, H-1β), 5.40 – 5.24 (m, 8 H, $=CH_2$ Alloc), 5.27 (dd, $J_{2\beta,3\beta}$ = 10.4 Hz, $J_{4\beta,3\beta}$ = 3.7 Hz, 1 H, H-3β), 5.05 (dd, 1 H, H-2β), 4.97 - 4.90 (m, 2 H, H-2α, H-3α), 4.70 - 4.63 (m, 8 H, OCH_2 Alloc), 4.67 (m, 1 H, H-1α), 4.20 (ddd, $J_{5a\beta,4\beta}$ = 2.1 Hz, $J_{5b\beta,4\beta}$ = 2.9 Hz, 1 H, H-4β), 4.19 (dd, $J_{5a,b\beta}$ = 12.9 Hz, 1 H, H-5aβ), 4.12 (dd, $J_{3,4\alpha}$ = $J_{5a\alpha,4\alpha}$ = 3.3 Hz, $J_{5b\alpha,4\alpha}$ = 1.9 Hz, 1 H, H-4α), 4.08 (dd, $J_{5a,b\alpha}$ = 12.8 Hz, 1 H, H-5aα), 3.75 (dd, 1 H, H-5bβ), 3.67 (dd, 1 H, H-5bα); ^{13}C NMR (100 MHz, $CDCl_3$): *δ* = 154.69, 153.98, 153.90, 153.85 (C=O Alloc), 131.07, 131.00, 130.91, 130.89 (=CH Alloc), 119.43, 119.37, 119.32 ($=CH_2$ Alloc), 95.61 (C-1α), 90.78 (C-1β), 74.88 (C-3α), 74.31 (C-2α), 72.34 (C-3β), 71.74 (C-2β), 69.30, 69.27, 69.11, 69.10 (OCH_2 Alloc), 63.04 (C-5α), 60.04 (C-5β), 59.27 (C-4β), 58.15 (C-4α); HRMS ($^+$ESI-TOF): calcd for $C_{13}H_{17}N_3NaO_8$ *m/z* $[M + Na]^+$: 366.0908, found 366.0908

Synthesis of 7

Compound **7** was prepared as described[1] in 5 steps, one crystallization, 62 % yield as follows:

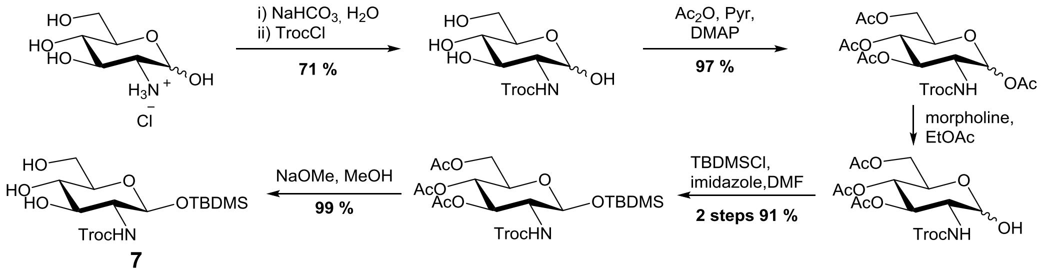

Analytical data of compound **7** were identical to those reported in the literature.[2]

Synthesis of 8

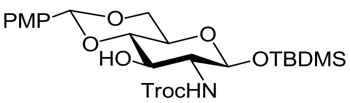

***Tert*-Butyldimethylsilyl 2-deoxy-4,6-*O*-(*p*-methoxybenzylidene)-2-(2,2,2-trichloroethoxycarbonylamino)-β-D-glucopyranoside (8)**

To a stirred solution of **7** (3 g, 6.40 mmol) in dry acetonitrile (100 mL), camphorsulfonic acid (149 mg, 0.64 mmol) and anisaldehyde dimethyl acetal (1.58 mL, 9.28 mmol) were added successively. The reaction mixture was stirred for 12 h at r.t., diluted with dichloromethane (300 mL) and washed with satd. aq. $NaHCO_3$ (2×50 mL). The organic phase was dried over Na_2SO_4, filtered and concentrated. The residue was purified by MPLC (dichloromethane/acetone, 1:0 → 20:1) to give **8** (2.66 g, 75 %) as a solid. R_f = 0.29 (dichloromethane/acetone 20:1); $[\alpha]_D^{20}$ = -25 (*c* = 1.8 in chloroform).^{1}H NMR (600 MHz, $CDCl_3$, TMS): δ = 7.41 (ad, J = 8.8 Hz, 2 H, m-CH-Ar), 6.90 (ad, 2 H, o-CH-Ar), 5.48 (s, 1 H, O-CH-O anisylidene), 5.15 (d, $J_{2,NH}$ = 5.6 Hz, 1 H, N-H), 4.83 (d, $J_{2,1}$ = 6.8 Hz, 1 H, H-1), 4.73 (d, J_{gem} = 11.8 Hz, 1 H, OCH_{2a} Troc), 4.66 (d, 1 H, OCH_{2b} Troc), 4.27 (dd, J_{gem} = 10.5 Hz, $J_{5,6a}$ = 4.9 Hz, 1 H, H-6a), 4.04-3.93 (m, 1 H, H-3), 3.80 (s, 3 H, OMe), 3.75 (t, $J_{5,6b}$ = 10.3 Hz, 1 H, H-6b), 3.53 (t, $J_{3,4}$ = $J_{5,4}$ = 9.2 Hz, 1 H, H-4), 3.47-3.40 (m, 1 H, H-5), 3.40-3.31 (m, 1 H, H-2), 2.98 (bs, 1H, OH), 0.89 (s, 9 H, *t*Bu), 0.12, 0.10 (2 s, 6 H, 2 x Si-Me); ^{13}C NMR (150 MHz, $CDCl_3$, TMS): δ = 160.46 (C_q anisylidene), 154.56 (CO Troc), 129.69 (p-C_q anisylidene), 127.86 (m-CH anisylidene), 113.89 (o-CH anisylidene), 101.99 (O-CH-O anisylidene), 96.34 (C-1), 95.37 (CCl_3 Troc), 81.65 (C-4), 74.95 (CH_2 Troc), 70.84 (C-3), 68.75 (C-6), 66.37 (C-5), 60.92 (C-2), 55.47 (OMe), 25.74 (*t*Bu), 25.72 (CH_3 *t*Bu), -4.02, -5.14 (Si-CH_3); HRMS ($^+$ESI-TOF): calcd. for $C_{23}H_{34}Cl_3NNaO_8Si$ *m/z* $[M+Na]^+$: 608.1011, found 608.1016.

Synthesis of fatty acids 9-11

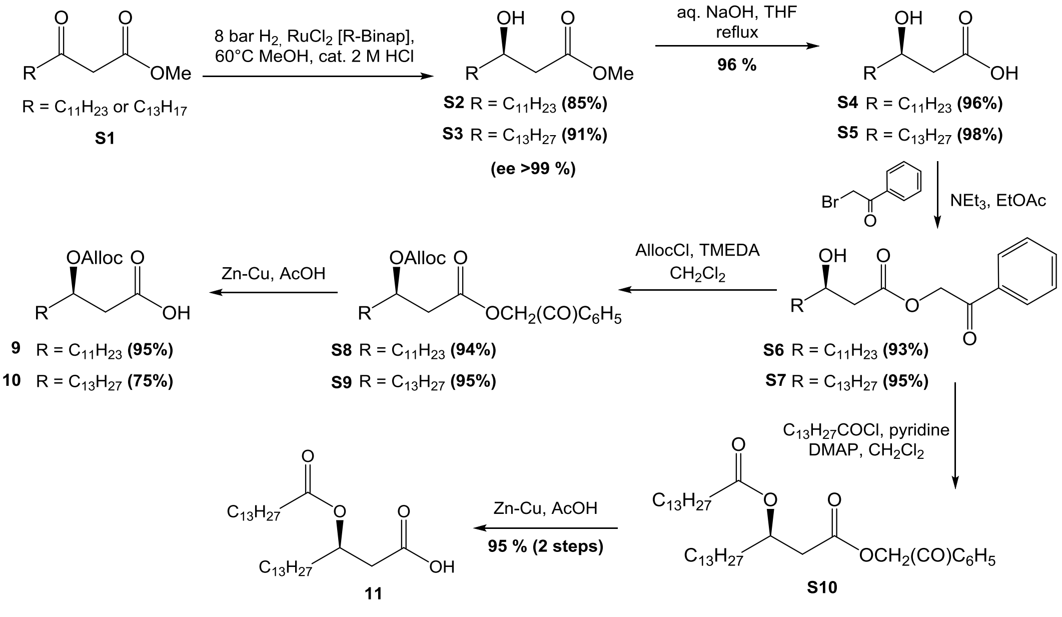

Synthesis of the Alloc protected (*R*)-3-hydroxy fatty acids **9** and **10** and the acyloxyacyl fatty acid **11** was performed starting from the corresponding β-ketoesters **S1** and relied on the known protocols.[3] The β-ketoesters were synthesized in multigram amounts by use of the Meldrum's acid method,[4] starting from the corresponding commercially available acid chlorides. The first step of the sequence was the asymmetric *Noyori* hydrogenation[5] of β-ketoesters. Addition of catalytic amount of hydrochloric acid substantially increased the hydrogenation rate, such that the reaction could be carried out at lower temperatures (60 °C) and under lower hydrogen pressure (8 bar).[6,7] Upon application of commercially available catalyst (Sigma-Aldrich) the hydrogenation did not work in our hands. Therefore, the ruthenium catalyst was freshly prepared as described.[6] The catalyst could be stored under argon at -20°C for at least 1 month without loss of activity. The reactions were performed under 8 bar of hydrogen, at 60°C in methanol for 15 h. in the presence of the ruthenium catalyst and a catalytic amount of hydrochloric acid starting with 10 g of β-ketoester to afford **S2** and **S3** which were purified by crystallisation from hexane. Enantiomeric purity was determined as described.[3] The introduction of Alloc protecting groups to phenacyl esters **S6** and **S7** was performed by reaction with AllocCl in the presence of TMEDA to afford **S8** and **S9**. Finally the phenacyl ester was cleaved using freshly prepared zinc-copper couple to give the fatty acids **9** and **10** in a high overall yield. Similarly, (*R*)-3-(tetradecanoyloxy)hexadecanoic acid **11** was prepared, starting from **S7** by reaction with tetradecanoyl chloride as reported.

Phenacyl (*R*)-3-(allyloxycarbonyloxy)tetradecanoate (S8)

Phenacyl (*R*)-3-hydroxytetradecanoate **S6** (2.47 g, 6.82 mmol) was dissolved in dry dichloromethane (45 mL) and tetramethylethylenediamine (TMEDA) (1.52 mL, 10.23 mmol) was added. The mixture was cooled down to 0°C, then allyl chloroformate (2.16 mL, 20.45 mmol) was slowly added and the reaction was stirred at r.t. for 3 h. The mixture was diluted with dichloromethane (200 mL) and was washed with 2 N HCl (100 mL) and satd. $NaHCO_3$ (100 mL). The organic phase was dried over Na_2SO_4, filtered and concentrated. The residue was purified by flash chromatography on silica gel (n-hexane/ethyl acetate, 5:1, dry-loading after dissolution in DCM) to give **S8** (2.98 g, 94%) as colorless oil. R_f = 0.61 (toluene/ethyl acetate 9:1; $[\alpha]_{20}^{D}$ = - 2 (*c* = 3.3 in chloroform); ^{1}H NMR (300 MHz, $CDCl_3$): *δ* = 7.92-7.84 (m, 2 H, 2 x o-CH), 7.63-7.55 (m, 1 H, p-CH), 7.51-7.43 (m, 2 H, 2 x m-CH), 6.01-5.86 (m, 1 H, =CH Allyl), 5.39 (d, J_{gem} = 16.4 Hz, 1 H, α-CH_a phenacyl), 5.35 (qd, J_{gem} = J_{allyl} = 1.6 Hz, J_{trans} = 17.2 Hz, 1 H, =CH_2 trans Allyl), 5.30 (d, 1 H, α-CH_b phenacyl), 5.25 (qd, J_{cis} = 10.4 Hz, 1 H, =CH_2 cis Allyl), 5.17 (m, 1 H, β-CH), 4.66-4.60 (m, 2 H, -O-CH_2 Alloc), 2.86 (dd, J_{gem} = 15.8 Hz, J_{vic} = 7.3 Hz, 1 H, α-CH_a), 2.76 (dd, J_{vic} = 5.6 Hz, 1 H, α-CH_b), 1.80-1.62 (m, 2 H, γ-CH_2), 1.47-1.15 (m, 18 H, CH_2), 0.87 (t, *J* = 6.7 Hz, 3 H, ω-CH_3); ^{13}C NMR (75 MHz, $CDCl_3$): *δ* = 191.77 (C=O phenacyl), 169.78 (COO), 154.60 (C_q Alloc), 134.29 (i-C_q), 133.99 (p-CH), 131.81 (=CH Alloc), 128.97 (m-CH), 127.86 (o-CH), 118.82 (=CH_2 Alloc), 74.83 (β-CH), 68.57 (-OCH_2 Alloc), 66.28 (α-CH_2 phenacyl), 38.88 (α-CH_2), 34.33 (γ-CH_2), 32.02, 29.73, 29.72, 29.63, 29.54, 29.45, 29.43, 25.07, 22.79 (9 x CH_2), 14.22 (ω-CH_3); HRMS calcd. for $C_{26}H_{38}NaO_6$ *m/z* $[M+Na]^+$: 469.2561, found 469.2560.

Phenacyl (*R*)-3-(allyloxycarbonyloxy)hexadecanoate (S9)

S9 was prepared similarly to **S8** starting with phenacyl (*R*)-3-hydroxyhexadecanoate **S7** (6.24 g, 15.99 mmol). Yield: 7.20 g (95%) as colorless oil. R_f = 0.63 (toluene/ethyl acetate 9:1); $[\alpha]_{20}^{D}$ = - 2 (*c* = 2.6 in chloroform); ^{1}H NMR (300 MHz, $CDCl_3$): *δ* = 7.92-7.87 (m, 2 H, 2 x o-CH), 7.63-7.57 (m, 1 H, p-CH), 7.51-7.44 (m, 2 H, 2 x m-CH), 6.01-5.87 (m, 1 H, =CH Allyl), 5.39 (d, J_{gem} = 16.4 Hz, 1 H, α-CH_a phenacyl), 5.35 (qd, J_{gem} = J_{allyl} = 1.5 Hz, J_{trans} = 17.2 Hz, 1 H, =CH_2 trans Allyl), 5.30 (d, 1 H, α-CH_b phenacyl), 5.25 (qd, J_{cis} = 10.4 Hz, 1 H, =CH_2 cis Allyl), 5.17 (m, 1 H, β-CH), 4.65-4.61 (m, 2 H, OCH_2 Alloc), 2.86 (dd, J_{gem} = 15.9 Hz, J_{vic} = 7.4 Hz, 1 H, α-CH_a), 2.76 (dd, J_{vic} = 5.6 Hz, 1 H, α-CH_b), 1.77-1.60 (m, 2 H, γ-CH_2), 1.45-1.18 (m, 22 H, CH_2), 0.88 (t, *J* = 6.7 Hz, 3 H, ω-CH_3); ^{13}C NMR (75 MHz, $CDCl_3$): *δ* = 191.79 (C=O phenacyl), 169.80 (CO), 154.62 (C_q Alloc), 134.32 (C_q), 134.01 (p-CH), 131.82 (=CH Alloc), 128.99 (m-CH), 127.88 (o-CH), 118.86 (=CH_2 Alloc), 74.85 (β-CH), 68.55 (OCH_2 Alloc), 66.30 (α-CH_2 phenacyl), 38.90 (α-CH_2), 34.11 (γ-CH_2), 32.05, 29.81, 29.79, 29.77, 29.76, 29.66, 29.57, 29.48, 29.45, 25.09, 22.81 (11 x CH_2), 14.24 (ω-CH_3); HRMS calcd. for $C_{28}H_{42}NaO_6$ *m/z* $[M+Na]^+$: 497.2874, found 497.2871.

(*R*)-3-(allyloxycarbonyloxy)tetradecanoic acid (9)

Preparation of a Zn-Cu-couple: Zinc (15.23 g, 0.23 mol) was suspended in deionized water (50 mL) and a 5% solution of $CuSO4{\cdot}5H_2O$ (5.33 g, 21.34 mmol in 107 mL H_2O) was added and the mixture was stirred for 5 min at r.t. The solid were separated on the filter, washed with water (50 mL), ethanol (50 mL) and diethyl ether (50 mL). Phenacyl (*R*)-3-(allyloxycarbonyloxy)tetradecanoate **S8** (2.91 g, 6.53 mmol) was dissolved in a mixture of toluene (95 mL) and glacial acetic acid (62 mL). The freshly prepared Zn-Cu-couple was added to the solution and the reaction was stirred at r.t. for 4 h. The solids were removed by filtration over Celite. The filtrate was evaporated and redissolved in dichloromethane (200 mL). The mixture was washed with 2 M HCl (50 mL) and deionized water (50 mL). The organic layer was dried over Na_2SO_4, filtered and concentrated. The residue was purified by flash chromatography on silica gel (1. hexane/ethyl acetate 5:1; 2. hexane/ethyl acetate + 1% acetic acid) to afford **9** (2.03 g, 95%) of as colorless oil. R_f = 0.1 (toluene/ethyl acetate 3:1); $[\alpha]_{20}^{D}$ = -0.1 (*c* = 0.9 in chloroform); ^{1}H NMR (300 MHz, $CDCl_3$): *δ* = 5.93 (m, 1 H, =CH Allyl), 5.35 (qd, J_{gem} = J_{allyl} = 1.5 Hz, 1 H, =CH_2 trans Allyl), 5.25 (qd, 1 H, =CH_2 cis Allyl), 5.08 (m, 1 H, β-CH), 4.65-4.59 (m, 2 H, -O-CH_2 Alloc), 2.72 (dd, J_{gem} = 16.2 Hz, J_{vic} = 7.4 Hz, 1 H, α-CH_a), 2.61 (dd, J_{vic} = 5.4 Hz, 1 H, α-CH_b), 1.79-1.56 (m, 2 H, γ-CH_2), 1.42-1.17 (m, 18 H, CH_2), 0.88 (t, *J* = 6.7 Hz, 3 H, ω-CH_3); ^{13}C NMR (75 MHz, $CDCl_3$): *δ* = 175.70 (COO), 154.62 (C_q Alloc), 131.69 (=CH Alloc), 118.98 (=CH_2 Alloc), 74.61 (β-CH), 68.60 (OCH_2 Alloc), 38.82 (α-CH_2), 34.11 (γ-CH_2), 32.05, 29.75, 29.75, 29.66, 29.58, 29.47, 29.46, 25.10, 22.82 (9 x CH_2), 14.25 (ω-CH_3); HRMS calcd. for $C_{18}H_{32}O_5$ *m/z* $[M-H]^-$: 327.2177, found 327.2179.

(*R*)-3-(allyloxycarbonyloxy)hexadecanoic acid (10)

10 was prepared similarly to **9** starting with phenacyl (*R*)-3-(allyloxycarbonyloxy)hexadecanoate **S9** (7.13 g, 15.02 mmol). Yield: 3.99 g (75%) as colorless oil. R_f = 0.32 (n-hexane/ethyl acetate 3:1 + 1% AcOH); $[\alpha]_{20}^{D}$ = -0.6 (*c* = 0.6 in chloroform); ^{1}H NMR (300 MHz,

$CDCl_3$): δ = 5.93 (m, 1 H, =CH Allyl), 5.35 (qd, J_{gem} = J_{allyl} = 1.5 Hz, 1 H, =CH_2 trans Allyl), 5.2 (qd, 1 H, =CH_2 cis Allyl), 5.08 (m, 1 H, β-CH), 4.65-4.60 (m, 2 H, -O-CH_2 Alloc), 2.73 (dd, J_{gem} = 16.3 Hz, J_{vic} = 7.4 Hz, 1 H, α-CH_a), 2.62 (dd, J_{vic} = 5.3 Hz, 1 H, α-CH_b), 1.79-1.57 (m, 2 H, γ-CH_2), 1.43-1.17 (m, 22 H, CH_2), 0.88 (t, J = 6.7 Hz, 3 H, ω-CH_3); ^{13}C NMR (75 MHz, $CDCl_3$): δ = 176.21 (CO), 154.60 (C_q Alloc), 131.68 (=CH Alloc), 118.99 (=CH_2 Alloc), 74.53 (β-CH), 68.60 (OCH_2 Alloc), 38.83 (α-CH_2), 34.11 (γ-CH_2), 32.07, 29.83, 29.80, 29.79, 29.76, 29.67, 29.59, 29.50, 29.46, 25.10, 22.84 (11 x CH_2), 14.26 (ω-CH_3); HRMS calcd. for $C_{20}H_{35}O_5$ *m/z* $[M-H]^-$: 355.2490, found 355.2489.

(*R*)-3-(tetradecanoyloxy)hexadecanoic acid (11)

Compound **S7** (5.89 g, 15.08 mmol) was dissolved in dry dichloromethane (70 mL), pyridine (7.4 mL, 91.58 mmol) and DMAP (10 mg) were added. The mixture was cooled down to 0°C and myristoyl chloride (3.72 mL, 15.08 mmol) was slowly added and the reaction was stirred at r.t. for 15 h. The reaction was quenched by addition of methanol (2.5 mL) and the stirring was continued for another 30 min. The mixture was diluted with ethyl acetate (300 mL) and washed with 2 M HCl (100 mL) and brine (50 mL). The organic layer was dried over Na_2SO_4, filtered and concentrated to give **S10** as slightly yellowish solid. Cleavage of the phenacyl ester was performed similar to the preparation of **9**. The crude product was purified by silica gel column chromatography (1. toluene/ethyl acetate 6:1; 2. dichloromethane/methanol 85:15) to give **11** (5.62 g, 93%) as a colorless oil. R_f = 0.35 (toluene/ethyl acetate 10:1); $[\alpha]_{20}^{D}$ = -0.3 (c = 1.4 in chloroform); ^{1}H NMR (600 MHz, $CDCl_3$): δ = 5.23-5.18 (m, 1 H, β-CH), 2.62 (dd, J_{gem} = 15.8 Hz, J_{vic} = 7.3 Hz, 1 H, α-CH_a), 2.62 (dd, J_{vic} = 5.4 Hz, 1 H, α-CH_b), 2.27 (t, J = 7.5 Hz, 2 H, α-CH_2 ^{tetradecanoate}), 1.67-1.55 (m, 4 H, γ-CH_2, β-CH_2 ^{tetradecanoate}), 1.38-1.11 (m, 42 H, CH_2), 0.88 (at, J = 7.0 Hz, 6 H, 2 x ω-CH_3). ^{13}C NMR (150 MHz, $CDCl_3$): δ = 176.14 (COOH), 173.43 (CO ^{tetradecanoate}), 70.16 (β-CH), 39.04 (α-CH_2), 34.64 (γ-CH_2), 34.14 (α-CH_2 ^{tetradecanoate}), 32.07, 29.84, 29.82, 29.79, 29.70, 29.64, 29.51, 29.43, 29.28, 29.26, 25.16, 22.84 (CH_2), 14.24 (ω-CH_3); HRMS (ESI) calcd. for $C_{30}H_{57}O_4$ *m/z* $[M-H]^-$: 481.4262, found 481.4267.

Synthesis of 12

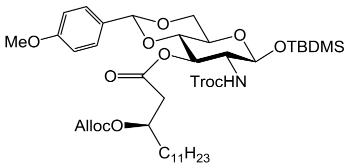

***tert*-Butyldimethylsilyl 3-*O*-[(*R*)-3-(allyloxycabonyloxy)tetradecanoyl]-2-deoxy-4,6-*O*-(*p*-methoxybenzylidene)-2-(2,2,2-trichloroethoxycarbonylamino)-β-D-glucopyranoside (12)**

To a stirred solution of **8** (3.02 g, 5.14 mmol) and fatty acid **9** (2.03 g, 6.17 mmol) in dry dichloromethane (40 mL), DMAP (13 mg) was added and the mixture was cooled to 0°C. Then a solution of *N,N′*-diisopropylcarbodiimide (DIC) (955 µL, 6.17 mmol) in dry dichloromethane (4 mL) was added dropwise and the reaction was stirred at 0°C for 5 hours. The reaction mixture was filtered, the filtrate was diluted with dichloromethane (200 ml) and washed with 1 M aq. HCl (20 mL), water (50 mL) and satd. aq. $NaHCO_3$ (2×50 mL), dried over Na_2SO_4, filtered and concentrated. The residue was purified by MPLC (toluene/ethyl acetate, 50:1 → 15:1) to provide **12** (4.47 g, 97%) as a syrup. R_f = 0.31 (toluene/ethyl acetate 15:1); $[\alpha]_{20}^{D}$ = -14.0 (c = 0.9 in chloroform); ^{1}H NMR (600 MHz, $CDCl_3$, TMS): δ = 7.35 (ad, J = 8.7 Hz, 2 H, m-CH-Ar), 6.87 (ad, 2 H, o-CH-Ar), 5.91 (m, 1 H, =CH Alloc), 5.45 (s, 1 H, O-CH-O anisylidene), 5.39 (at, $J_{4,3}$ = $J_{2,3}$ = 9.9 Hz, 1 H, H-3), 5.34 (qd, J_{gem} = J_{allyl} ~ 1.5 Hz, 1 H, =CH_2 trans Alloc), 5.26 (qd, 1 H, =CH_2 cis Alloc), 5.24 (d, $J_{2,NH}$ ~ 7.2 Hz, 1 H, NH), 5.03 (m, 1 H, β-CH^{Myr}), 4.95 (d, $J_{2,1}$ = 7.8 Hz, 1 H, H-1), 4.74 (d, J_{gem} = 12.0 Hz, 1 H, OCH_{2a} Troc), 4.62 (d, 1 H, OCH_{2b} Troc), 4.61 (td, J_{gem} = 13.2 Hz, 1 H, -O-CH_{2a} Alloc), 4.57 (td, 1 H, -O-CH_{2b} Alloc), 4.29 (dd, $J_{6a,6b}$ = 10.6 Hz, $J_{5,6a}$ = 5.0 Hz, 1 H, H-6a), 3.79 (s, 3 H, -OMe), 3.78 (t, $J_{5,6b}$ = 10.3 Hz, 1 H, H-6b), 3.67 (t, $J_{5,4}$ 0 9.5 Hz, 1 H, H-4), 3.54-3.47 (m, 2 H, H-5, H-2), 2.68 (dd, J_{gem} = 15.5 Hz, J_{vic} = 6.7 Hz, 1 H, α-CH_a^{Myr}), 2.60 (dd, J_{vic} = 5.8 Hz, 1 H, α-CH_b^{Myr}), 1.67-1.54 (m, 2 H, γ-CH_2^{Myr}), 1.35-1.14 (m, 18 H, CH_2^{Myr}), 0.90-0.86 (m, 12 H, *t*Bu, ω-CH_3^{Myr}), 0.12, 0.10 (2 s, 6 H, 2 x Si-Me); ^{13}C NMR (150 MHz, $CDCl_3$): δ = 169.87 (CO^{Myr}), 160.31 (C_q anisylidene), 154.58 (C_q Alloc), 154.16 (CO Troc), 131.69 (=CH Alloc), 129.55 (p-C_q anisylidene), 127.68 (m-CH anisylidene), 119.11 (=CH_2 Alloc), 113.76 (o-CH anisylidene), 101.61 (O-CH-O anisylidene), 96.68 (C-1), 95.44 (CCl_3 Troc), 78.94 (C-4), 74.82 (CH_2 Troc), 74.77 (β-CH^{Myr}), 71.27 (C-3), 68.73 (C-6), 68.61 (OCH_2 Alloc), 66.60 (C-5), 59.42 (C-2), 55.41 (OMe), 39.05 (α-CH_2^{Myr}), 33.86 (γ-CH_2^{Myr}), 32.06, 29.78, 29.76, 29.69, 29.64, 29.49, 29.44 (7 x CH_2^{Myr}), 25.70 (C_q *t*Bu), 25.67 (CH_3 *t*Bu), 25.11, 22.83 (2 x CH_2^{Myr}), 14.26 (ω-CH_3^{Myr}), -4.09, -5.15 (Si-CH_3); HRMS ($^-$ESI-TOF): calcd. for $C_{41}H_{63}Cl_3NO_{12}Si$ *m/z* $[M-H]^-$: 894.3191; found 894.3190.

Synthesis of 13 and 14

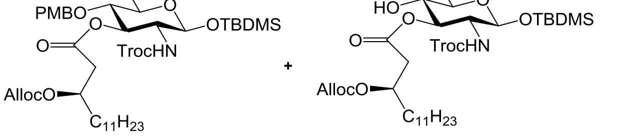

13 **14**

***tert*-Butyldimethylsilyl 3-*O*-[(*R*)-3-(allyloxycarbonyloxy)tetradecanoyl]-2-deoxy-4-*O*-*p*-methoxybenzyl-2-(2,2,2-trichloroethoxycarbonylamino)-β-D-glucopyranoside (13) and**

t*ert*-butyldimethylsilyl 3-*O*-[(*R*)-3-(allyloxycarbonyloxy)tetradecanoyl)]-2-deoxy-6-*O*-*p*-methoxybenzyl-2-(2,2,2-trichloroethoxycarbonylamino)-β-D-glucopyranoside (14)

To a solution of **12** (2.96 g, 3.30 mmol) in dry acetonitrile (70 mL) powdered molecular sieves (3 Å, 3 g) were added and the suspension was stirred for 1 h at r.t. under Ar. Then sodium cyanoborohydride (2.49 g, 39.61 mmol) was added and the mixture was cooled to 0°C and TMSCl (5.06 mL, 39.61 mmol) was added drowpwise via syringe and stirring was continued for 6 h at r.t. under Ar. The reaction was cooled to 0°C and quenched by addition of satd. aq. $NaHCO_3$ (50 mL). The mixture was diluted with ethyl acetate (50 mL) and filtered over a pad of Celite, the solids were washed with ethyl acetate (150 mL). The organic phase was washed with 1 M HCl (80 mL), followed by satd. aq. $NaHCO_3$ (2×50 mL) and brine (100 mL), dried over Na_2SO_4, filtered and concentarted. The residue was purified by chromatography on silica gel (dichloromethane/acetone, 30:1) to give an inseparable mixture **13** + **14** (**13 : 14** = 60 : 40 according to ^{1}H-NMR, 2.63 g, 90%) as a syrup. Small portions of **13** and **14** were isolated in pure form and characterized by NMR. **13:** R_f = 0.33 (dichloromethane/acetone 30:1); $[\alpha]_{20}^{D}$ = +1 (*c* = 1.5 in chloroform); ^{1}H NMR (600 MHz, $CDCl_3$, TMS): *δ* = 7.20 (ad, *J* = 8.6 Hz, 2 H, m-CH-Ar), 6.86 (ad, 2 H, o-CH-Ar), 5.91 (m, 1 H, =CH Alloc), 5.34 (qd, J_{gem} = J_{allyl} ~ 1.4 Hz, 1 H, =CH_2 trans Alloc), 5.25 (qd, 1 H, =CH_2 cis Alloc), 5.22 (dd, $J_{4,3}$ = 9.2 Hz, $J_{2,3}$ = 10.5 Hz, 1 H, H-3), 5.13 (d, $J_{2,NH}$ = 9.2 Hz, 1 H, NH), 5.07-5.02 (m, 1 H, β-CH^{Myr}), 4.78 (d, $J_{2,1}$ = 7.9 Hz, 1 H, H-1), 4.73 (d, J_{gem} = 12.0 Hz, 1 H, OCH_{2a} Troc), 4.64-4.54 (m, 2 H, OCH_2 Alloc, 1 H, OCH_{2b} Troc, 1 H, OCH_{2a} PMB), 4.52 (d, J_{gem} = 11.6 Hz, 1 H, OCH_{2b} PMB), 3.83 (ddd, $J_{6a,b}$ = 12.0 Hz, $J_{5,6a}$ = 2.7 Hz, $J_{6\text{-}OH,6a}$ = 5.3 Hz, 1 H, H-6a), 3.79 (s, 3 H, -OMe), 3.70 (ddd, $J_{5,6b}$ = 4.2 Hz, $J_{6\text{-}OH,6b}$ = 8.0 Hz, 1 H, H-6b), 3.66 (at, $J_{5,4}$ = 9.5 Hz, 1 H, H-4), 3.50 (aq, 1 H, H-2), 3.41 (ddd, 1 H, H-5), 2.59 (dd, J_{gem} = 16.3 Hz, J_{vic} = 7.6 Hz, 1 H, α-CH_a^{Myr}), 2.51 (dd, J_{vic} = 4.9 Hz, 1 H, α-CH_b^{Myr}), 1.82 (dd, 1 H, 6-OH), 1.69-1.54 (m, 2 H, γ-CH_2^{Myr}), 1.46-1.19 (m, 18 H, CH_2^{Myr}), 0.91-0.83 (m, 12 H, *t*Bu, ω-CH_3^{Myr}), 0.11, 0.08 (2 s, 6 H, 2 x Si-Me); ^{13}C NMR (150 MHz, $CDCl_3$): *δ* = 170.30 (CO^{Myr}), 159.64 (C_q PMB), 154.58 (C_q Alloc), 154.20 (CO Troc), 131.72 (=CH Alloc), 129.94 (m-CH PMB), 129.82 (p-C_q PMB), 119.04 (=CH_2 Alloc), 114.11 (o-CH PMB), 96.34 (C-1), 95.49 (CCl_3 Troc), 75.35 (C-4, C-5), 74.77 (CH_2 Troc), 74.60, 74.55 (β-CH^{Myr}, C-3), 73.36 (OCH_2 PMB), 68.59 (OCH_2 Alloc), 62.03 (C-6), 58.78 (C-2), 55.39 (OMe), 38.92 (α-CH_2^{Myr}), 34.02 (γ-CH_2^{Myr}), 32.05, 29.76, 29.75, 29.68, 29.63, 29.49, 29.47 (7 x CH_2^{Myr}), 25.67 (C_q *t*Bu), 25.66 (CH_3 *t*Bu), 25.16, 22.82 (2 x CH_2^{Myr}), 14.24 (ω-CH_3^{Myr}), -3.99, -5.11 (Si-CH_3); HRMS ($^+$ESI-TOF): calcd. for $C_{41}H_{66}Cl_3NNaO_{12}Si$ *m/z* $[M+Na]^+$: 920.3312, found 920.3301.

14: R_f = 0.25 (dichloromethane/acetone 30:1); $[\alpha]_{20}^{D}$ = -8.3 (*c* = 1.56 in chloroform); ^{1}H NMR (600 MHz, $CDCl_3$, TMS): *δ* = 7.25 (ad, *J* = 8.6 Hz, 2 H, m-CH-Ar), 6.87 (ad, 2 H, o-CH-Ar), 5.90 (m, 1 H, =CH Alloc), 5.34 (qd, J_{gem} = J_{allyl} ~ 1.4 Hz, 1 H, =CH_2 trans Alloc), 5.25 (qd, 1 H, =CH_2 cis Alloc), 5.16 (d, $J_{2,NH}$ = 8.9 Hz, 1 H, N-H), 5.06 (dd, $J_{4,3}$ = 9.4 Hz $J_{2,3}$ = 10.3 Hz, 1 H, H-3), 5.03 (m, 1 H, β-CH^{Myr}), 4.76 (d, $J_{2,1}$ = 7.9 Hz, 1 H, H-1), 4.73 (d, J_{gem} = 12.0 Hz, 1 H, OCH_{2a} Troc), 4.65-4.55 (m, 1 H, OCH_{2b} Troc, 2 H, OCH_2 Alloc), 4.53 (d, J_{gem} = 11.6 Hz, 1 H, OCH_{2a} PMB), 4.50 (d, 1 H, OCH_{2b} PMB), 3.81 (s, 3 H, OMe), 3.74 (dd, J_{gem} = 10.5 Hz, $J_{5,6a}$ = 4.9 Hz, 1 H, H-6a), 3.72 (dd, $J_{5,6b}$ = 4.2 Hz, 1 H, H-6b), 3.67 (dd, $J_{3,4}$ = $J_{5,4}$ = 9.4 Hz, $J_{4,4\text{-}OH}$ = 3.3 Hz, 1 H, H-4), 3.55-3.49 (m, 2 H, H-2, H-5), 3.15 (d, 1 H, 4-OH), 2.68 (dd, J_{gem} = 15.4 Hz, J_{vic} = 7.8 Hz, 1 H, α-CH_a^{Myr}), 2.58 (dd, J_{vic} = 4.3 Hz, 1 H, α-CH_b^{Myr}), 1.72-1.57 (m, 2 H, γ-CH_2^{Myr}), 1.40-1.20 (m, 18 H, CH_2^{Myr}), 0.90-0.85 (m, 12 H, *t*Bu, ω-CH_3^{Myr}), 0.12, 0.09 (2 s, 6 H, 2 x Si-Me); ^{13}C NMR (150 MHz, $CDCl_3$): *δ* = 171.21 (CO^{Myr}), 159.47 (i-C_q PMB), 154.90 (C_q Alloc), 154.22 (CO Troc), 131.53 (=CH Alloc), 130.05 (p-C_q PMB), 129.42 (m-CH PMB), 119.22 (=CH_2 Alloc), 114.00 (o-CH PMB), 96.43 (C-1), 95.55 (CCl_3 Troc), 75.65 (C-3), 75.30 (β-CH^{Myr}), 74.75 (CH_2 Troc), 74.36 (C-5), 73.51 (OCH_2 PMB), 70.67 (C-4), 70.04 (C-6), 68.75 (OCH_2 Alloc), 58.12 (C-2), 55.42 (OMe), 39.65 (α-CH_2^{Myr}), 34.46 (γ-CH_2^{Myr}), 32.05, 29.76, 29.75, 29.66, 29.60, 29.47, 29.43 (7 x CH_2^{Myr}), 25.73 (C_q *t*Bu), 25.71 (CH_3 *t*Bu), 25.13, 22.82 (2 x CH_2^{Myr}), 14.25 (ω-CH_3^{Myr}), -3.99, -5.15 (Si-CH_3); HRMS ($^-$ESI-TOF): calcd. for $C_{41}H_{65}Cl_3NO_{12}Si$ *m/z* $[M-H]^-$ 896.3347; found 896.3353.

Synthesis of 15

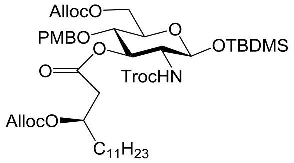

***tert*-Butyldimethylsilyl 6-*O*-allyloxycarbonyl-3-*O*-[(*R*)-3-(allyloxycarbonyloxy)tetradecanoyl]-2-deoxy-4-*O*-*p*-methoxybenzyl-2-(2,2,2-trichloroethoxycarbonylamino)-β-D-glucopyranoside (15)**

To a stirred solution of an inseparable mixture **13 + 14** (6:4, 2.67 g, 2.97 mmol) in dry dichloromethane (150 mL) 2,4,6-collidine (5.87 mL, 44.55 mmol) and allyloxycarbonyl chloride (AllocCl) (14.26 mL, 133.65 mmol) were added succesively at 0°C under Ar. The reaction mixture was stirred at 45°C under reflux for 15 h, then cooled to r.t. and diluted with dichloromethane (250 mL). The mixture was washed with 1 M HCl (3 x 100 mL), water (100 mL), satd. aq. $NaHCO_3$ (2 x 150 mL), dried over Na_2SO_4, filtered and concentrated. The residue was purified by MPLC (dichloromethane/ethyl acetate, 30:1 → 10:1) to afford **15** (1.95 g, 96 % based on the proportion of **13 RH235** in the starting mixture **13+14**) as a syrup and a recovered **14** (1.08 g, 91% recovery based on the proportion of **14** in the starting mixture **13+14**). **15**: R_f = 0.32 (dichloromethane/ethyl, acetate 30:1); $[\alpha]_{20}^{D}$ = +11 (*c*= 0.74 in chloroform); ^{1}H NMR (600 MHz, $CDCl_3$): *δ* = 7.19 (ad, *J* = 8.6 Hz, 2 H, m-CH-Ar), 6.86 (ad, 2 H, o-CH-Ar), 5.97-5.87 (m, 2 H, =CH Alloc), 5.39-5.31 (m, 2 H, =CH_2 trans Alloc), 5.29-5.22 (m, 3 H, =CH_2 cis Alloc, H-3), 5.10 (d, $J_{2,NH}$ = 9.2 Hz, 1 H, N-H), 5.08-5.03 (m, 1 H, β-CH^{Myr}), 4.76 (d, $J_{2,1}$ = 7.9 Hz, 1 H, H-1), 4.73 (d, J_{gem} = 11.9 Hz, 1 H, OCH_{2a} Troc), 4.65-4.55 (m, 2 H, OCH_2 Alloc, 1 H, OCH_{2b} Troc, 1 H, OCH_{2a} PMB), 4.47 (d, J_{gem} = 11.0 Hz, 1 H, OCH_{2b} PMB), 4.40 (dd, $J_{6a,b}$ = 11.5 Hz, $J_{5,6a}$ = 1.9 Hz, 1 H, H-6a), 4.24 (dd, $J_{5,6b}$ = 5.1 Hz, 1 H, H-6b), 3.79 (s, 3 H, OMe), 3.61 (at, $J_{5,4}$ = 9.6 Hz, 1 H, H-4), 3.57 (ddd, 1 H, H-5), 3.49 (aq, 1 H, H-2), 2.61 (dd, J_{gem} = 16.3 Hz, J_{vic} = 7.5 Hz, 1 H, α-CH_a^{Myr}), 2.54 (dd, J_{vic} = 4.8 Hz, 1 H, α-CH_b^{Myr}), 1.70-1.57 (m, 2 H, γ-CH_2^{Myr}), 1.37-1.22 (m, 18 H, CH_2^{Myr}), 0.88 (t, *J* = 7.0 Hz, 3 H, ω-CH_3^{Myr}), 0.88 (s, 3 H, *t*Bu),

0.09, 0.07 (2 s, 6 H, 2 x Si-Me); ^{13}C NMR (150 MHz, $CDCl_3$): δ = 170.19 (CO,^{Myr}), 159.70 (C_q PMB), 154.91, 154.59 (2 x C_q Alloc), 154.17 (CO Troc), 131.70, 131.66 (2 x =CH Alloc), 129.98 (m-CH PMB), 129.54 (p-C_q PMB), 119.14, 119.07 (2 x =CH_2 Alloc), 114.15 (o-CH PMB), 96.23 (C-1), 95.52 (CCl_3 Troc), 75.52 (C-4), 74.76 (CH_2 Troc), 74.65, 74.58 (β-CH^{Myr}; C-3), 73.33 (CH_2 PMB), 73.11 (C-5), 68.73, 68.63 (OCH_2 Alloc), 66.27 (C-6), 58.72 (C-2), 55.41 (OMe), 38.94 (α-CH_2^{Myr}), 34.03 (γ-CH_2^{Myr}), 32.06, 29.78, 29.77, 29.69, 29.64, 29.49 (CH_2^{Myr}), 25.70 (C_q t-Bu), 25.68 (CH_3 *t*Bu), 25.19, 22.83 (2 x CH_2^{Myr}), 14.26 (ω-CH_3^{Myr}), -4.08, -5.22 (Si-CH_3); HRMS ($^+$ESI-TOF):calcd. for $C_{45}H_{74}Cl_3N_2O_{14}Si$ *m/z* $[M+NH_4]^+$: 999.3969, found 999.3969.

Synthesis of 16

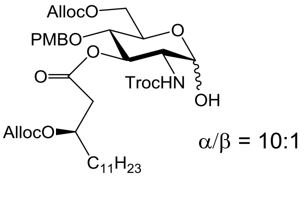

6-*O*-Allyloxycarbonyl-3-*O*-[(*R*)-3-(allyloxycarbonyloxy)tetradecanoyl]-2-deoxy-4-*O*-*p*-methoxybenzyl-2-(2,2,2-trichloroethoxycarbonylamino)-α-D-glucopyranose (16)

To a stirred solution of **15** (350 mg, 0.36 mmol) in dry THF (20 mL) in a PTFE flask a solution of Et_3N (148 µL, 1.07 mmol) in THF (1 mL) and triethylamine hydrofluoride (1.42 mL, 8.73 mmol) were added successively (pH 6) and the reaction mixture was stirred at r.t. for 48 h under Ar. The mixture was diluted with ethyl acetate (250 mL) and washed satd. aq. $NaHCO_3$ (2×50 mL) and water (50 mL). The organic phase was dried over Na_2SO_4, filtered and concentrated. The residue was purified by flash chromatography on silica gel (toluene/ethyl acetate, 4:1) to afford **16** (306 mg, 98 %) as a syrup. R_f = 0.23 (toluene/ethyl acetate, 5:1); $[\alpha]_D^{20}$ = +45 (*c* = 0.7 in chloroform); ^{1}H NMR (600 MHz, $CDCl_3$, TMS, α/β = 10:1, α-anomer) δ = 7.21 (ad, *J* = 8.5 Hz, 2 H, m-CH-Ar), 6.87 (ad, 2 H, o-CH-Ar), 5.98-5.86 (m, 2 H, =CH Alloc), 5.43-5.30 (m, 1 H, H-3, 1 H, N-H, 2 H, 2 x =CH_2 trans Alloc), 5.30-5.23 (m, 2 H, 2 x =CH_2 cis Alloc), 5.27 (d, $J_{2,1}$ = 3.7 Hz, 1 H, H-1), 5.10-5.05 (m, 1 H, β-CH^{Myr}), 4.71 (d, J_{gem} = 12.0 Hz, 1 H, OCH_{2a} Troc), 4.68 (d, 1 H, OCH_{2b} Troc), 4.65-4.54 (m, 5 H, OCH_2 Alloc, OCH_{2a} PMB), 4.37 (d, J_{gem} = 11.0 Hz, 1 H, OCH_{2b} PMB), 4.41 (dd, $J_{6a,b}$ = 11.6 Hz, $J_{5,6a}$ = 2.0 Hz, 1 H, H-6a), 4.25 (dd, $J_{5,6b}$ = 4.4 Hz, 1 H, H-6b), 4.11 (ddd, $J_{4,5}$ = 10.0 Hz, 1 H, H-5), 3.92 (ddd, $J_{NH,2}$ = 9.8 Hz, $J_{3,2}$ = 10.2 Hz, 1 H, H-2), 3.79 (s, 3 H, OMe), 3.61 (at, $J_{3,4}$ = 9.6 Hz, 1 H, H-4), 2.92 (d, 1 H, OH), 2.64 (dd, J_{gem} = 16.6 Hz, J_{vic} = 7.9 Hz, 1 H, α-CH_a^{Myr}), 2.54 (dd, J_{vic} = 4.8 Hz, 1 H, α-CH_b^{Myr}), 1.69-1.55 (m, 2 H, γ-CH_2^{Myr}), 1.37-1.22 (m, 18 H, CH_2^{Myr}), 0.88 (t, *J* = 7.0 Hz, 3 H, ω-CH_3^{Myr}); ^{13}C NMR (151 MHz, $CDCl_3$): δ = 170.51 (CO^{Myr}), 159.71 (C_q PMB), 154.92, 154.63 (2 x C_q Alloc), 154.48 (CO Troc), 131.82, 131.59 (2 x =CH Alloc), 130.05 (m-CH PMB), 129.55 (p-C_q PMB), 119.32, 118.96 (2 x =CH_2 Alloc), 114.18 (o-CH PMB), 95.54 (CCl_3 Troc), 91.94 (C-1), 75.15 (C-4), 74.80 (CH_2 Troc), 74.65 (OCH_2 PMB), 74.44 (β-CH^{Myr}), 73.35 (C-3), 69.19 (C-5), 68.90, 68.58 (OCH_2 Alloc), 66.12 (C-6), 55.41 (OMe), 54.72 (C-2), 38.93 (α-CH_2^{Myr}), 34.13 (γ-CH_2^{Myr}), 32.07, 29.78, 29.70, 29.65, 29.53, 29.49, 25.17, 22.84 (CH_2^{Myr}), 14.26 (ω-CH_3^{Myr}); HRMS ($^+$ESI-TOF): calcd. for $C_{39}H_{56}Cl_3NNaO_{14}$ *m/z* $[M+Na]^+$: 890.2659, found 890.2659.

Synthesis of 17

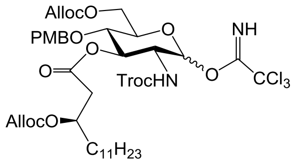

6-*O*-Allyloxycarbonyl-3-O-[(*R*)-3-(allyloxycarbonyloxy)tetradecanoyl]-2-deoxy-4-*O*-*p*-methoxybenzyl-2-(2,2,2-trichloroethoxycarbonylamino)-D-glucopyranosyl trichloroacetimidate (17)

To a stirred solution of **16** (604 mg, 0.69 mmol) in dry dichloromethane (20 mL) trichloroacetonitrile (1.39 mL, 13.90 mmol) and Cs_2CO_3 (204 mg, 0.63 mmol) were added succesively. The reaction mixture was stirred for 1 h at r.t. and filtered over a pad of Celite. The filtrate was diluted with dichloromethane (200 mL) and washed with satd. aq. $NaHCO_3$ (2 x 50 mL) and water (50 mL). The organic phase was dried over Na_2SO_4, filtered and concentrated. The residue was purified by flash chromatography on silica gel (hexane/ethylacetate, 3:1 supplemented with 0.1 % NEt_3) to give **17** (562 mg, 80 %) as a syrup. R_f = 0.55 (toluene/ethyl acetate, 5:1); $[\alpha]_D^{20}$ = +10 (*c* = 1.2 in chloroform); ^{1}H NMR (600 MHz, $CDCl_3$, TMS, α:β = 10:1, α-anomer) δ = 8.72 (s, 1 H, =NH), 7.22 (ad, *J* = 8.6 Hz, 2 H, m-CH-Ar), 6.87 (ad, 2 H, o-CH-Ar), 6.38 (d, $J_{2,1}$ = 3.6 Hz, 1 H, H-1), 5.98-5.87 (m, 2 H, =CH Alloc), 5.42 (dd, $J_{2,3}$ = 10.2 Hz, $J_{4,3}$ = 9.2 Hz, 1 H, H-3), 5.41-5.31 (m, 3 H, 2 x =CH_2 trans Alloc, NH), 5.31-5.24 (m, 2 H, 2 x =CH_2 cis Alloc), 5.12-5.07 (m, 1 H, β-CH^{Myr}), 4.74 (d, J_{gem} = 12.1 Hz, 1 H, OCH_{2a} Troc), 4.66-4.61 (m, 5 H, OCH_{2b} Troc, OCH_2 Alloc, OCH_{2a} PMB), 4.60-4.56 (m, 1 H, OCH_2 Alloc), 4.37 (d, J_{gem} = 10.8 Hz, 1 H, OCH_{2b} PMB), 4.39 (dd, $J_{6a,b}$ = 11.9 Hz, $J_{5,6a}$ = 2.1 Hz, 1 H, H-6a), 4.30 (dd, $J_{5,6b}$ = 3.2 Hz, 1 H, H-6b), 4.14 (ddd, $J_{NH,2}$ = 9.0 Hz, 1 H, H-2), 4.02 (ddd, $J_{4,5}$ = 10.0 Hz, 1 H, H-5), 3.84 (t, 1 H, H-4), 3.79 (s, 3 H, OMe), 2.66 (dd, J_{gem} = 16.5 Hz, J_{vic} = 8.1 Hz, 1 H, α-CH_a^{Myr}), 2.57 (dd, J_{vic} = 4.6 Hz, 1 H, α-CH_b^{Myr}), 1.71-1.54 (m, 2 H, γ-CH_2^{Myr}), 1.37-1.20 (m, 18 H, CH_2^{Myr}), 0.88 (t, *J* = 7.0 Hz, 3 H, ω-CH_3^{Myr}); ^{13}C NMR (151 MHz, $CDCl_3$): δ = 170.79 (CO^{Myr}), 160.76 (OC=NH), 159.84 (C_q PMB), 154.68, 154.37 (2 x C_q Alloc, CO Troc), 131.75, 131.55 (2 x =CH Alloc), 130.24 (m-CH PMB), 129.22 (p-C_q PMB), 119.35, 119.07 (2 x =CH_2 Alloc), 114.14 (o-CH PMB), 95.45 (CCl_3 Troc), 94.80 (C-1), 74.95 (CH_2 Troc), 74.78 (OCH_2 PMB), 74.37 (β-CH^{Myr}), 74.20 (C-4), 72.92 (C-3), 71.54 (C-5), 68.90, 68.66 (OCH_2 Alloc), 65.39 (C-6), 55.41 (OMe), 54.45 (C-2), 38.99 (α-CH_2^{Myr}), 34.15 (γ-CH_2^{Myr}), 32.06, 29.78, 29.69, 29.63, 29.52, 29.48, 25.18, 22.83 (CH_2^{Myr}), 14.26 (ω-CH_3^{Myr}); HRMS ($^+$ESI-TOF): calcd. for $C_{41}H_{56}Cl_6N_2NaO_{14}$ *m/z* $[M+Na]^+$: 1033.1755, found 1033.1744.

Synthesis of 18

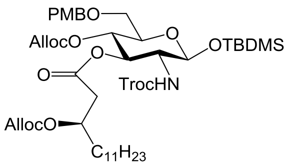

***tert*-Butyldimethylsilyl 4-*O*-allyloxycarbonyl-3-[(*R*)-3-(allyloxycarbonyloxy)tetradecanoyl]-2-deoxy-6-*O*-*p*-methoxy-benzyl-2-(2,2,2-trichloroethoxycarbonylamino)-β-D-glucopyranoside (18)**

To a stirred solution of **14** (1.94 g, 2.15 mmol) and tetramethylethylenediamine (TMEDA) (1.28 mL, 8.62 mmol) in dry dichloromethane (50 mL) allyl chloroformate (2.28 mL, 21.54 mmol) was added dropwise via syringe at 0 °C under Ar. The reaction mixture was stirred at r.t. for 3 h., diluted with dichloromethane (300 mL) and washed with 1 M HCl (2 x 100 mL), followed by satd. aq. $NaHCO_3$ (100 mL) and water (100 mL). The organic phase was dried over Na_2SO_4, filtered and concentrated. The residue was purified by chromatography on silica gel (dichloromethane/ethyl acetate, 30:1) to provide **18** (2.0 g, 95%) as a syrup. R_f = 0.18 (toluene/ethyl acetate 30:1); $[\alpha]_{20}^{D}$ = +12 (*c* = 2.2 in chloroform); ^{1}H NMR (600 MHz, $CDCl_3$): *δ* = 7.23 (ad, *J* = 8.8 Hz, 2 H, m-CH-Ar), 6.86 (ad, 2 H, o-CH-Ar), 5.92 (m, 1 H, =CH Alloc), 5.85 (m, 1 H, =CH Alloc), 5.36-5.30 (m, 1 H, H-3), 5.35 (qd, J_{gem} = J_{allyl} ~ 1.5 Hz, 1 H, =CH_2 trans Alloc), 5.30 (qd, J_{gem} = J_{allyl} ~ 1.5 Hz, 1 H, =CH_2 trans Alloc), 5.26 (qd, 1 H, =CH_2 cis Alloc), 5.23 (m, 1 H, NH), 5.23 (qd, 1 H, =CH_2 cis Alloc), 5.01 (m, 1 H, β-CH^{Myr}), 4.89 (d, $J_{2,1}$ = 7.8 Hz, 1 H, H-1), 4.89 (dd, $J_{5,4}$ = 9.9 Hz, $J_{3,4}$ = 9.2 Hz, 1 H, H-4), 4.72 (d, J_{gem} = 11.8 Hz, 1 H, OCH_{2a} Troc), 4.66-4.50 (m, 1 H, OCH_{2b} Troc, 4 H, OCH_2 Alloc), 4.49 (d, J_{gem} = 11.7 Hz, 1 H, OCH_{2a} PMB), 4.45 (d, 1 H, OCH_{2b} PMB), 3.80 (s, 3 H, OMe), 3.72-3.66 (m, 1 H, H-5), 3.60 (dd, $J_{6a,6b}$ = 10.6 Hz, $J_{5,6a}$ = 3.2 Hz, 1 H, H-6a), 3.58 (dd, $J_{5,6b}$ = 4.9 Hz, 1 H, H-6b), 3.53-3.47 (aq, 1 H, H-2), 2.68 (dd, J_{gem} = 16.0 Hz, J_{vic} = 6.9 Hz, 1 H, α-CH_a^{Myr}), 2.58 (dd, J_{vic} = 5.5 Hz, 1 H, α-CH_b^{Myr}), 1.67-1.54 (m, 2 H, γ-CH_2^{Myr}), 1.37-1.20 (m, 18 H, CH_2^{Myr}), 0.89-0.86 (m, 12 H, *t*Bu, ω-CH_3^{Myr}), 0.13, 0.09 (2 s, 6 H, 2 x Si-Me); ^{13}C NMR (151 MHz, $CDCl_3$): *δ* = 169.93 (CO^{Myr}), 159.34 (C_q PMB), 154.57 (C_q Alloc), 154.08 (CO Troc), 154.02 (C_q Alloc), 131.70 (=CH Alloc), 131.47 (=CH Alloc), 130.13 (p-C_q PMB), 129.34 (m-CH PMB), 119.14 (=CH_2 Alloc), 119.08 (=CH_2 Alloc), 113.89 (o-CH PMB), 95.90 (C-1), 95.48 (CCl_3 Troc), 74.74 (β-CH^{Myr}), 74.57 (CH_2 Troc), 73.57 (OCH_2 PMB), 73.33 (C-4), 73.08 (C-5), 72.33 (C-4), 69.01 (C-6), 68.90 (OCH_2 Alloc), 68.61 (OCH_2 Alloc), 58.74 (C-2), 55.39 (OMe), 38.80 (α-CH_2^{Myr}), 33.96 (γ-CH_2^{Myr}), 32.05, 29.77, 29.75, 29.68, 29.63, 29.47, 29.46 (7 x CH_2^{Myr}), 25.70 (C_q *t*Bu), 25.70 (CH_3 *t*Bu), 25.13, 22.82 (2 x CH_2^{Myr}), 14.25 (ω-CH_3^{Myr}), -4.02, -5.16 (Si-CH_3); HRMS ($^-$ESI-TOF): calcd. for $C_{45}H_{69}Cl_3NO_{14}Si$ *m/z* $[M-H]^-$: 980.3558, found 980.3559.

Synthesis of 19

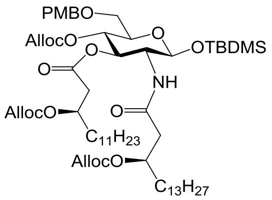

***tert*-Butyldimethylsilyl 4-*O*-allyloxycarbonyl-2-[(*R*)-3-(allyloxycarbonyloxy)hexadecanoylamino]-3-*O*-[(*R*)-3-(allyloxycarbonyloxy)tetradecanoyl]-2-deoxy-6-*O*-*p*-methoxybenzyl-β-D-glucopyranoside (19)**

To a stirred solution of **18** (1 g, 1.02 mmol) in dioxane/acetic acid (2:1, 30 mL) Zn dust (10 µm, 4 g, 61.2 mmol) was added and the mixture was stirred for 1h upon sonication every 10 min. The reaction mixture was filtered over a pad of Celite and the solids were washed with ethyl acetate washing (300 mL). The mixture was washed with water (100 mL), followed by satd. aq. $NaHCO_3$ and brine (2 x 50 mL). The organic layer was dried over Na_2SO_4, filtered and concentrated. The residue was dried by repeated reconstitution in dry toluene (3 x 50 mL) and concentration. The residue was dissolved in dry dichloromethane (50 mL) and a solution of fatty acid **10** (436 mg, 1.22 mmol) in dry dichloromethane (5 mL) was added under Ar. Then the stirred mixture was cooled to 0°C and *N,N´*-diisopropylcarbodiimide (190 µL, 1.22 mmol) was added and the reaction was stirred for 15 h at 4°C. Then another portion of *N,N´*-diisopropylcarbodiimide (50 µL, 0.32 mmol) and fatty acid **10** (140 mg, 0.32 mmol) were added and the reaction was stirred for 5 h. at 0 °C. The mixture was diluted with dichloromethane (250 mL), washed with 2 M HCl (50 mL), satd. aq. $NaHCO_3$ (50 mL) and water (50 mL). The organic phase was dried over Na_2SO_4, filtered and concentrated. The residue was purified by chromatography on silica gel (toluene/ethyl acetate, 18:1) to give **19** (955 mg, 82 %) as a syrup. R_f = 0.34 (toluene/ethyl acetate, 10:1); $[\alpha]_{20}^{D}$ = 10 (*c* = 1.2 in chloroform); ^{1}H NMR (600 MHz, $CDCl_3$, TMS): *δ* = 7.23 (ad, *J* = 8.6 Hz, 2 H, m-CH-Ar), 6.85 (ad, 2 H, o-CH-Ar), 5.97-5.88 (m, 2 H, 2 x =CH Alloc), 5.88-5.81 (m, 2 H, =CH Alloc, NH), 5.37-5.32 (m, 2 H, 2 x =CH_2 trans Alloc), 5.31 (dd, $J_{2,3}$ = 10.7 Hz, $J_{4,3}$ = 9.3 Hz, 1 H, H-3), 5.31-5.27 (m, 1 H, =CH_2 trans Alloc), 5.27-5.24 (m, 2 H, 2 x =CH_2 cis Alloc), 5.24-5.21 (m, 1 H, =CH_2 cis Alloc), 5.02–4.93 (m, 2 H, 2 x β-CH^{acyl}), 4.96 (d, $J_{2,1}$ = 7.8 Hz, 1 H, H-1), 4.86 (dd, $J_{5,4}$ = 9.9 Hz, $J_{3,4}$ = 9.3 Hz, 1 H, H-4), 4.66-4.50 (m, 6 H, OCH_2 Alloc), 4.49 (d, J_{gem} = 11.6 Hz, 1 H, -OCH_{2a} PMB), 4.44 (d, 1 H, OCH_{2b} PMB), 3.80 (s, 3 H, OMe), 3.71-3.66 (m, 2 H, H-5, H-2), 3.60-3.55 (m, $J_{6a,b}$ = 11.1 Hz, 2 H, H-6), 2.48 (d, *J* = 6.1 Hz, 2 H, α-CH_2^{acyl}), 2.48 (dd, J_{gem} = 15.2 Hz, J_{vic} = 6.5 Hz, 1 H, α-CH_{2a}^{acyl}), 2.35 (dd, J_{vic} = 5.7 Hz, 1 H, α-CH_{2b}^{acyl}), 1.68-1.55 (m, 4 H, γ-CH_2^{acyl}), 1.37-1.20 (m, 44 H, -CH_2^{acyl}), 0.89-0.86 (m, 15 H, t-Bu, 2 x ω-CH_3^{acyl}), 0.11, 0.08 (2 s, 6 H, 2 x Si-Me); ^{13}C NMR (151 MHz, $CDCl_3$): *δ* = 169.94 (CO^{acyl}), 169.33 ($CONH,^{acyl}$), 159.33 (C_q PMB), 154.79, 154.51, 154.10 (3 x C_q Alloc), 131.86, 131.74, 131.56 (=CH Alloc), 130.22 (p-C_q PMB), 129.34 (m-CH PMB), 119.15, 119.05, 118.91 (=CH_2 Alloc), 113.89 (o-CH PMB), 95.83 (C-1), 75.49, 74.68 (2 x β-CH^{acyl}), 73.69 (C-4), 73.31 (OCH_2 PMB), 73.04 (C-5), 72.68 (C-3), 69.08 (C-6), 68.98, 68.60

(OCH$_2$ Alloc), 57.36 (C-2), 55.41 (OMe), 41.50, 38.74 (2 x α-CH$_2^{acyl}$), 34.27, 34.00 (2 x γ-CH$_2^{acyl}$), 32.08, 29.85, 29.84, 29.81, 29.79, 29.73, 29.70, 29.68, 29.57, 29.53, 29.51 (CH$_2^{acyl}$), 25.79 (C$_q$ *t*Bu), 25.78 (CH$_3$ *t*Bu), 25.28, 22.11, 22.83 (CH$_2^{acyl}$), 14.26 (ω-CH$_3^{acyl}$), -3.99, -4.98 (Si-CH$_3$); HRMS ($^+$ESI-TOF): calcd. for $C_{62}H_{104}NO_{16}Si$ *m/z* [M+H]$^+$: 1146.7119, found 1146.7122.

Synthesis of 20

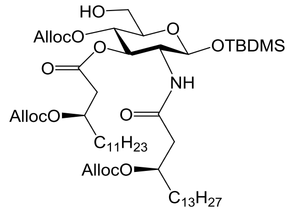

***tert*-Butyldimethylsilyl 4-*O*-allyloxycarbonyl-2-deoxy-2-[(*R*)-3-(allyloxycarbonyloxy)hexadecanoylamino]-3-O-[(*R*)-3-(allyloxycarbonyloxy)tetradecanoyl]-β-D-glucopyranoside (20)**

19 (552 mg, 0.48 mmol) was dissolved in dry dichloromethane (30 mL) and trifluoromethansulfonic acid (TFA) (3.34 mL 43.33 mmol) was added. After the reaction was stirred for 15 minutes at r.t., the mixture was diluted with dichloromethane (150 mL) and washed with sat. NaHCO$_3$ (2 x 50 mL). The organic layer was dried over Na$_2$SO$_4$, filtered and concentrated, the residue was re-dissolved in toluene and concentrated to dryness (2 x 50 mL) to give **20** (456 mg, 92 %) a a colorless syrup. R_f = 0.48 (dichloromethane/ethyl acetate 6:1); $[\alpha]_{20}^{D}$ = +7.5 (*c* = 0.9 in chloroform); ^{1}H NMR (600 MHz, CDCl$_3$, TMS): *δ* = 5.97-5.86 (m, 4 H, 3 x =CH Alloc, NH), 5.38 (dd, $J_{2,3}$ = 10.6 Hz, $J_{4,3}$ = 9.2 Hz, 1 H, H-3), 5.37-5.31 (m, 3 H, 3 x =CH$_2$ trans Alloc), 5.28-5.25 (m, 3 H, 3 x =CH$_2$ cis Alloc), 5.04 (d, $J_{2,1}$ = 7.9 Hz, 1 H, H-1), 5.02–4.93 (m, 2 H, 2 x β-CH^{acyl}), 4.85 (t, $J_{5,4}$ = 9.9 Hz 1 H, H-4), 4.67-4.57 (m, 6 H, OCH$_2$ Alloc), 3.76 (ddd, $J_{5,6a}$ = 2.6 Hz, $J_{6\text{-}OH,6a}$ = 7.4 Hz, $J_{6a,b}$ = 12.6 Hz, 1 H, H-6a), 3.68-3.62 (m, 2 H, H-6b, H-2), 3.57 (ddd, $J_{6b,5}$ = 4.6 Hz, 1H, H-5), 2.59 (d, *J* = 6.1 Hz, 2 H, α-CH$_2^{acyl}$), 2.49 (dd, J_{gem} = 15.2 Hz, J_{vic} = 6.4 Hz, 1 H, α-CH$_{2a}^{acyl}$), 2.36 (dd, J_{vic} = 5.6 Hz, 1 H, α-CH$_{2b}^{acyl}$), 2.08 (t, 1 H, 6-OH), 1.68-1.55 (m, 4 H, γ-CH$_2^{acyl}$), 1.40-1.20 (m, 44 H, CH$_2^{acyl}$), 0.90-0.85 (m, 15 H, *t*Bu, 2 x ω-CH$_3^{acyl}$), 0.11, 0.09 (2 s, 6 H, 2 x Si-Me); ^{13}C NMR (150 MHz, CDCl$_3$): *δ* = 169.85 (CO^{acyl}), 169.42 (CONH,^{acyl}), 154.79, 154.51 (C$_q$ Alloc), 131.84, 131.69, 131.33 (3 x =CH Alloc), 119.38, 119.20, 118.91 (3 x =CH$_2$ Alloc), 95.74 (C-1), 75.44, 74.70 (2 x β-CH^{acyl}), 73.89 (C-5), 72.88 (C-4), 72.26 (C-3), 69.24, 68.64, 68.60 (3 x OCH$_2$ Alloc), 61.66 (C-6), 57.50 (C-2), 41.47, 38.76 (2 x α-CH$_2^{acyl}$), 34.28, 34.00 (2 x γ-CH$_2^{acyl}$), 32.07, 29.85, 29.83, 29.80, 29.78, 29.73, 29.69, 29.67, 29.56, 29.51 (CH$_2^{acyl}$), 25.72 (CH$_3$ *t*Bu), 25.28, 25.13, 22.82 (CH$_2^{acyl}$), 17.99 (C$_q$ *t*Bu), 14.25 (ω-CH$_3^{acyl}$), -4.00, -4.90 (Si-CH$_3$); HRMS ($^+$ESI-TOF): calcd. for $C_{54}H_{96}NO_{15}Si$ *m/z* [M+H]$^+$: 1026.6544, found 1026.6568.

References

[1] Bazin-Lee, H. G., Johnson, C. L., Johnson, D. A., and Sowell, C. G. **2005,** Processes for the production of aminoalkyl glucosaminide phosphate and disaccharide immunoeffectors, and intermediates therefor. Patent: EP20030763418 - [PCT/US2003/021504].

[2] K. M. Koeller, C. H. Wong, *Chem.Eur.J.* **2000,** *6*, 1243-1251.

[3] D. S. Keegan, S. R. Hagen, D. A. Johnson, *Tetrahedron: Asymmetry* **1996,** *7*, 3559-3564.

[4] Y. Oikawa, K. Sugano, O. Yonemitsu, *J.Org.Chem.* **1978,** *43*, 2087-2088.

[5] R. Noyori, T. Ohkuma, M. Kitamura, H. Takaya, N. Sayo, H. Kumobayashi, S. Akutagawa, *J.Am.Chem.Soc.* **1987,** *109*, 5856-5858.

[6] S. A. King, A. S. Thompson, A. O. King, T. R. Verhoeven, *J.Org.Chem.* **1992,** *57*, 6689-6691.

[7] R. Kramer, R. Brückner, *Angew.Chem.Int.Ed.* **2007,** *46*, 6537-6541.

NMR and Mass- spectra

^{1}H and ^{13}C NMR spectra; ESI-MS spectra of compounds 1 and 2

5, ^{1}H NMR, 600 MHz, $CDCl_3$

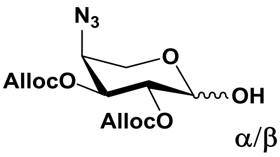

α/β = 2:1

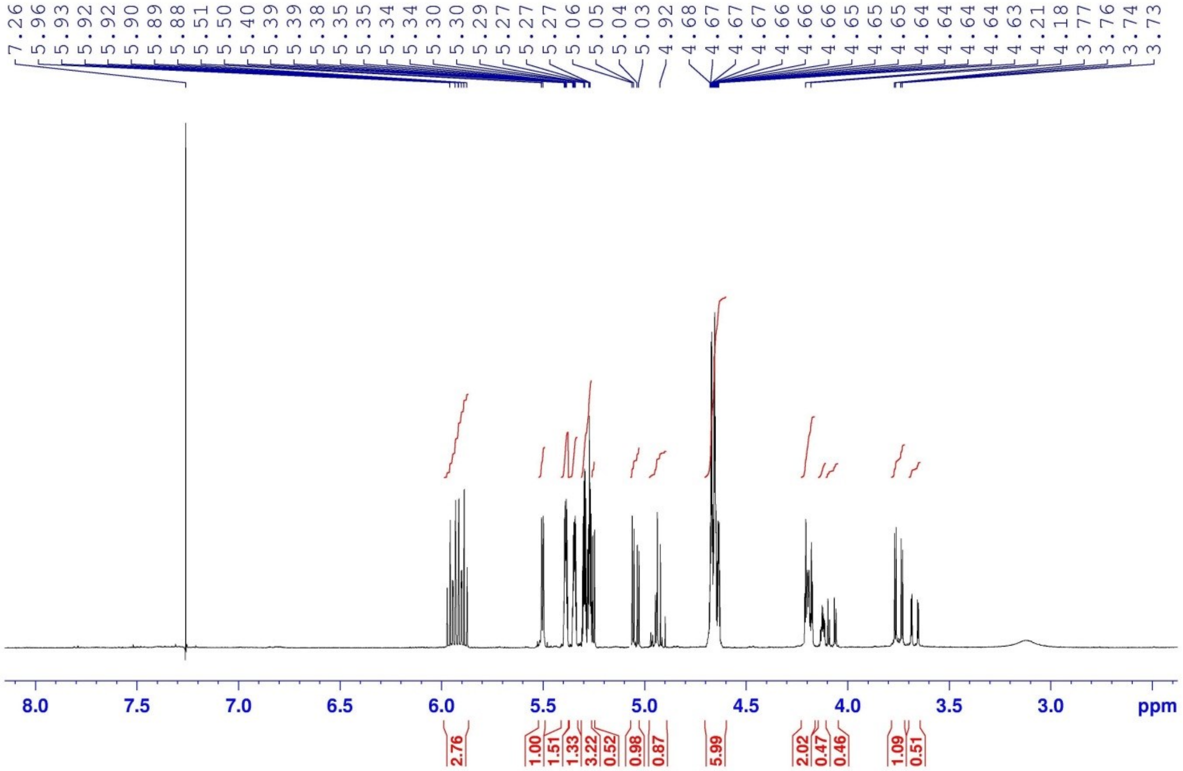

5, ^{13}C NMR, 151 MHz, $CDCl_3$

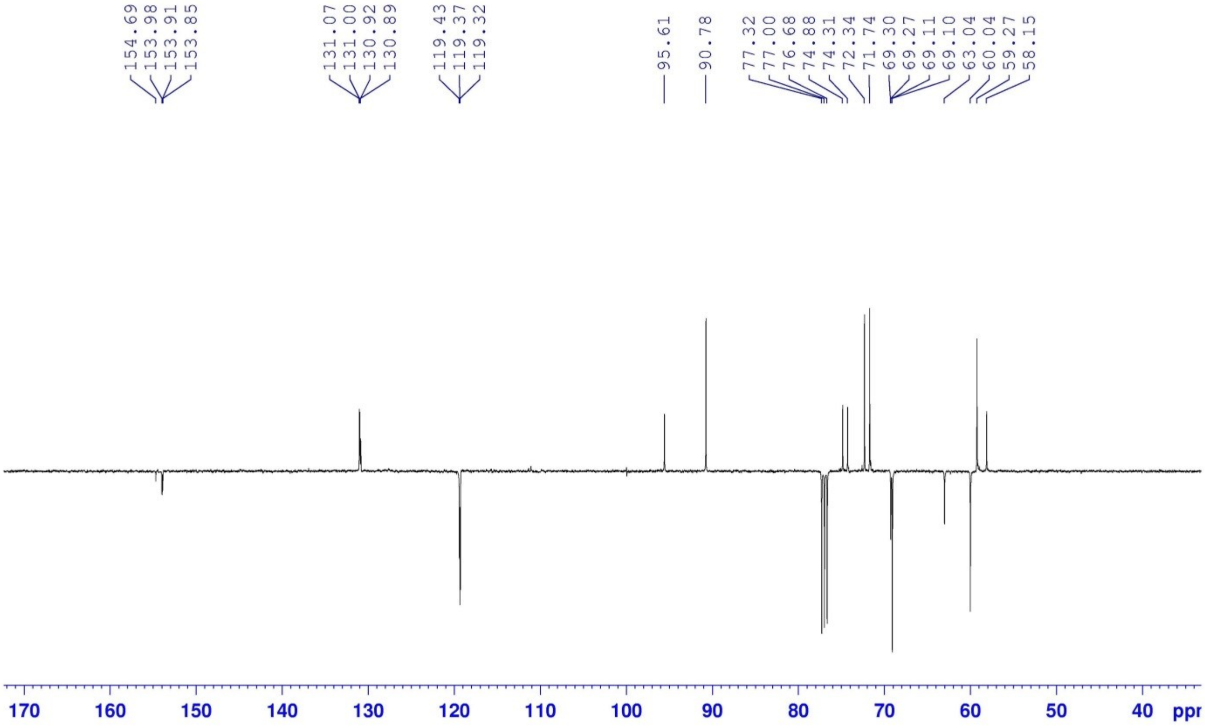

6, ^{1}H NMR, 600 MHz, $CDCl_3$

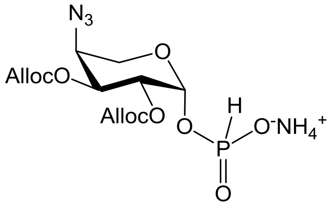

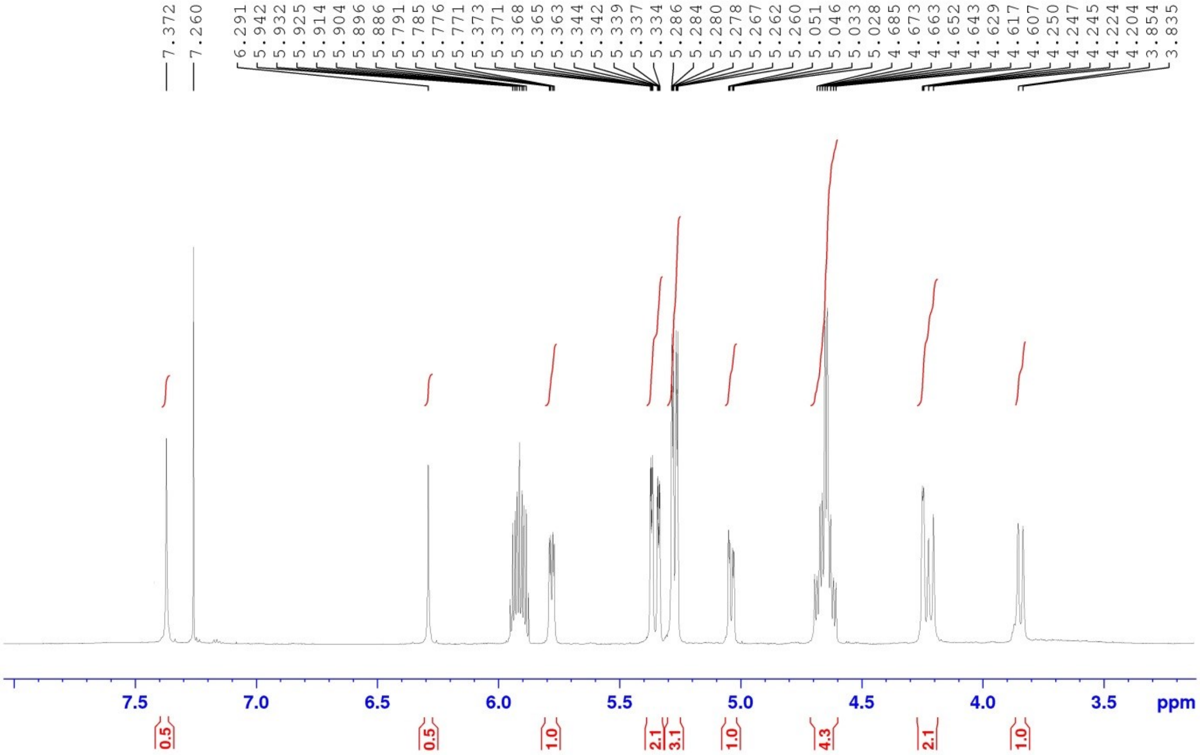

6, ^{13}C NMR, 151 MHz, $CDCl_3$

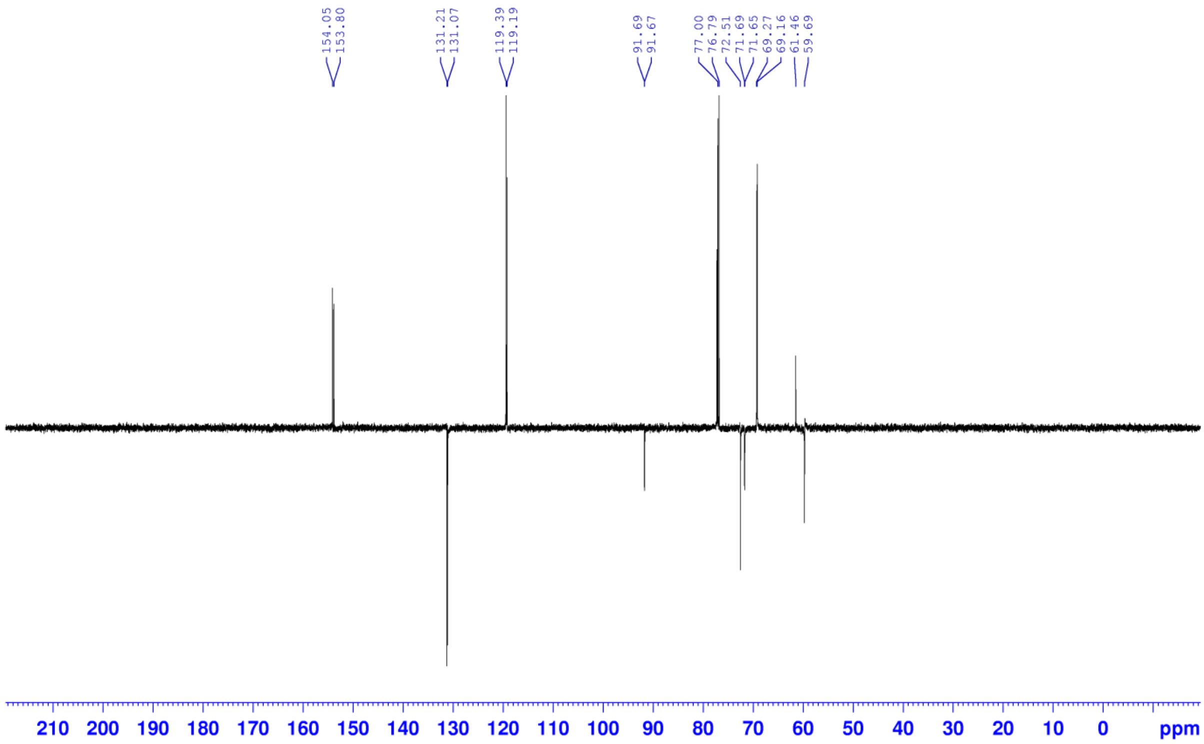

6, ^{31}P NMR, 243 MHz, $CDCl_3$

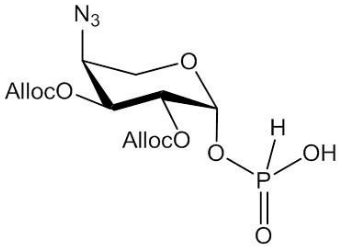

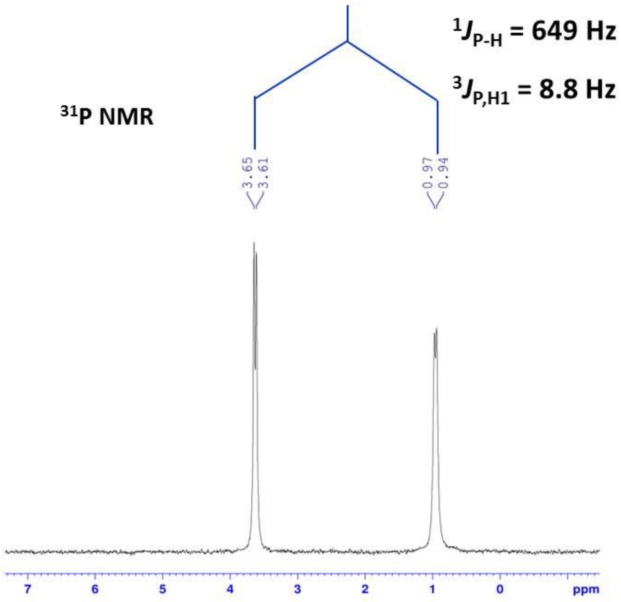

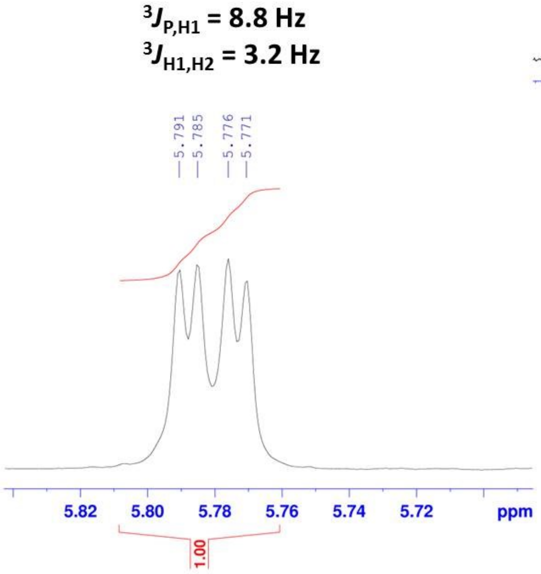

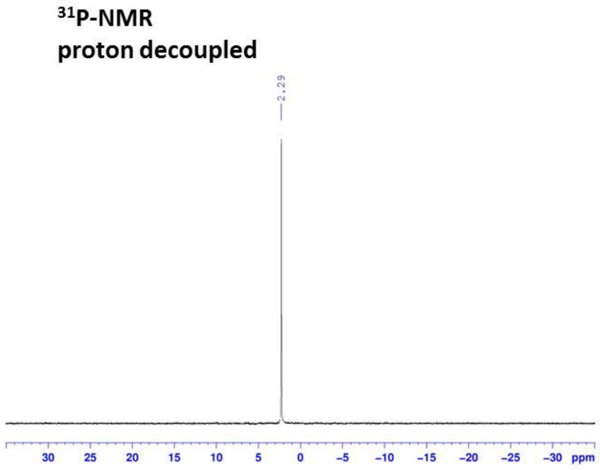

8, ^{1}H NMR, 600 MHz, $CDCl_3$

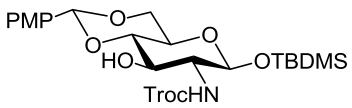

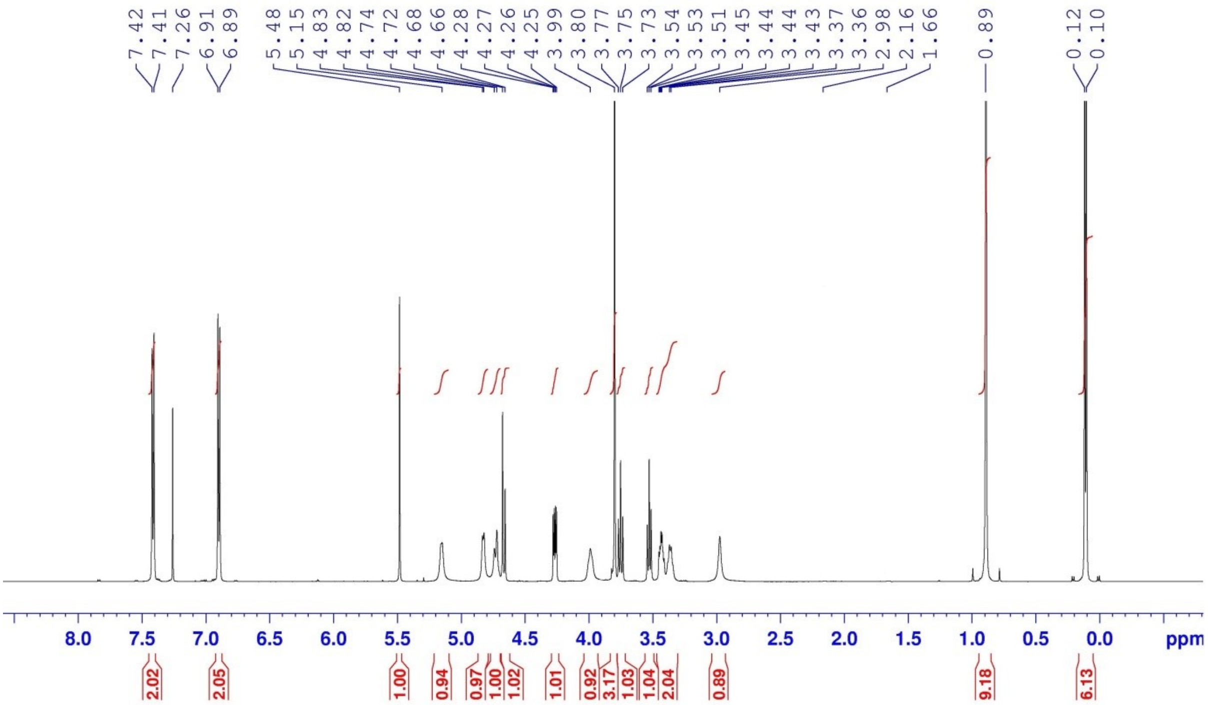

8, ^{13}C NMR, 150 MHz, $CDCl_3$

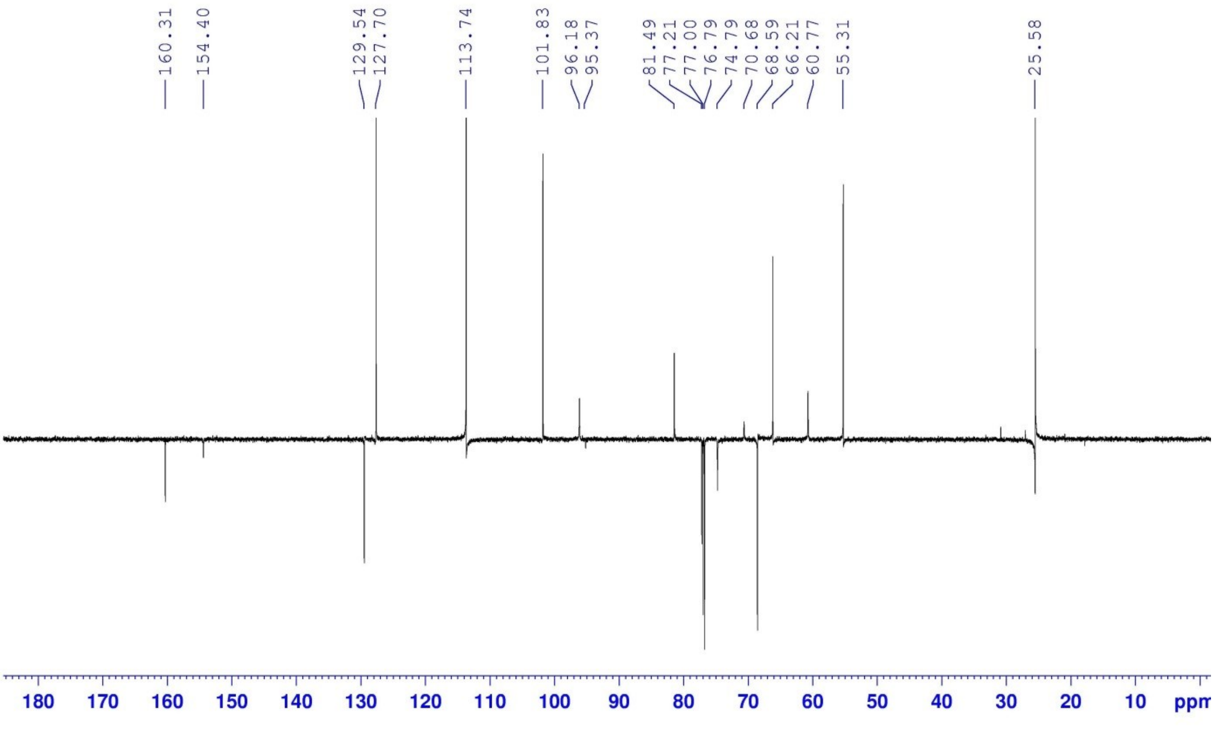

12, ^{1}H NMR, 600 MHz, $CDCl_3$

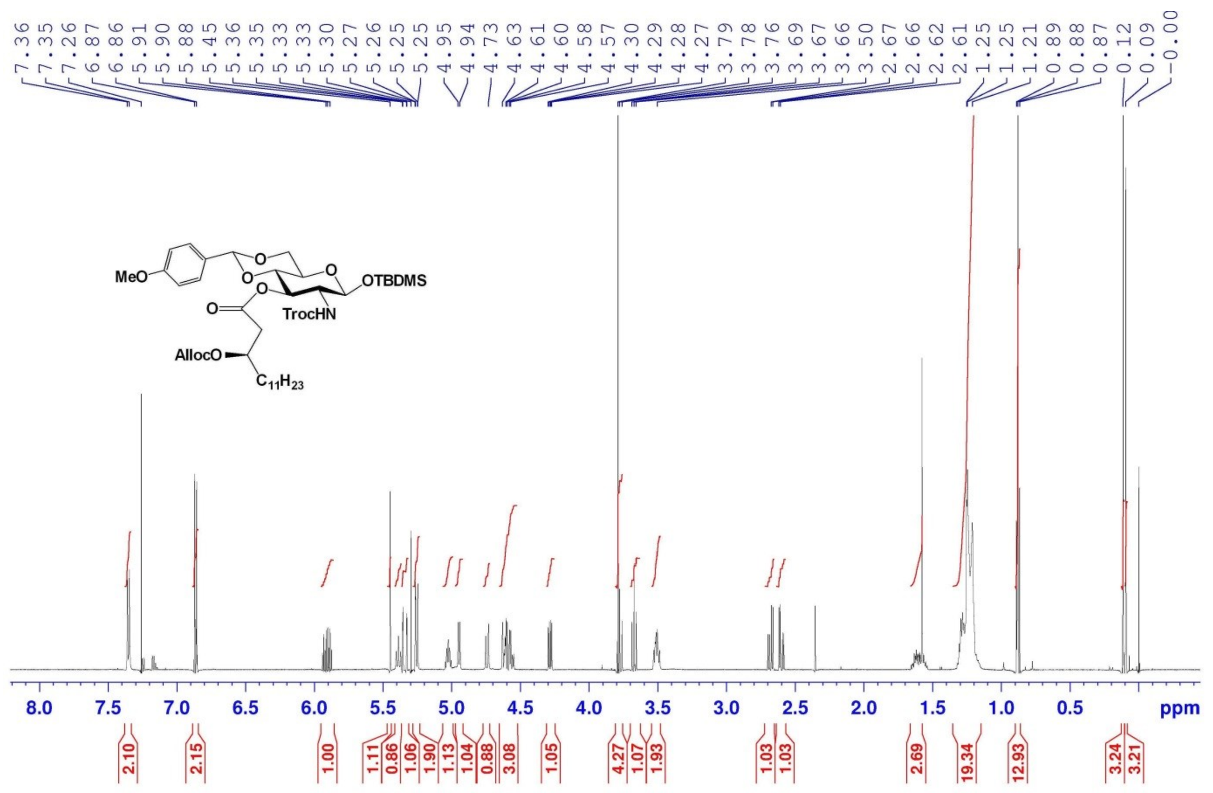

12, ^{13}C NMR, 150 MHz, $CDCl_3$

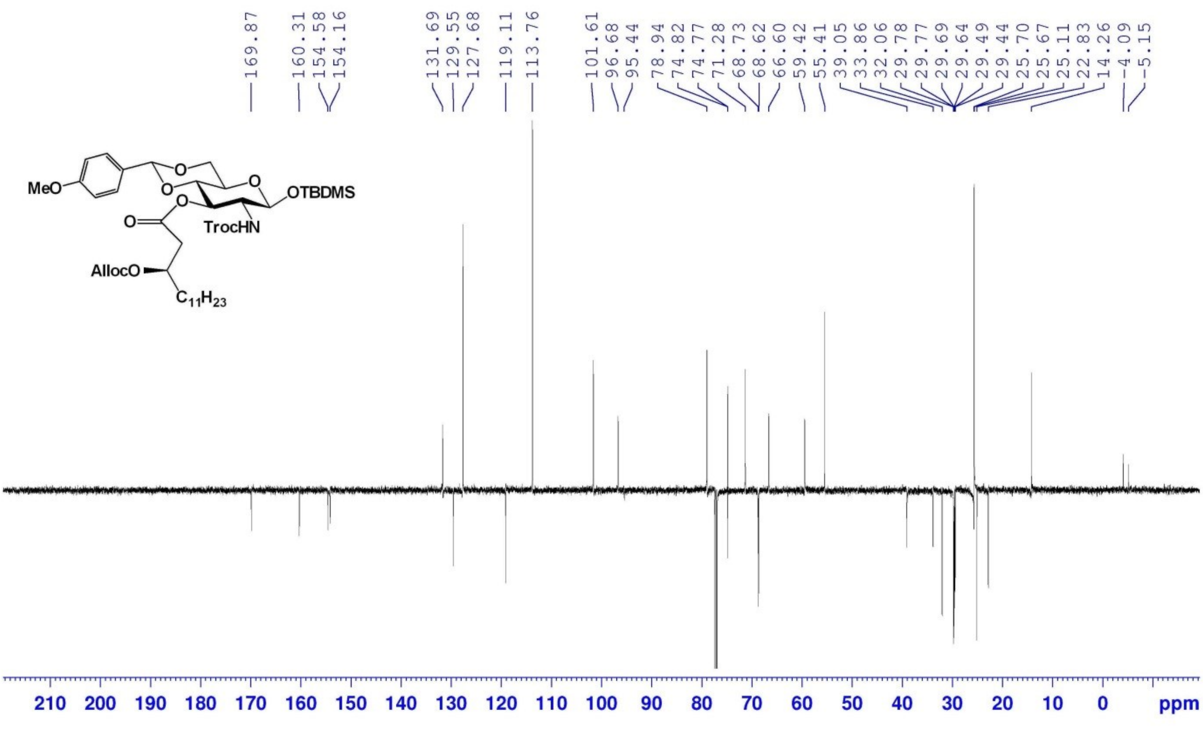

14, ^{1}H NMR, 600 MHz, $CDCl_3$

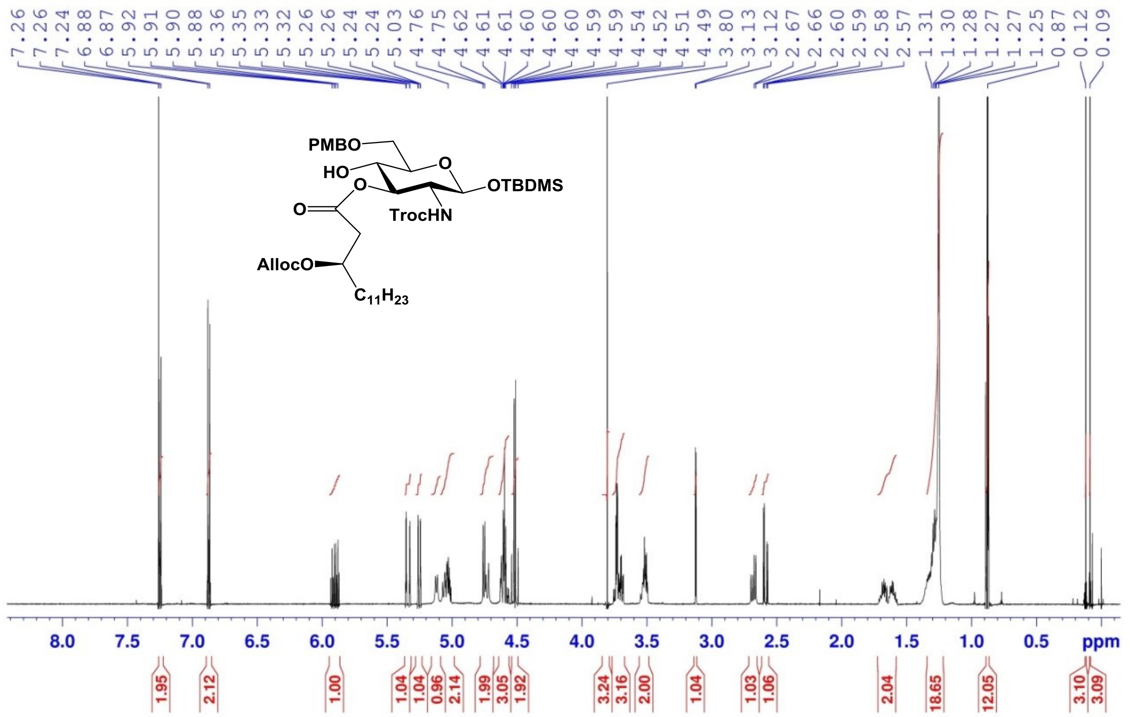

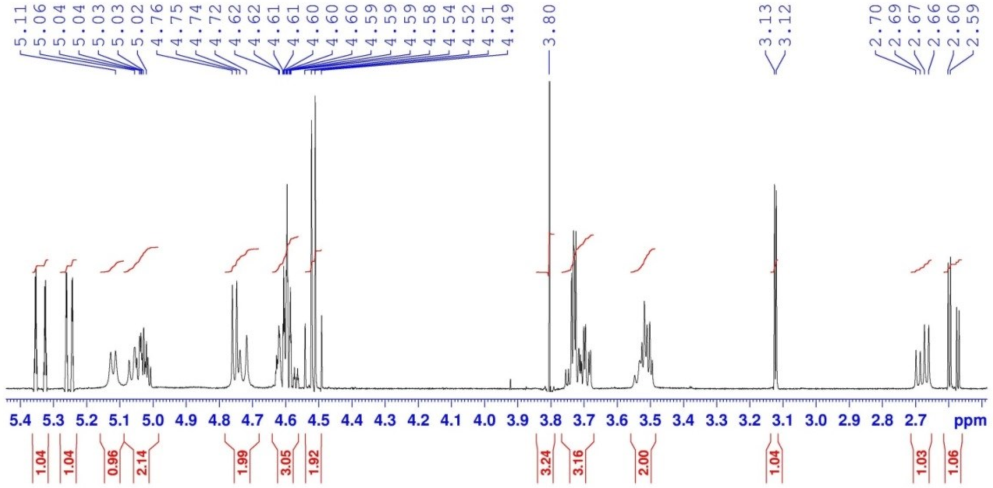

14, ^{13}C NMR, 150 MHz, $CDCl_3$

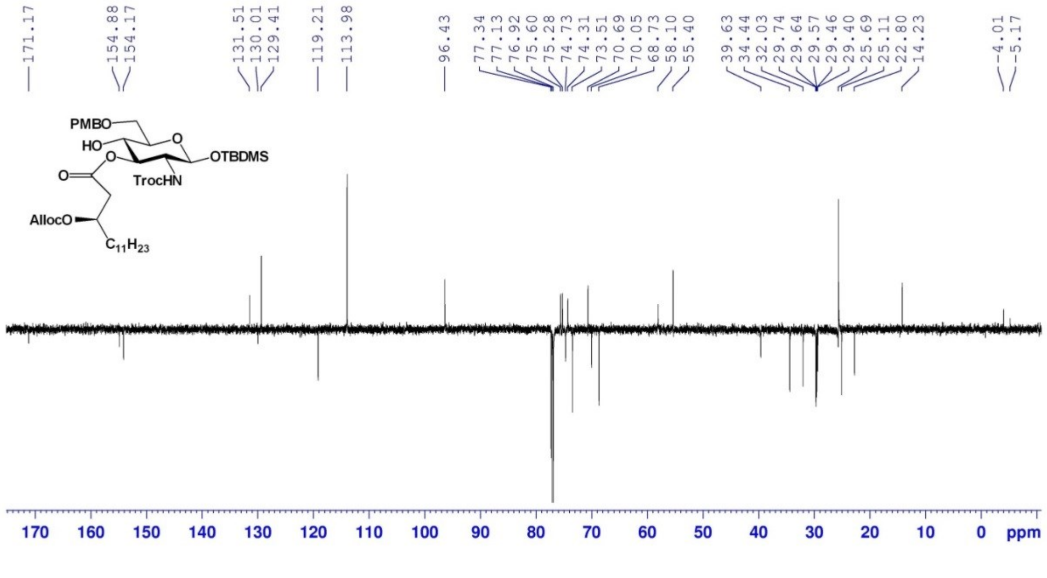

15, ^{1}H NMR, 600 MHz, $CDCl_3$

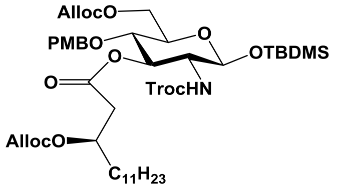

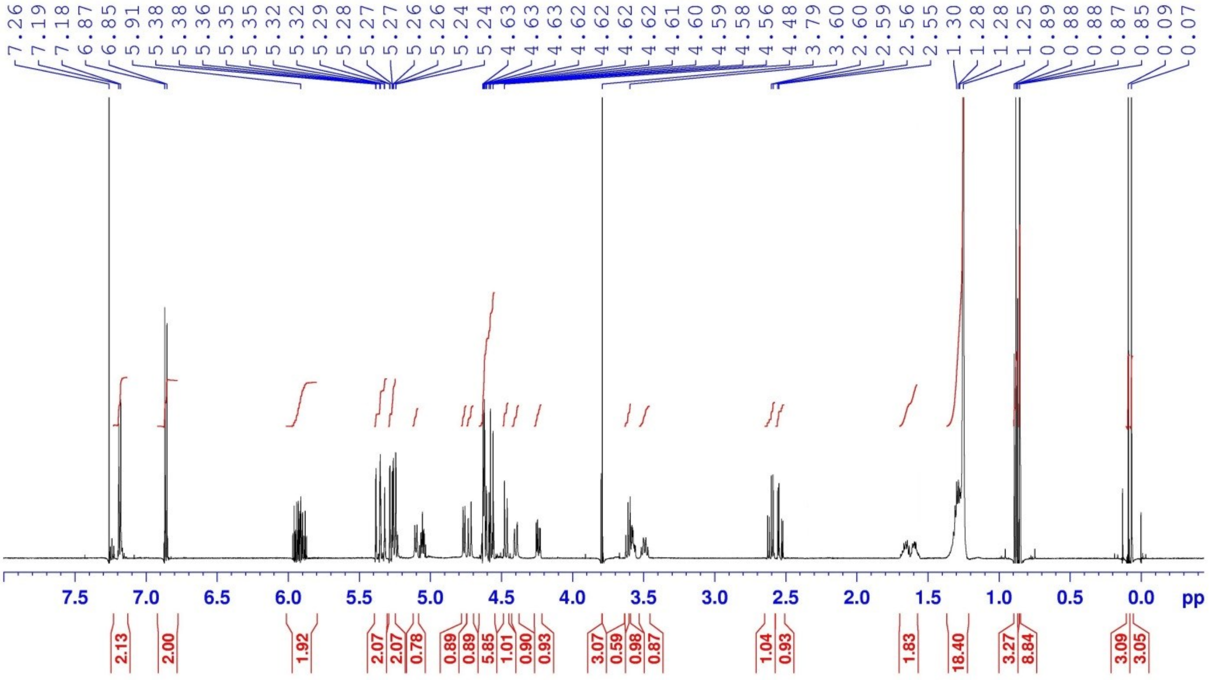

15, ^{1}H NMR, 600 MHz, $CDCl_3$, 4.0 – 6.0 ppm

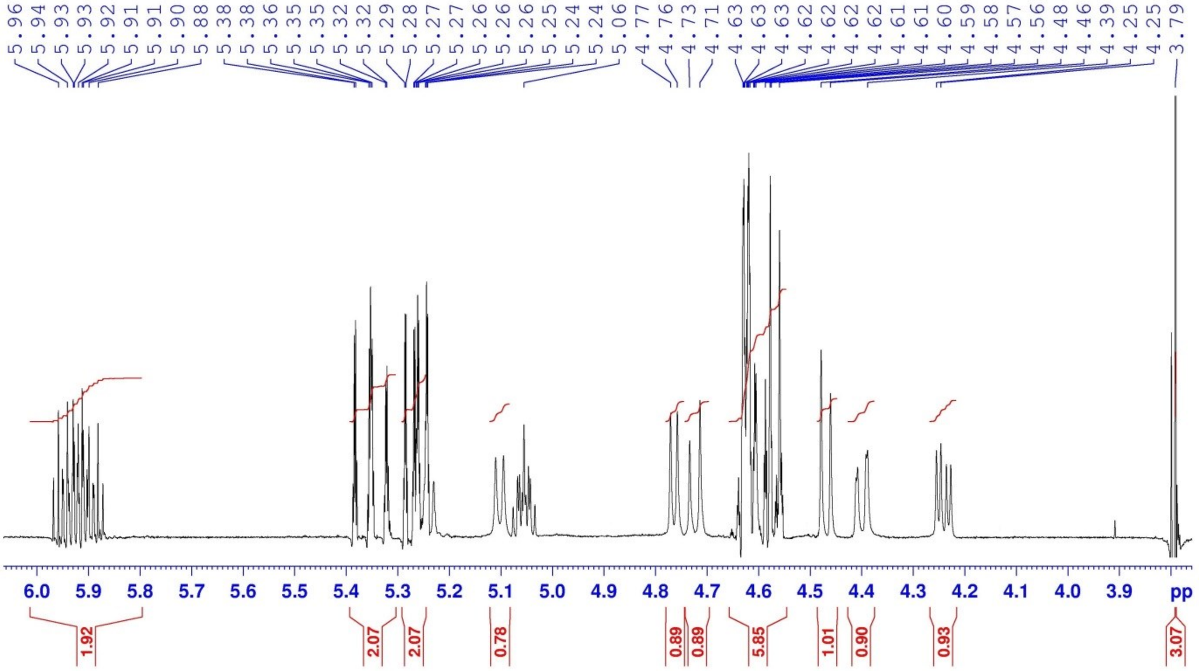

15, ^{13}C NMR, 150 MHz, $CDCl_3$

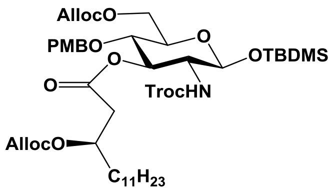

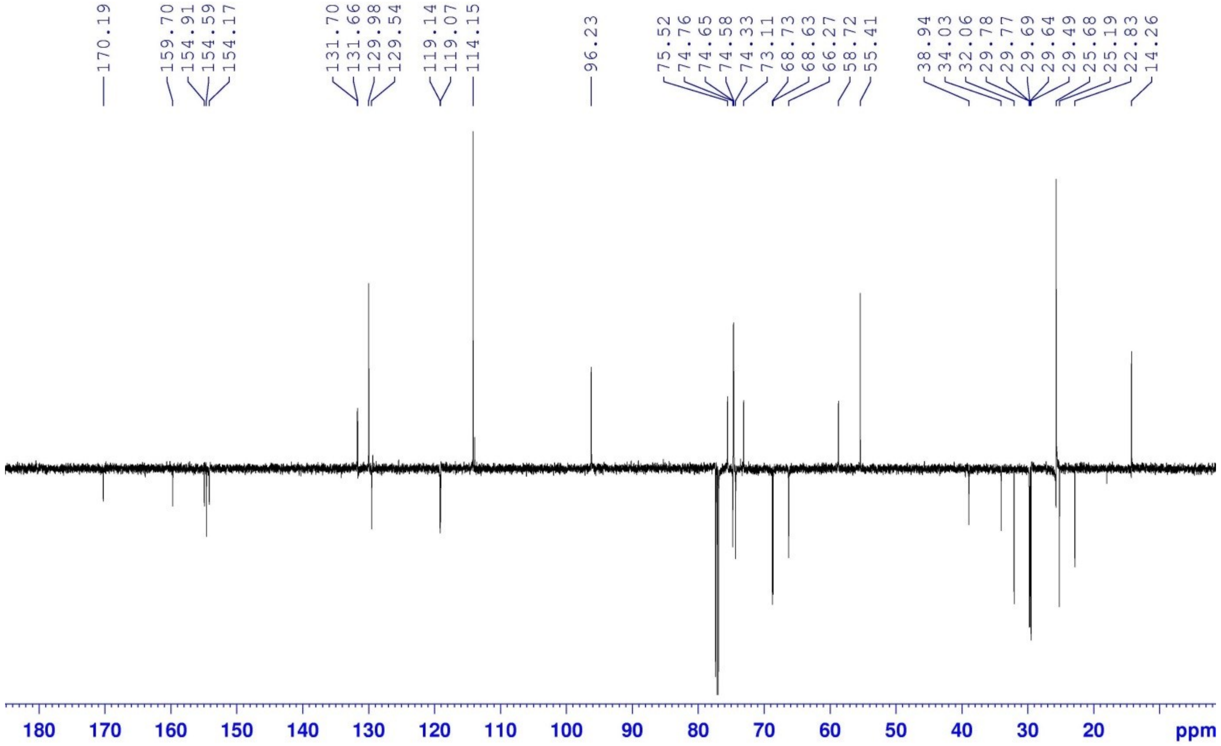

15, ^{13}C NMR, 150 MHz, $CDCl_3$, 50-100 ppm

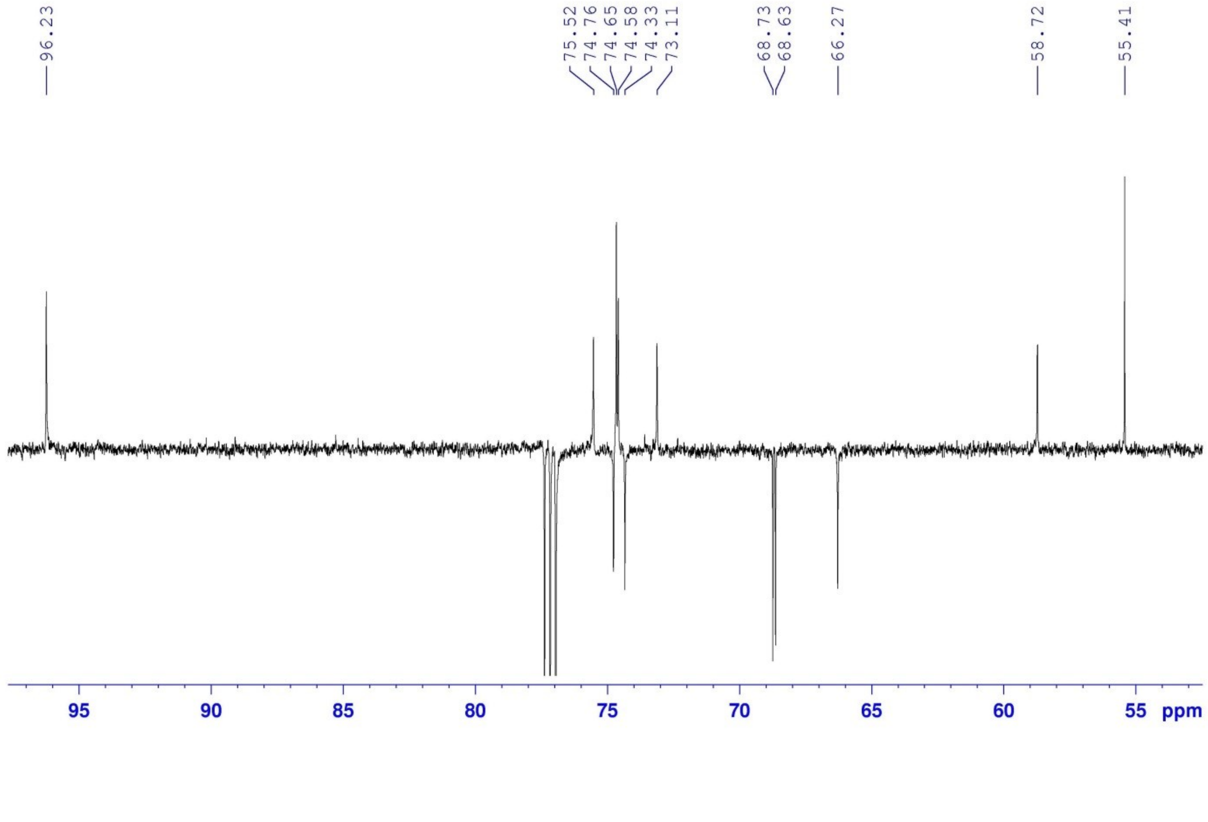

16, α/β = 10:1, ^{1}H NMR, 600 MHz, $CDCl_3$

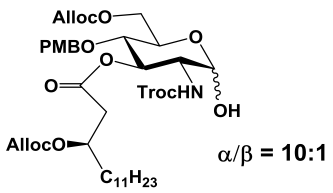

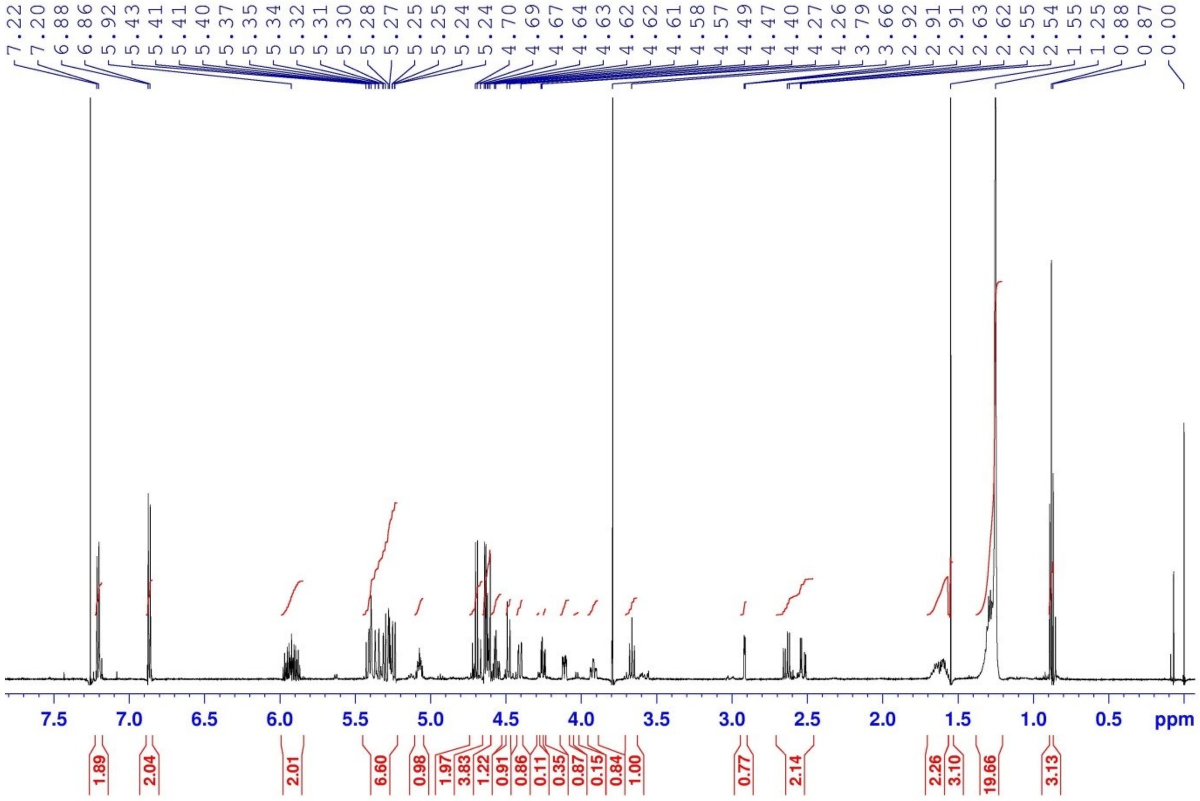

16, α/β = 10:1, ^{13}C NMR, 150 MHz, $CDCl_3$

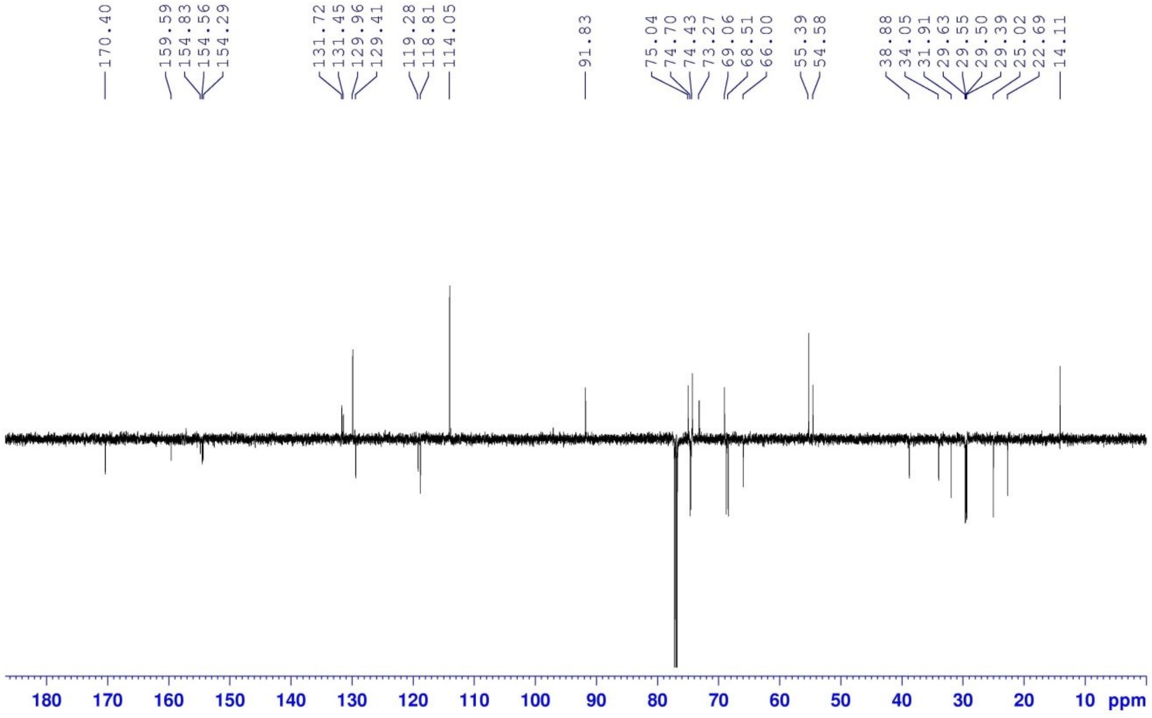

17, ^{1}H NMR, 600 MHz, $CDCl_3$

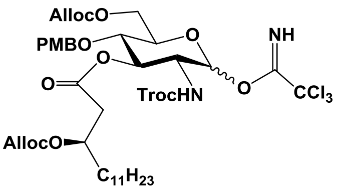

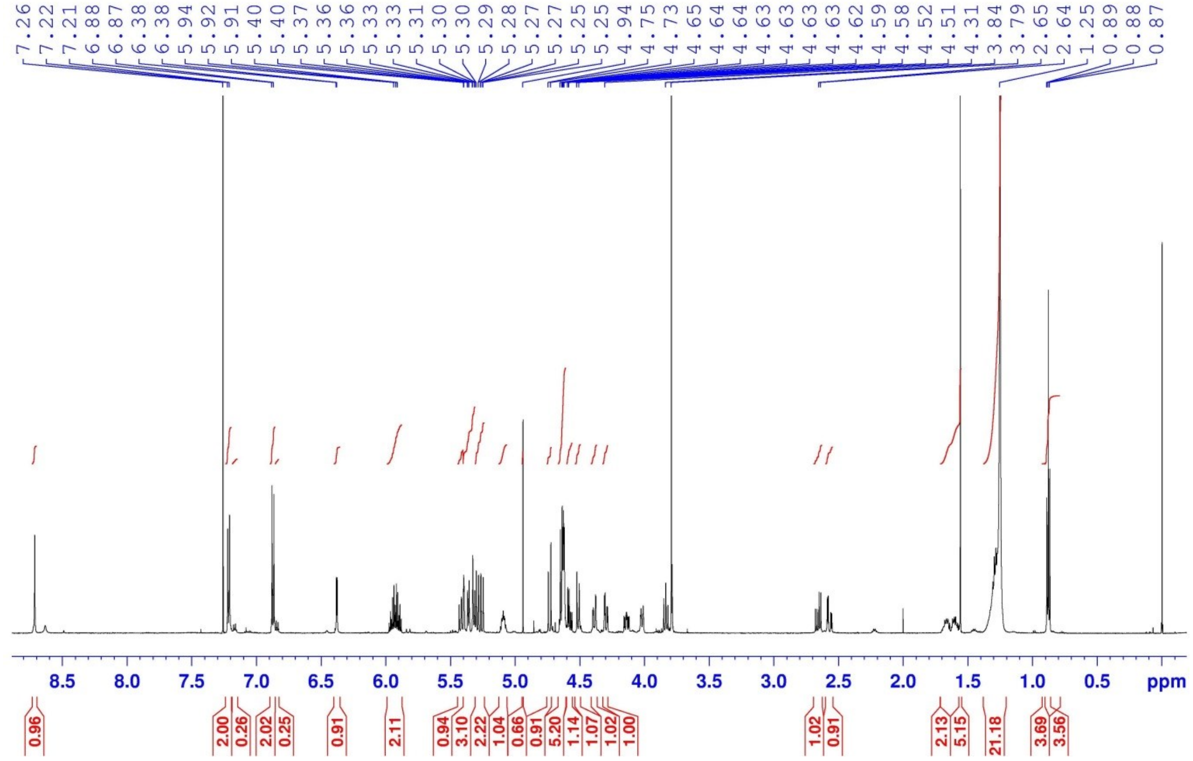

17, ^{13}C NMR, 150 MHz, $CDCl_3$

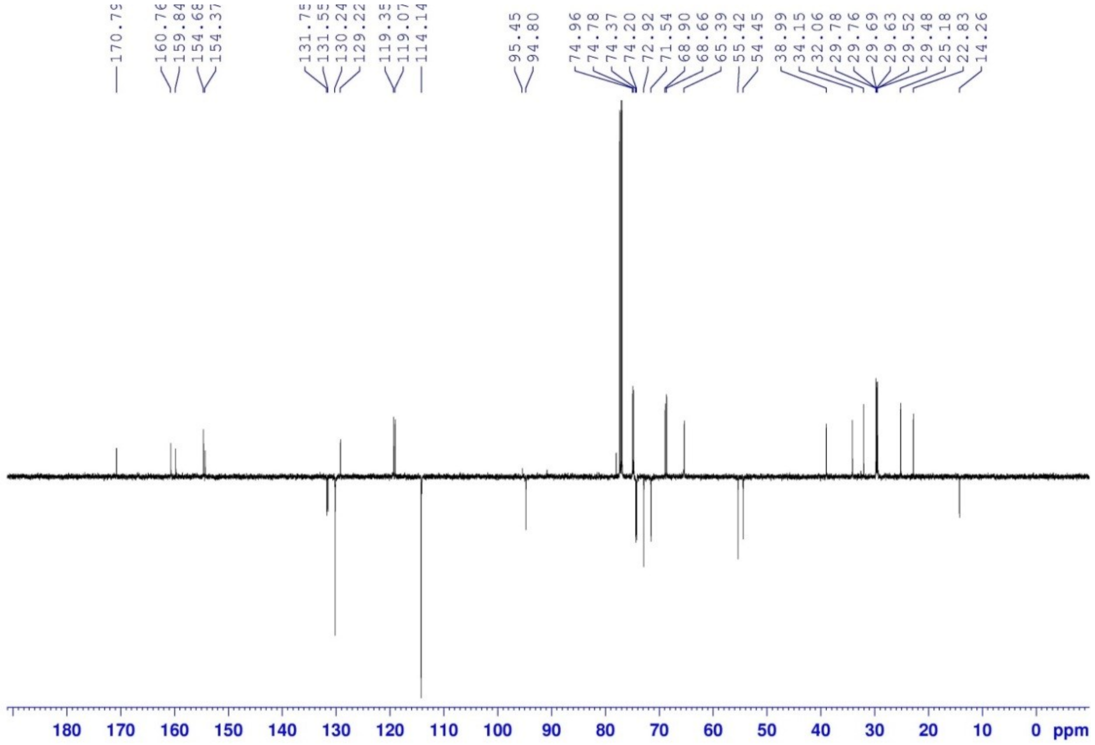

18, ^{1}H NMR, 600 MHz, $CDCl_3$

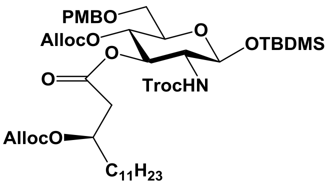

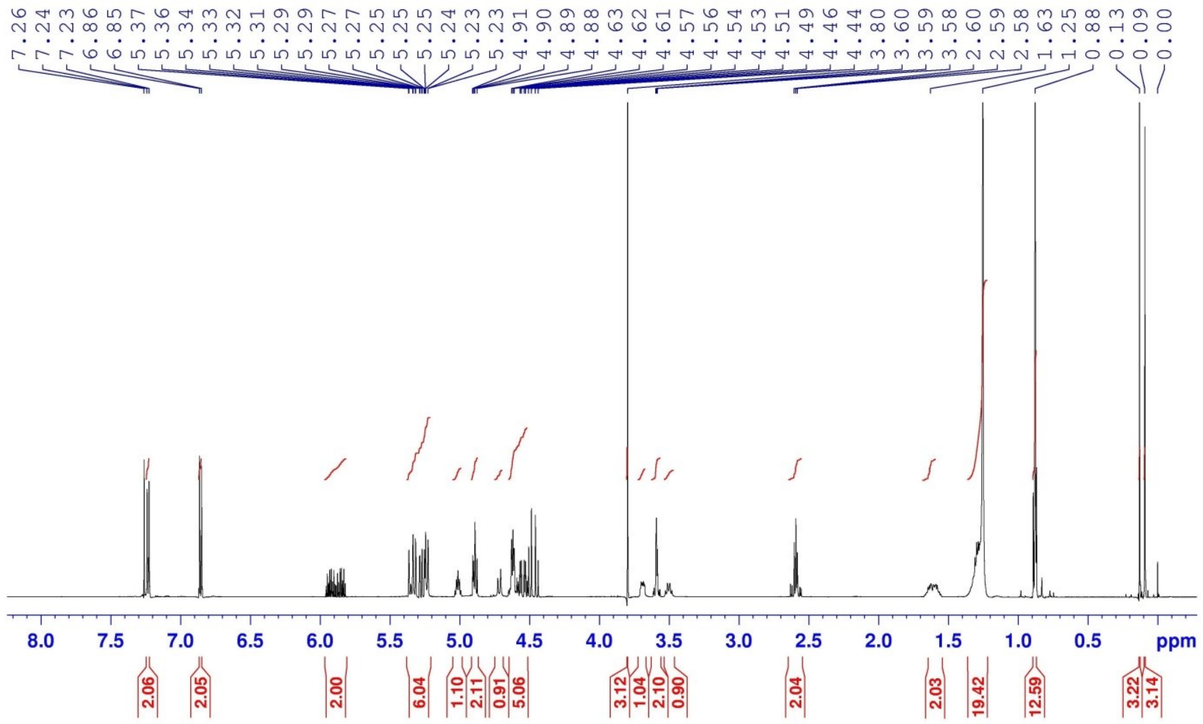

18, ^{13}C NMR, 150 MHz, $CDCl_3$

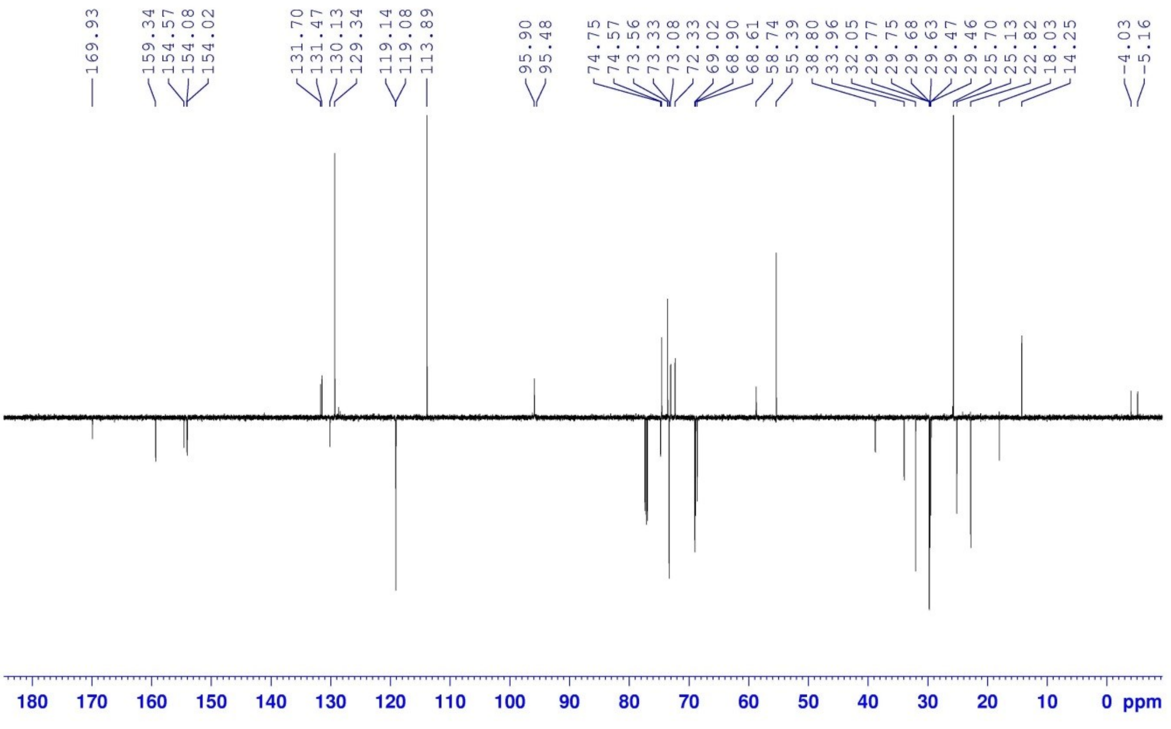

19, ^{1}H NMR, 600 MHz, $CDCl_3$

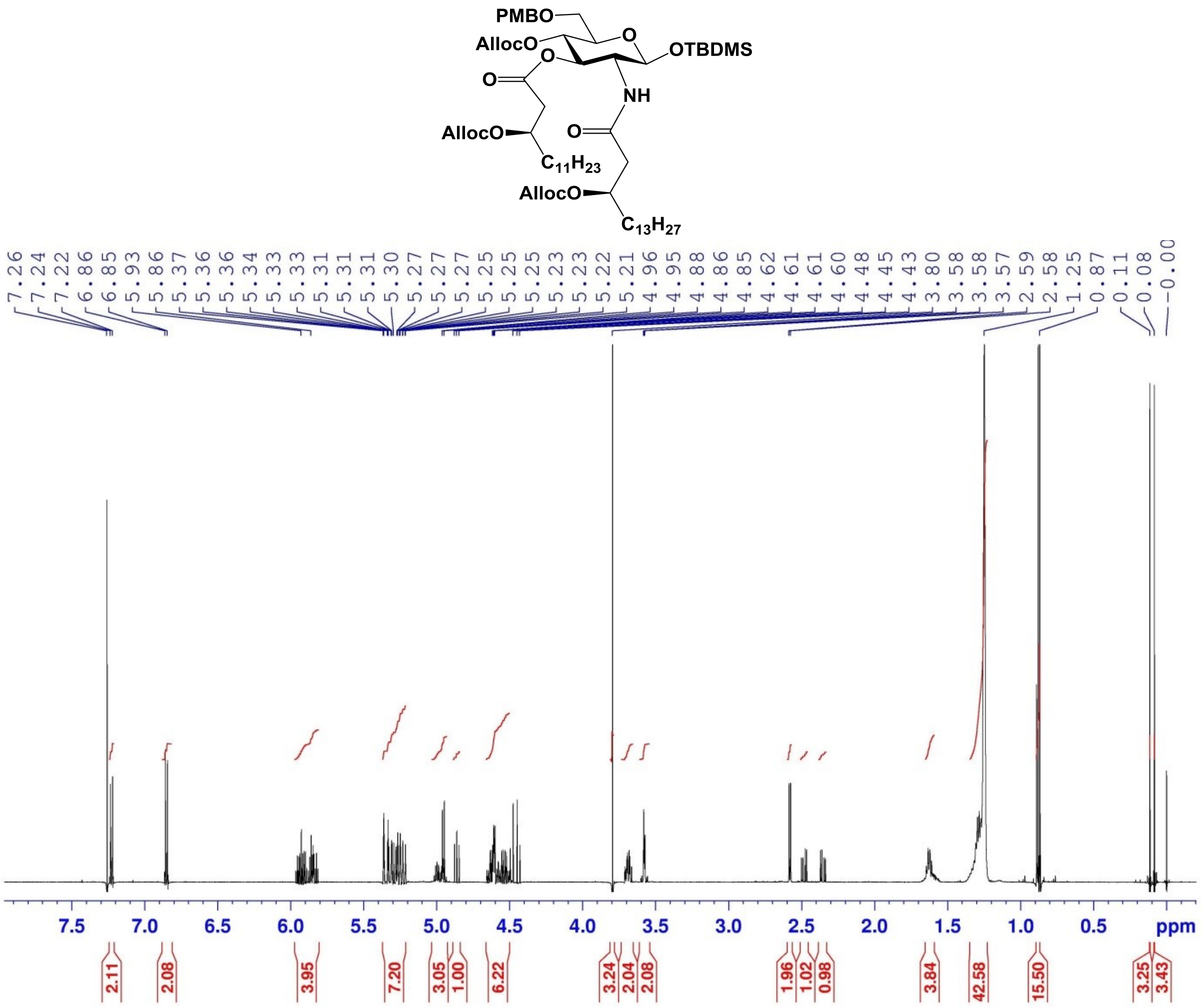

19, ^{1}H NMR, 600 MHz, $CDCl_3$

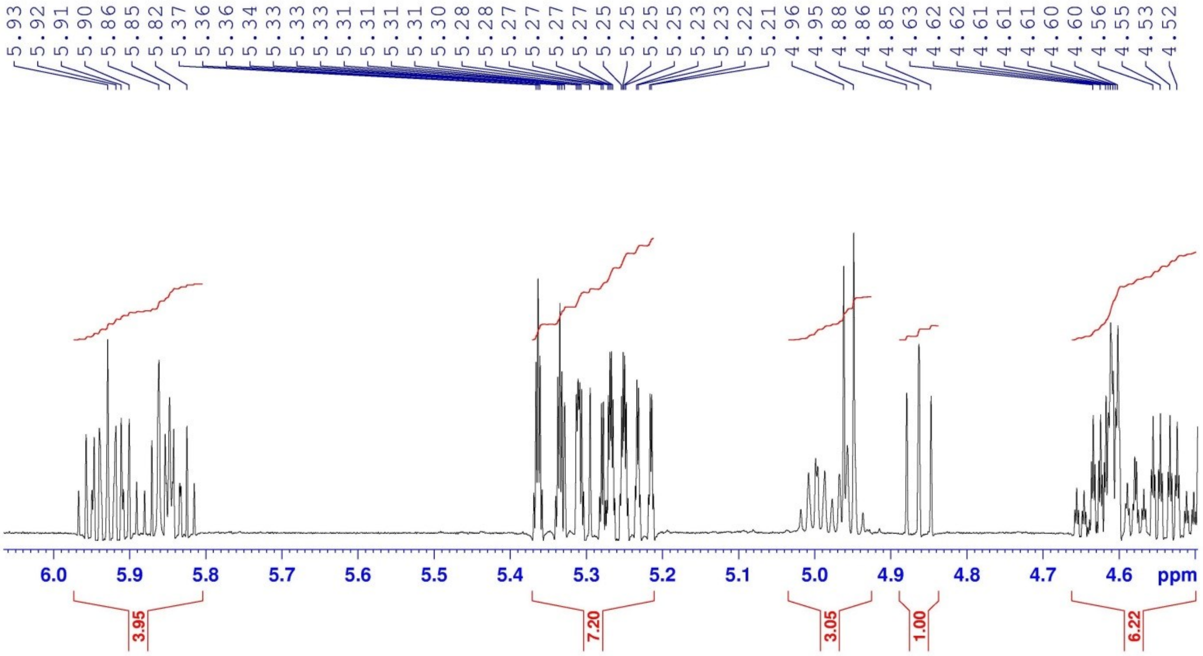

19, ^{13}C NMR, 150 MHz, CDCl$_3$

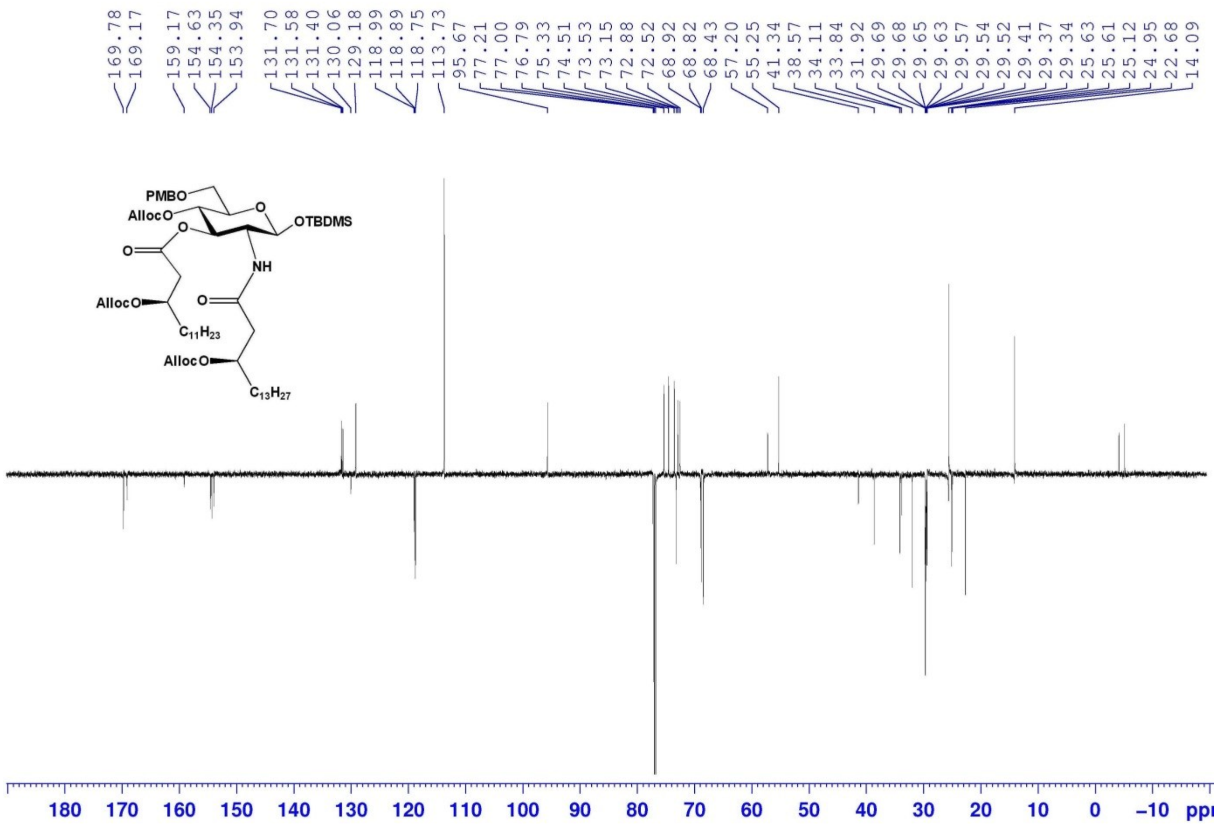

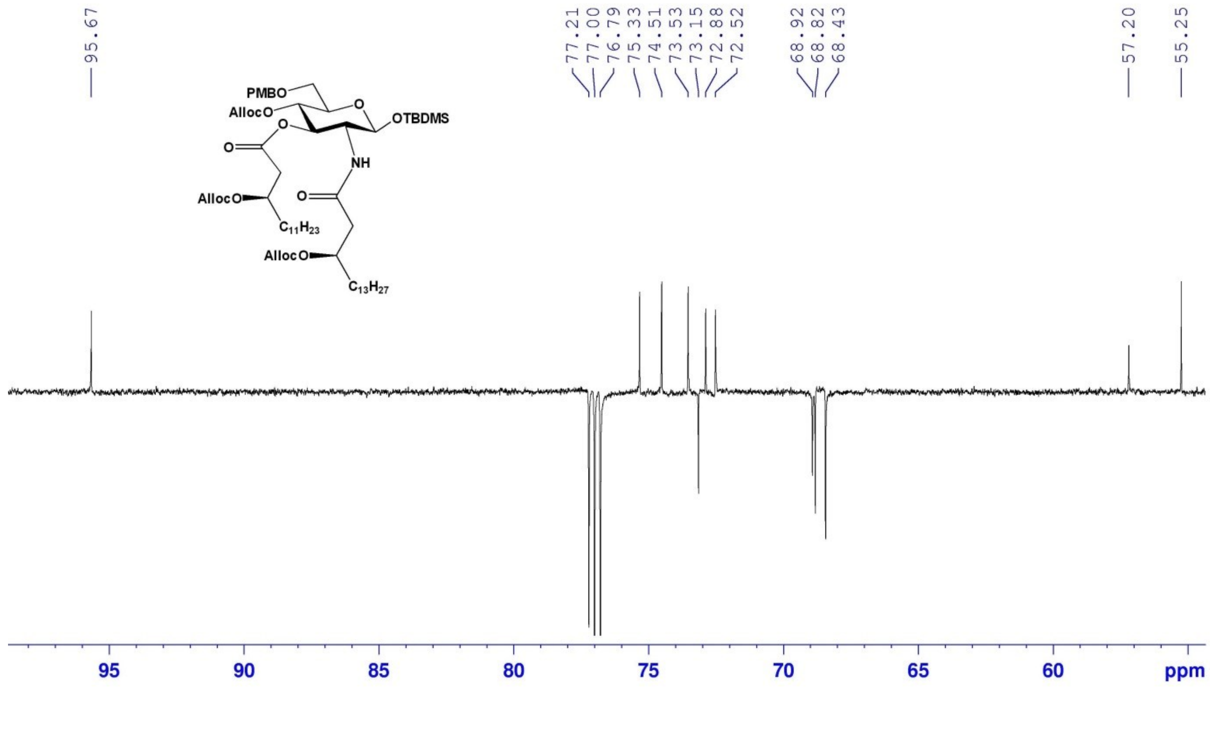

20, ^{1}H NMR, 600 MHz, $CDCl_3$

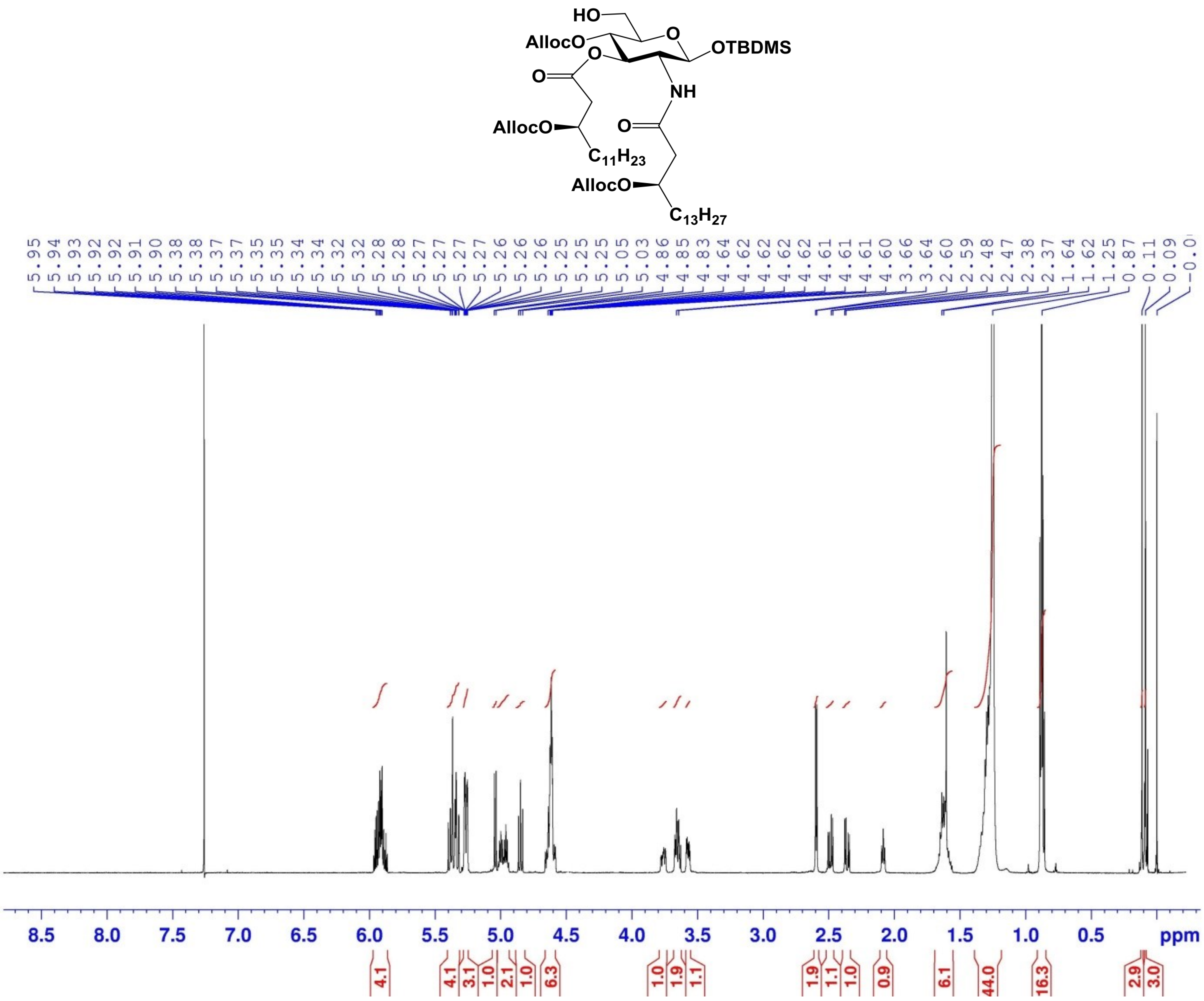

20, ^{13}C NMR, 150 MHz, $CDCl_3$

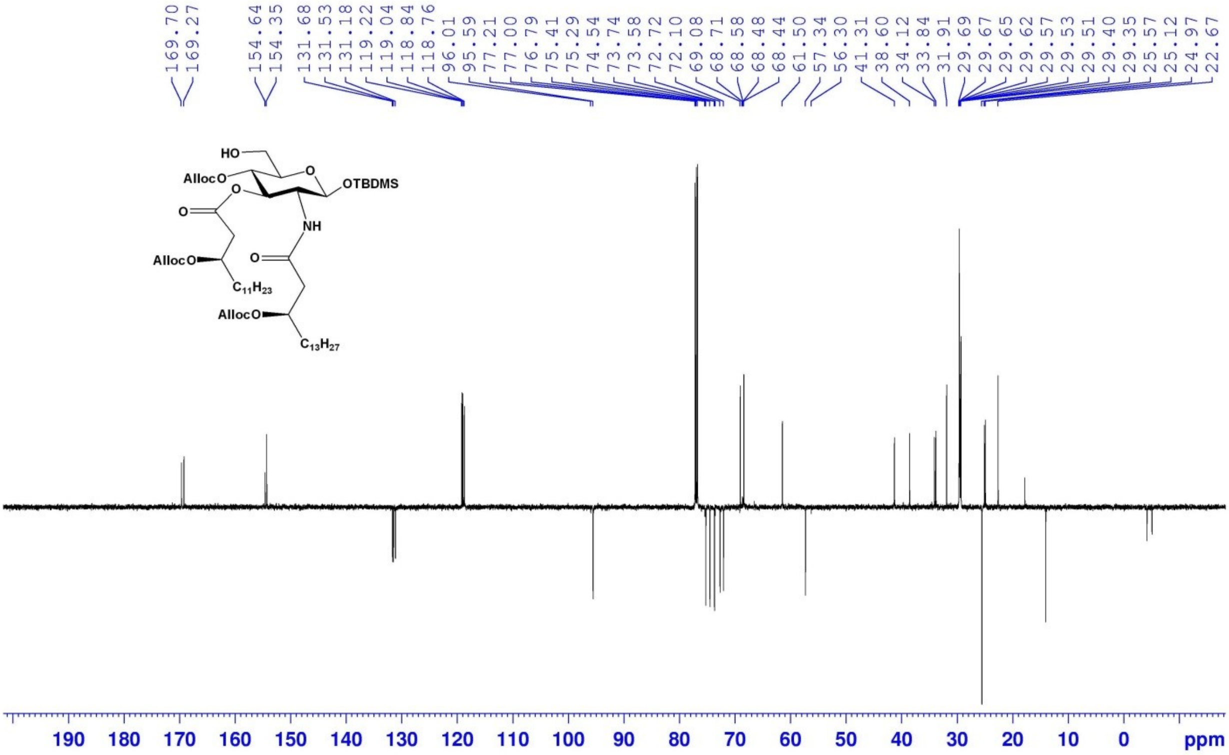

21, ^{1}H NMR, 600 MHz, $CDCl_3$

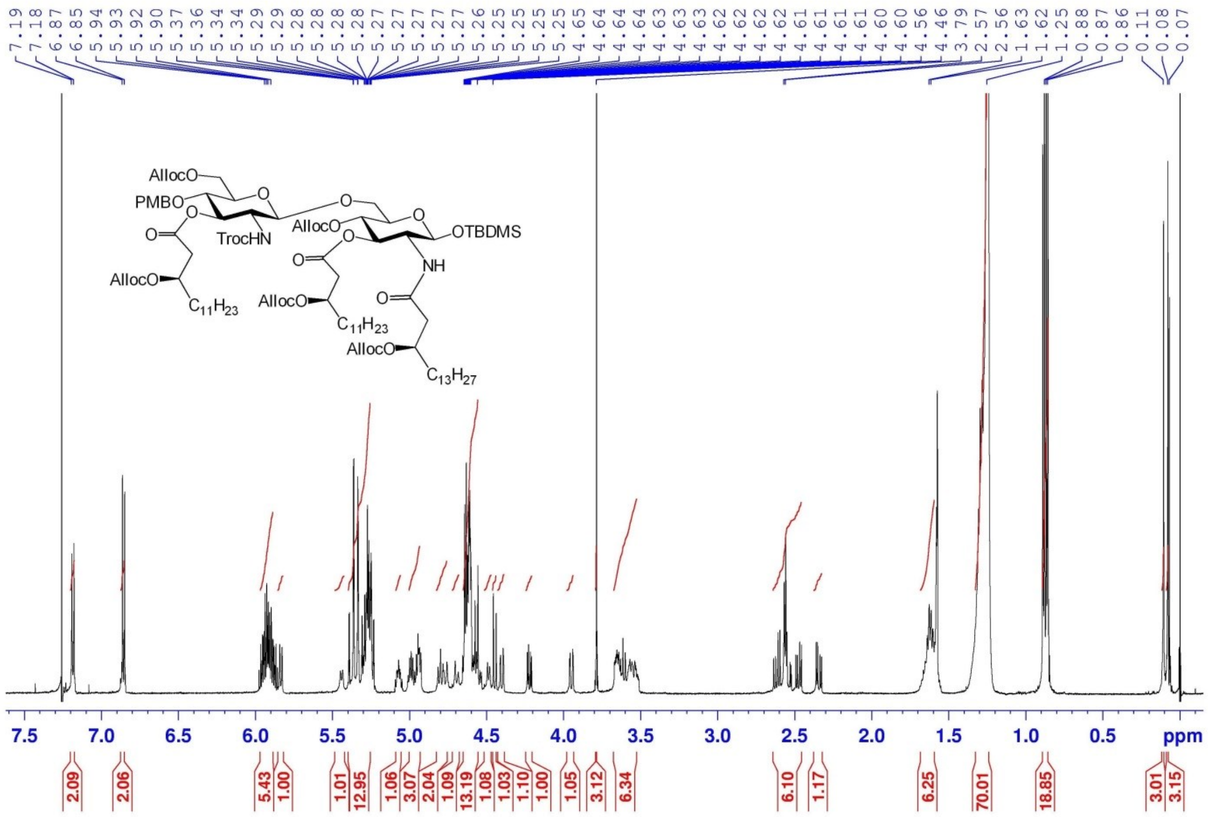

21, ^{13}C NMR, 150 MHz, $CDCl_3$

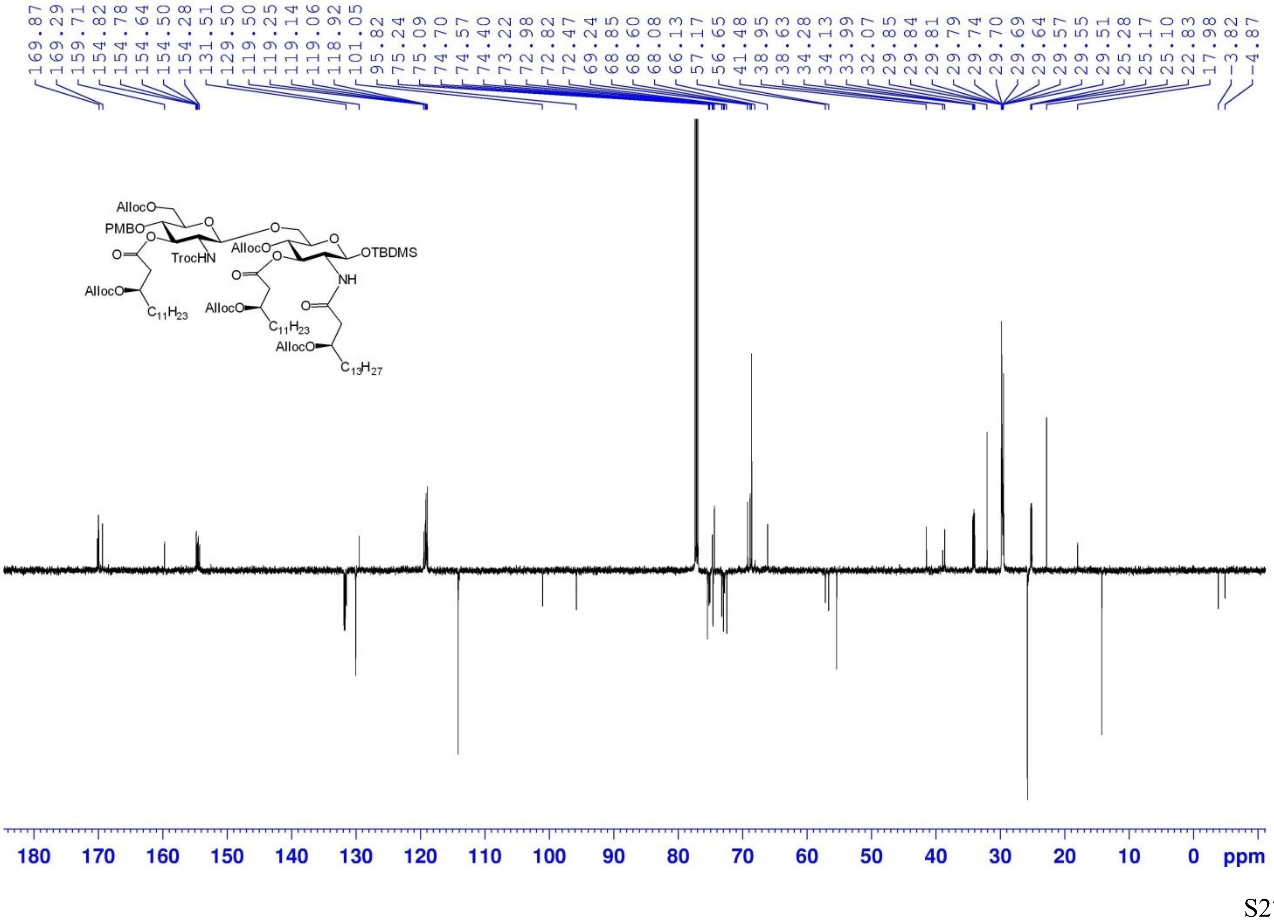

22, ^{1}H NMR, 600 MHz, $CDCl_3$

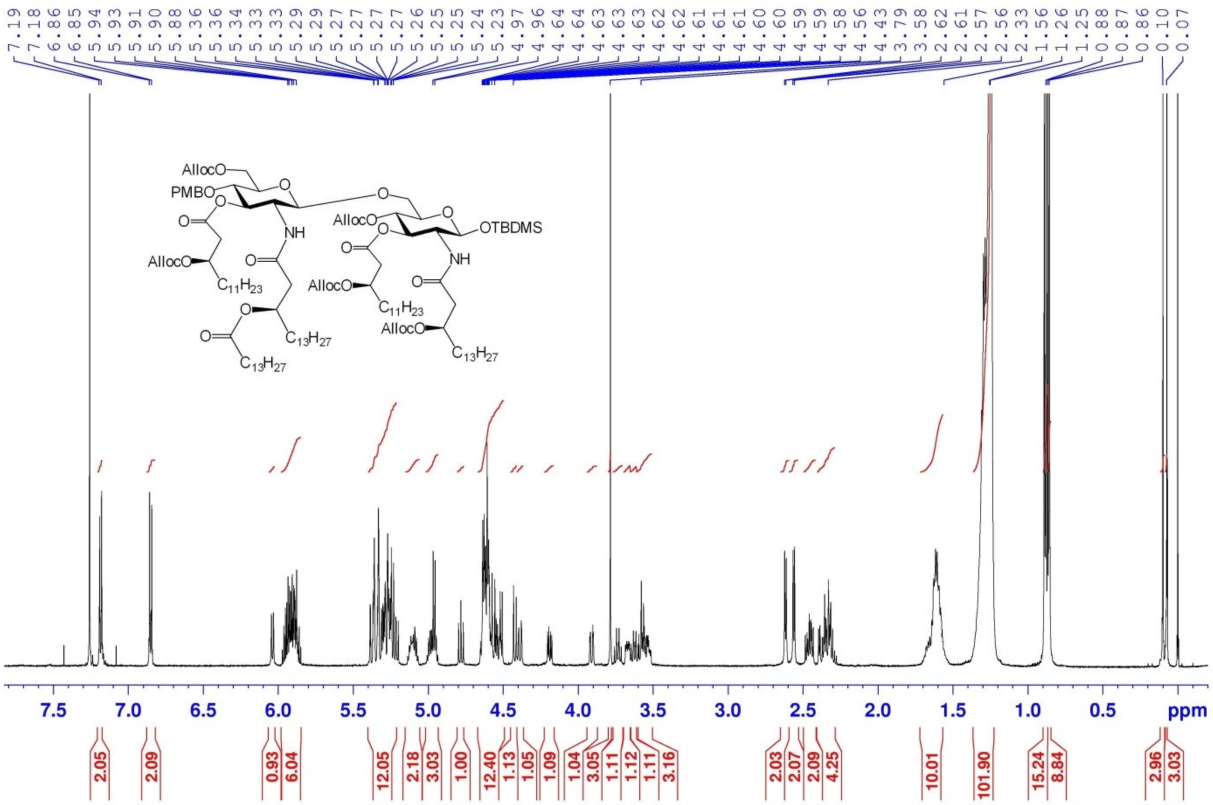

22, ^{13}C NMR, 150 MHz, $CDCl_3$

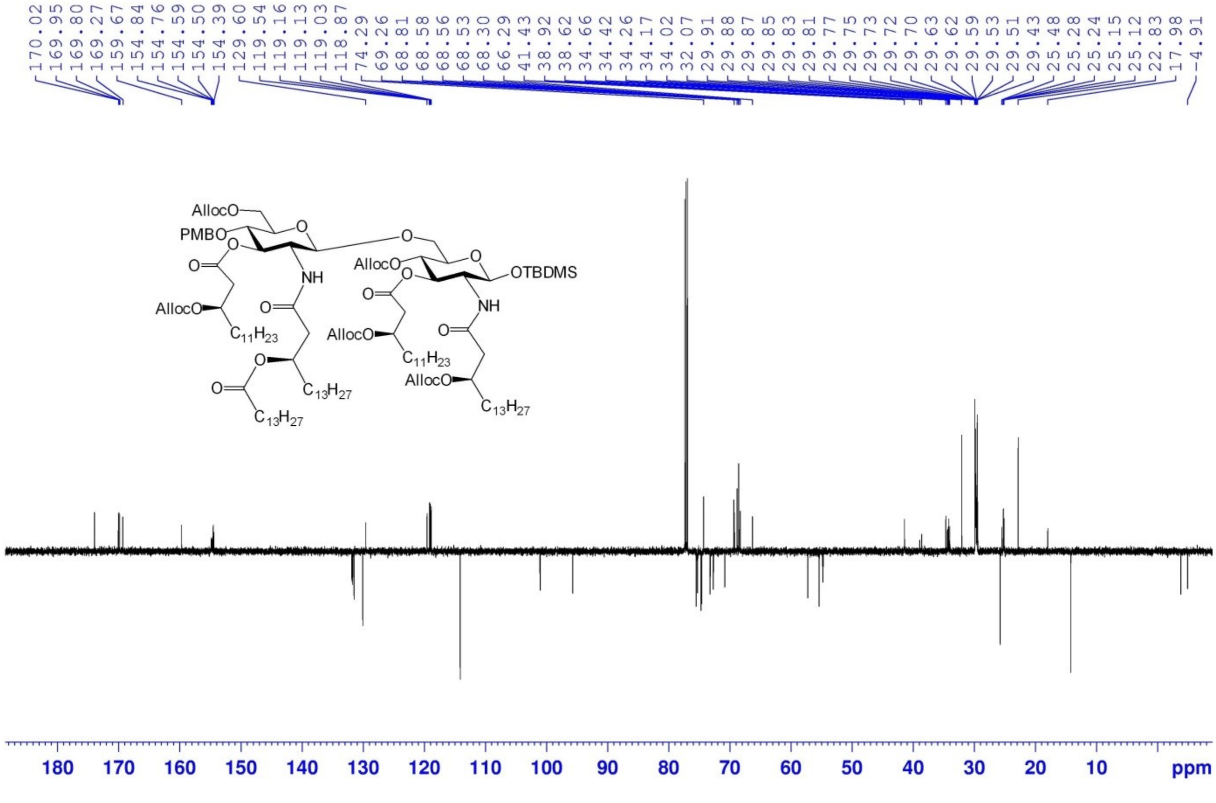

23, ^{1}H NMR, 600 MHz, $CDCl_3$

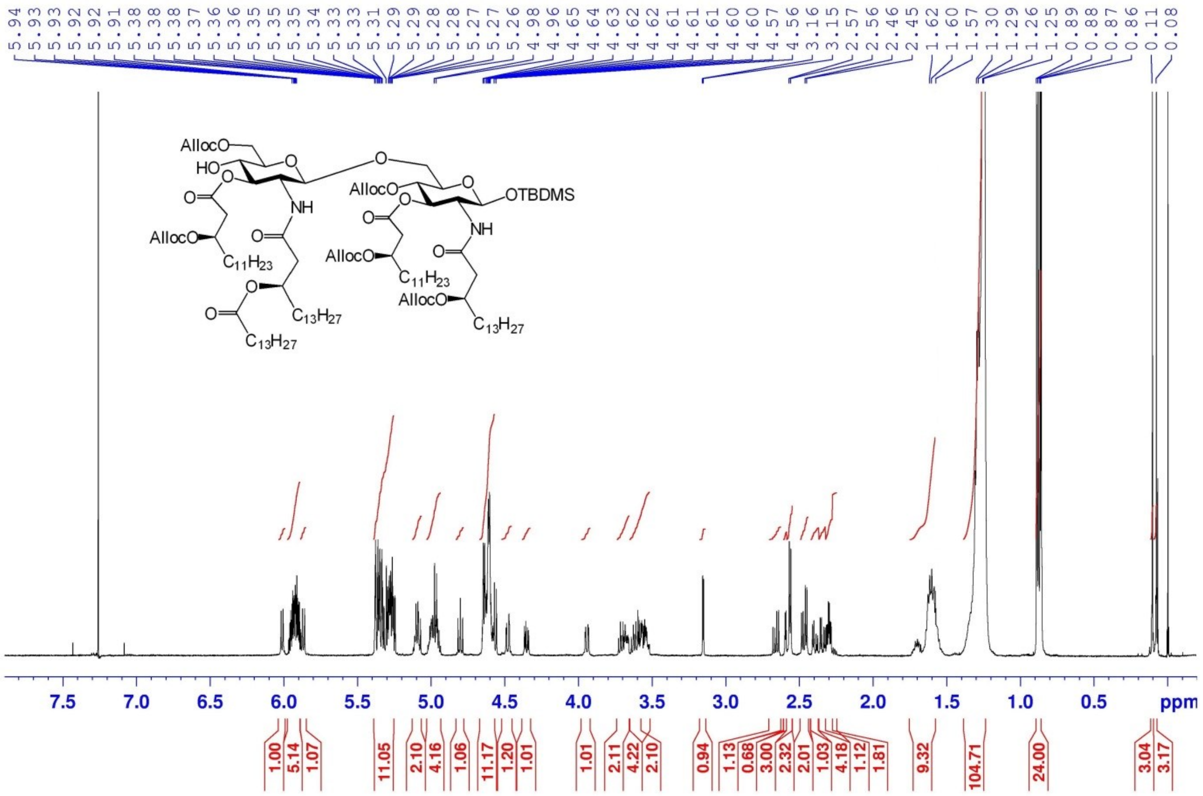

23, ^{13}C NMR, 150 MHz, $CDCl_3$

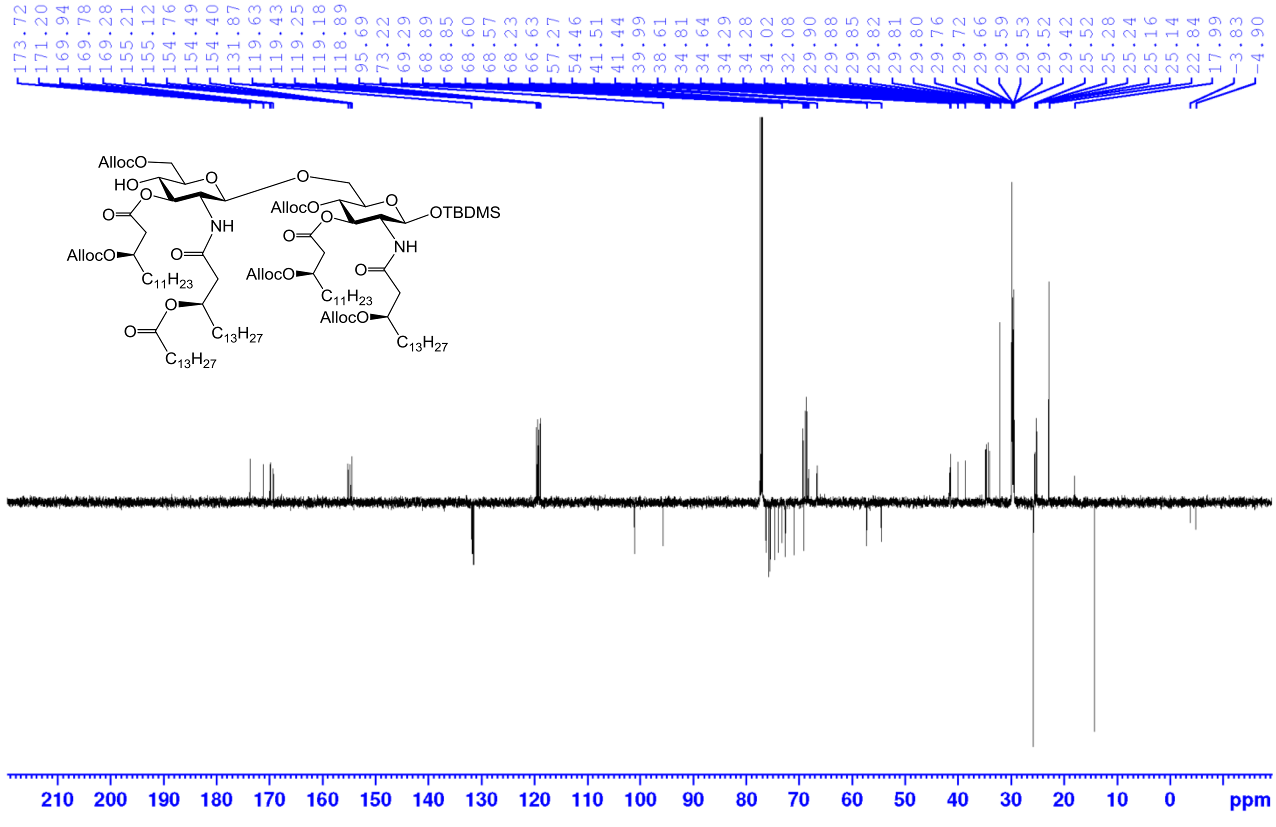

24, ^{1}H NMR, 600 MHz, $CDCl_3$

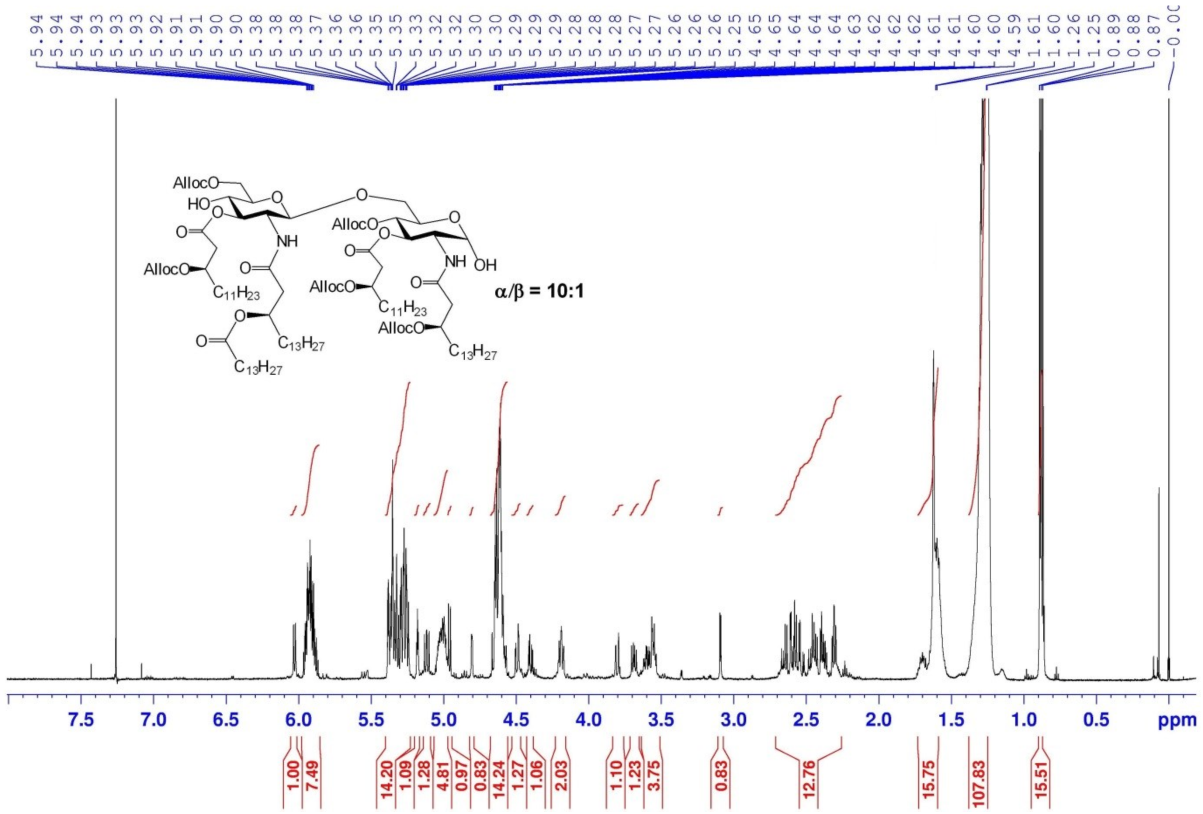

24, ^{13}C NMR, 150 MHz, $CDCl_3$

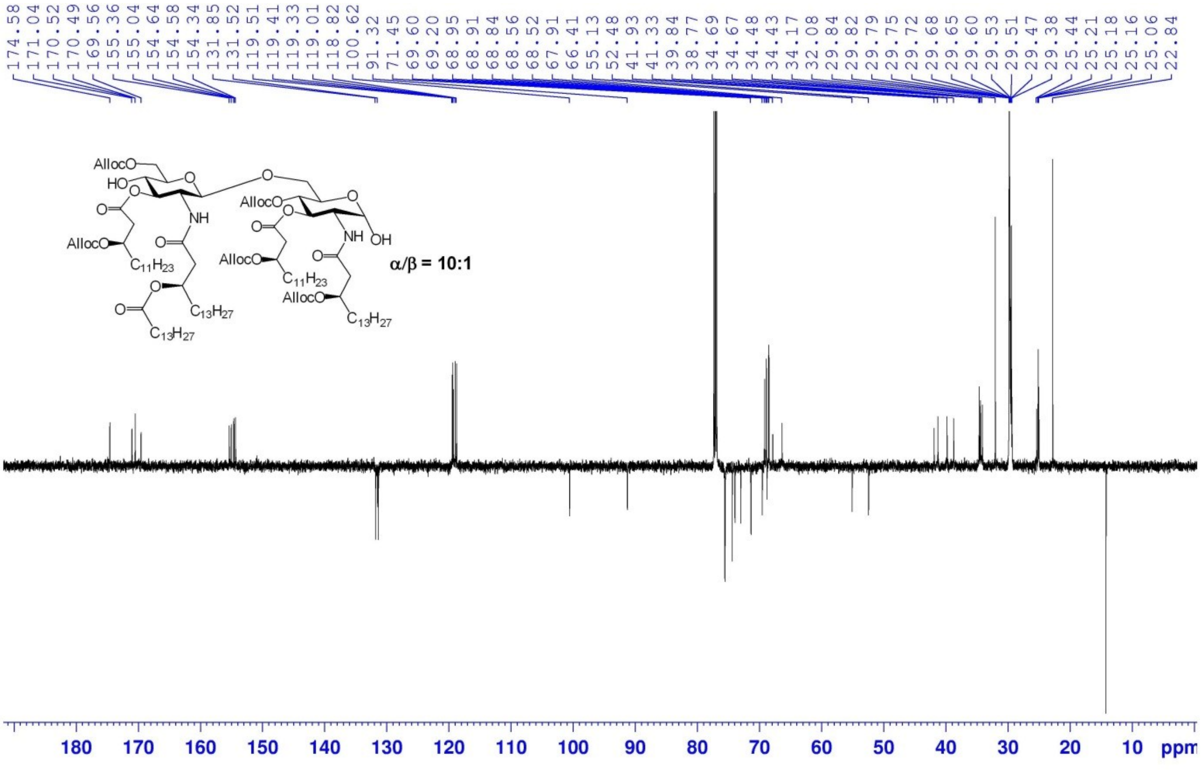

25, ^{1}H NMR, 600 MHz, $CDCl_3$

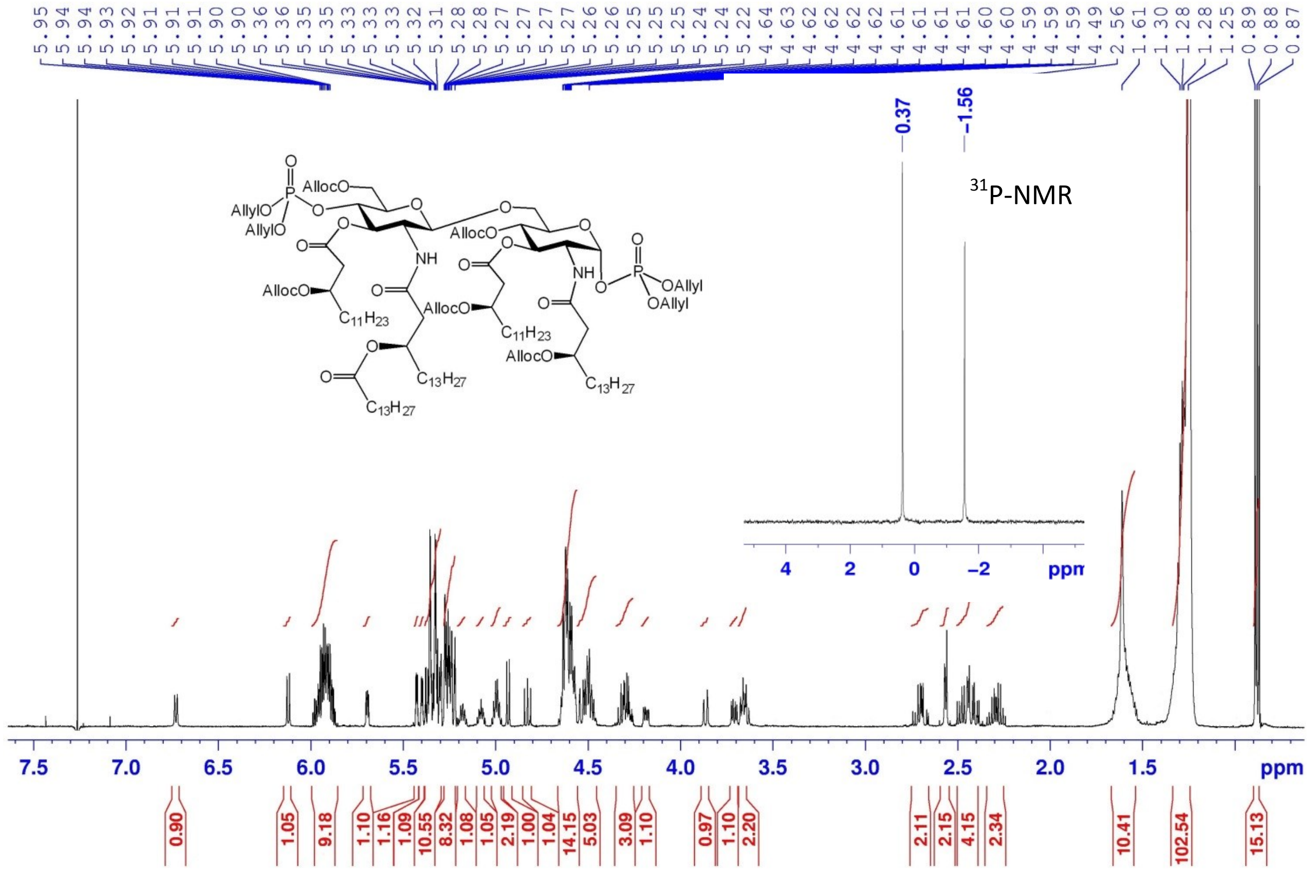

25, ^{1}H NMR, 600 MHz, $CDCl_3$, 4.5 – 6.5 ppm

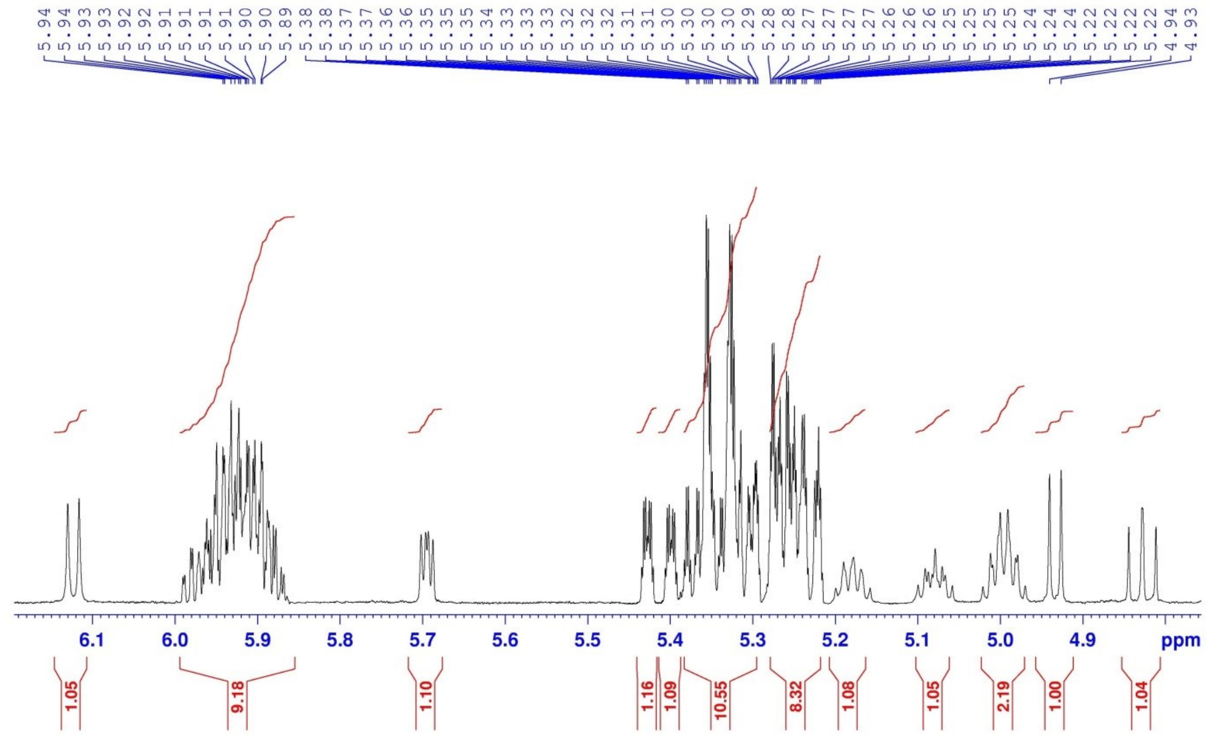

25, ^{13}C NMR, 150 MHz, $CDCl_3$

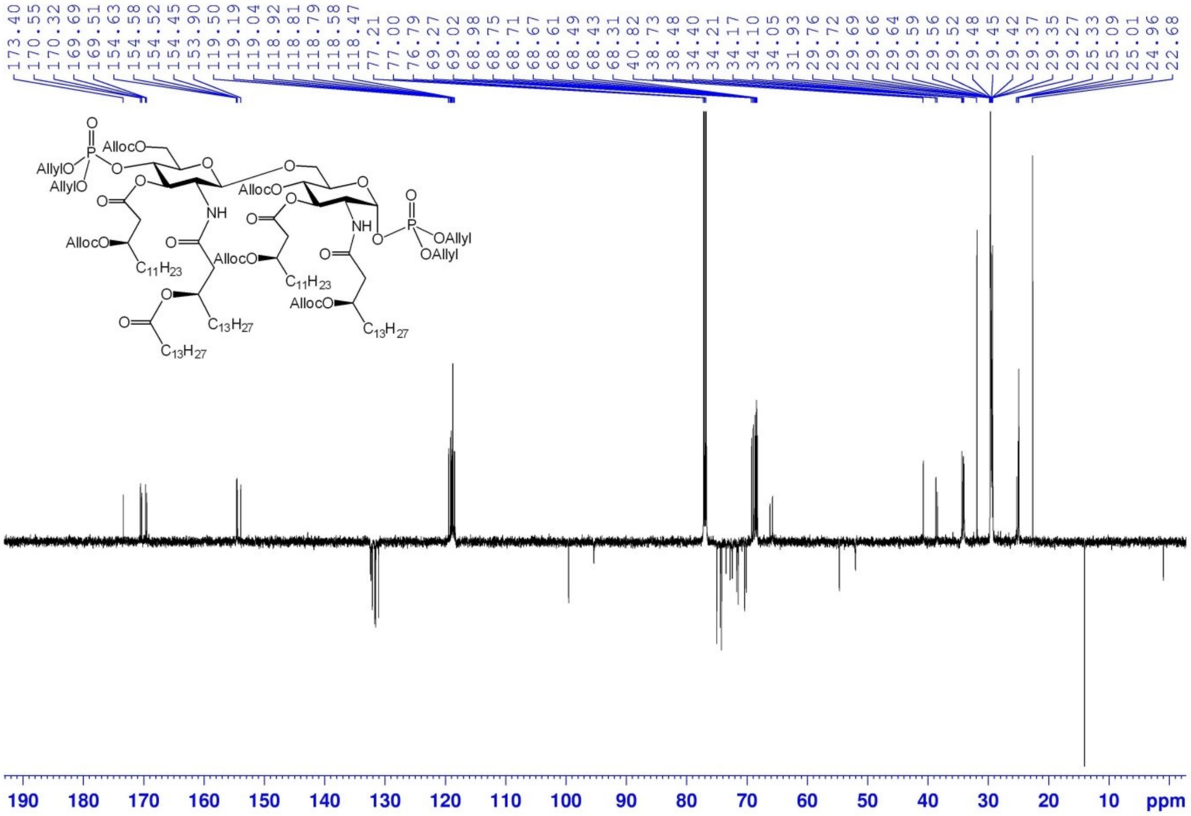

25, ^{13}C NMR, 150 MHz, $CDCl_3$, 65 – 75 ppm

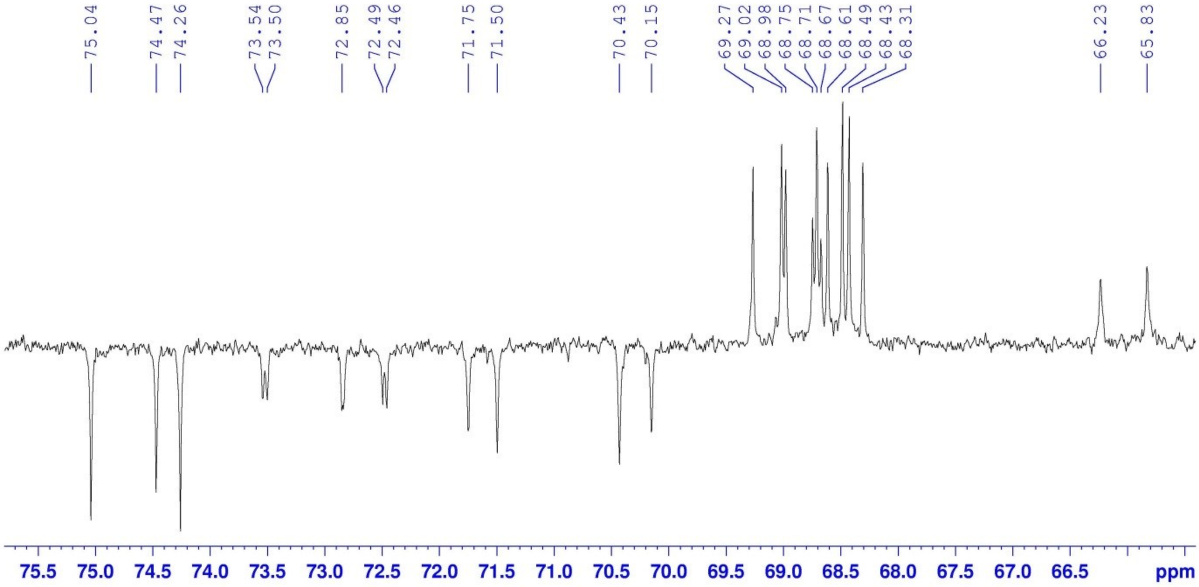

2, ^{1}H NMR, 600 MHz, $CDCl_3$ – CD_3OD, 4:1

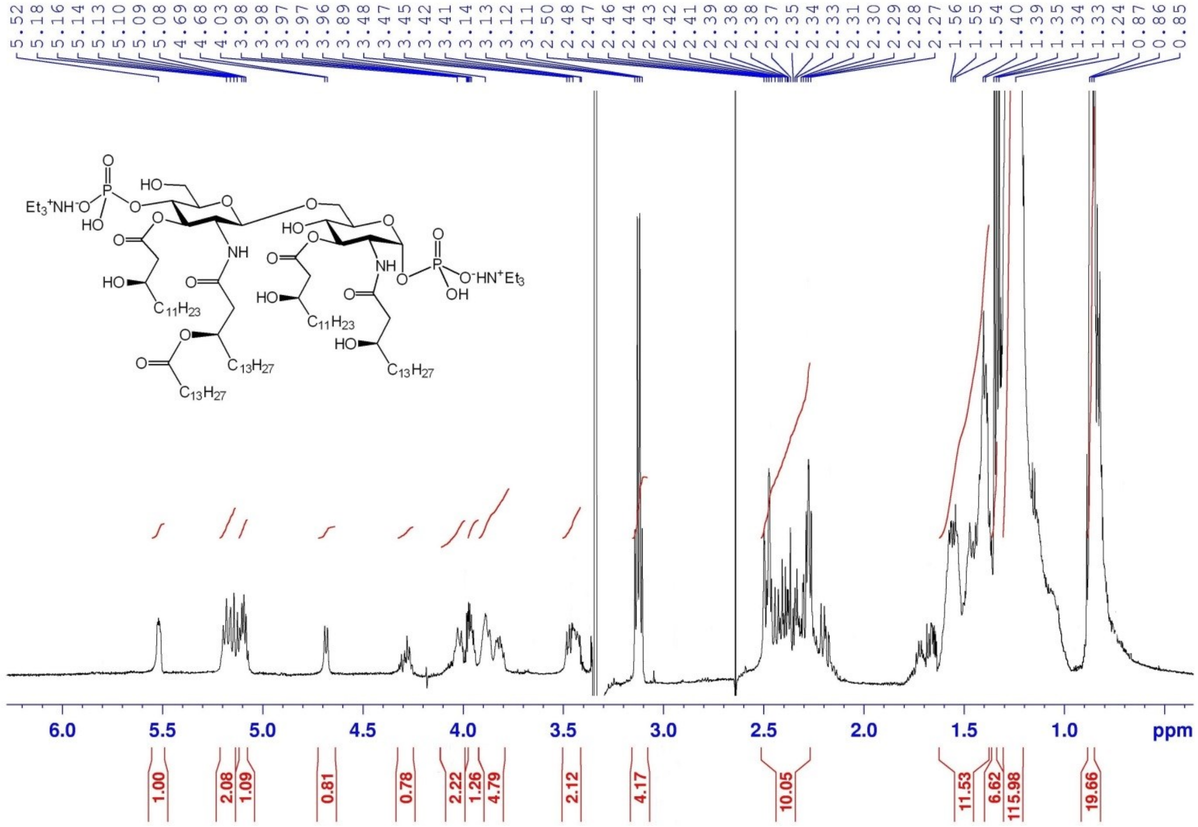

2, HSQC, $CDCl_3$ – CD_3OD, 4:1

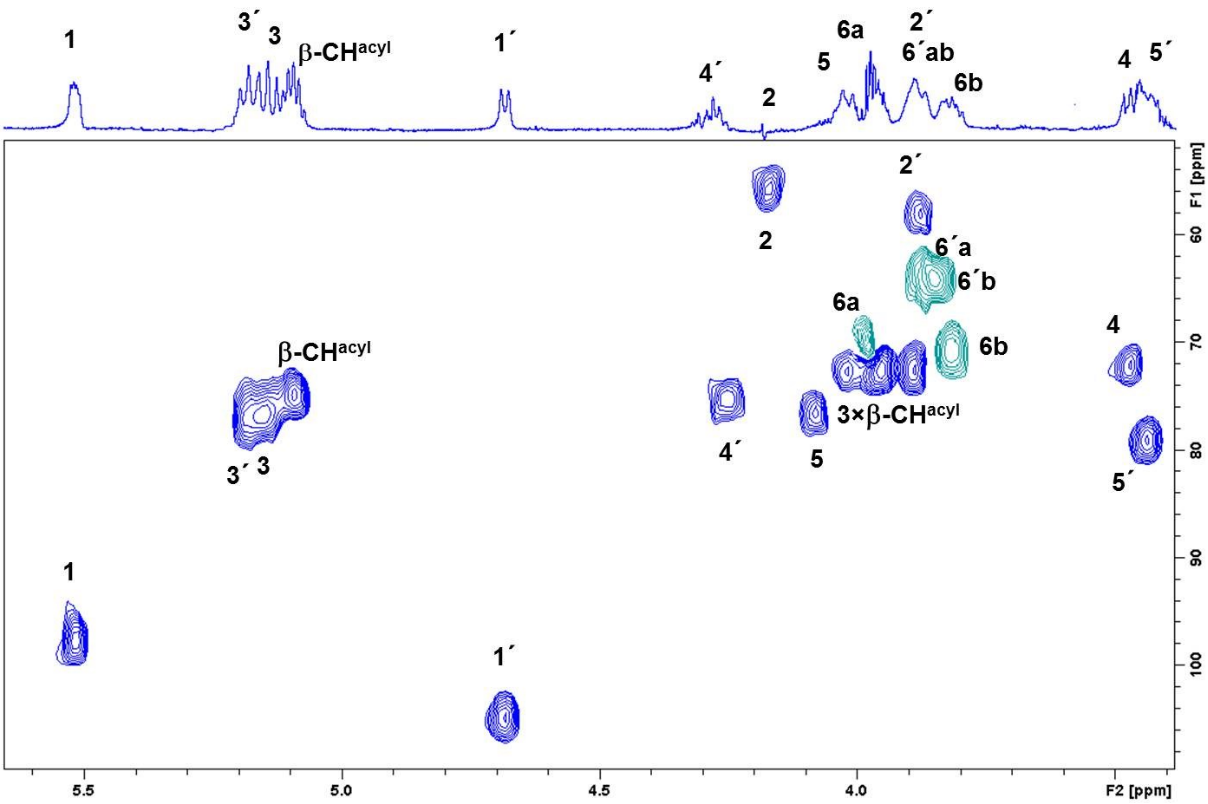

2, ESI-MS, positive mode

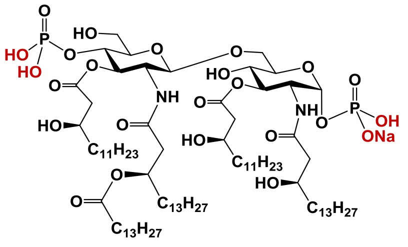

m/z: 1694.12

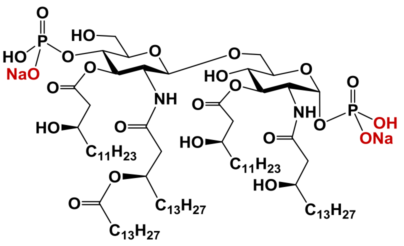

m/z: 1716.06

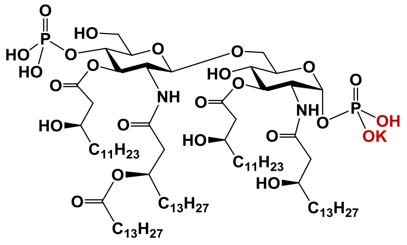

m/z: 1710.07

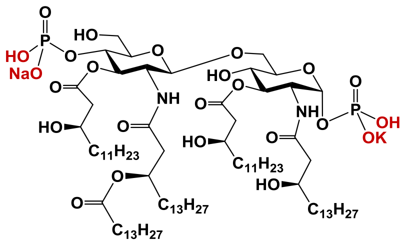

m/z: 1732.06

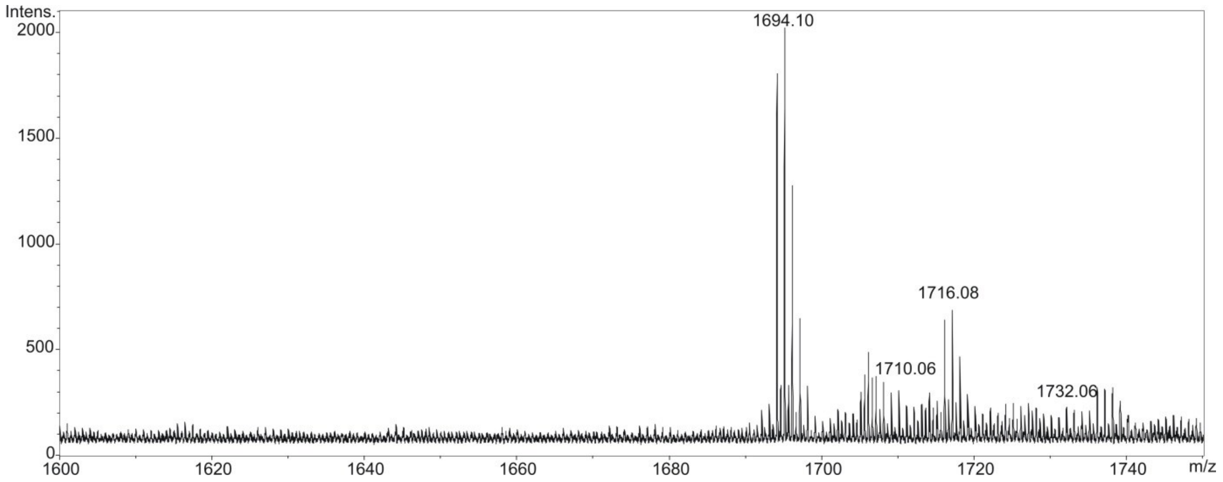

26, ^{1}H NMR, 600 MHz, $CDCl_3$

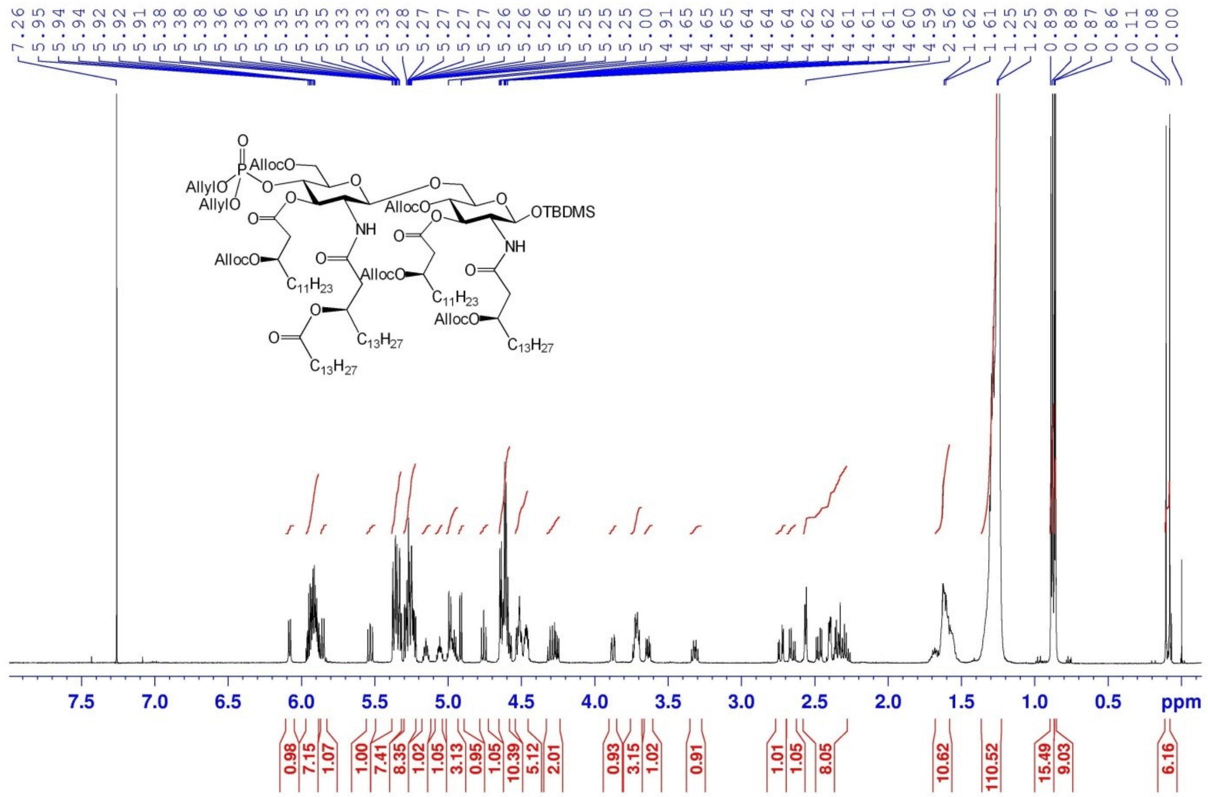

26, ^{1}H NMR, 600 MHz, $CDCl_3$, 3.5 – 6.5 ppm

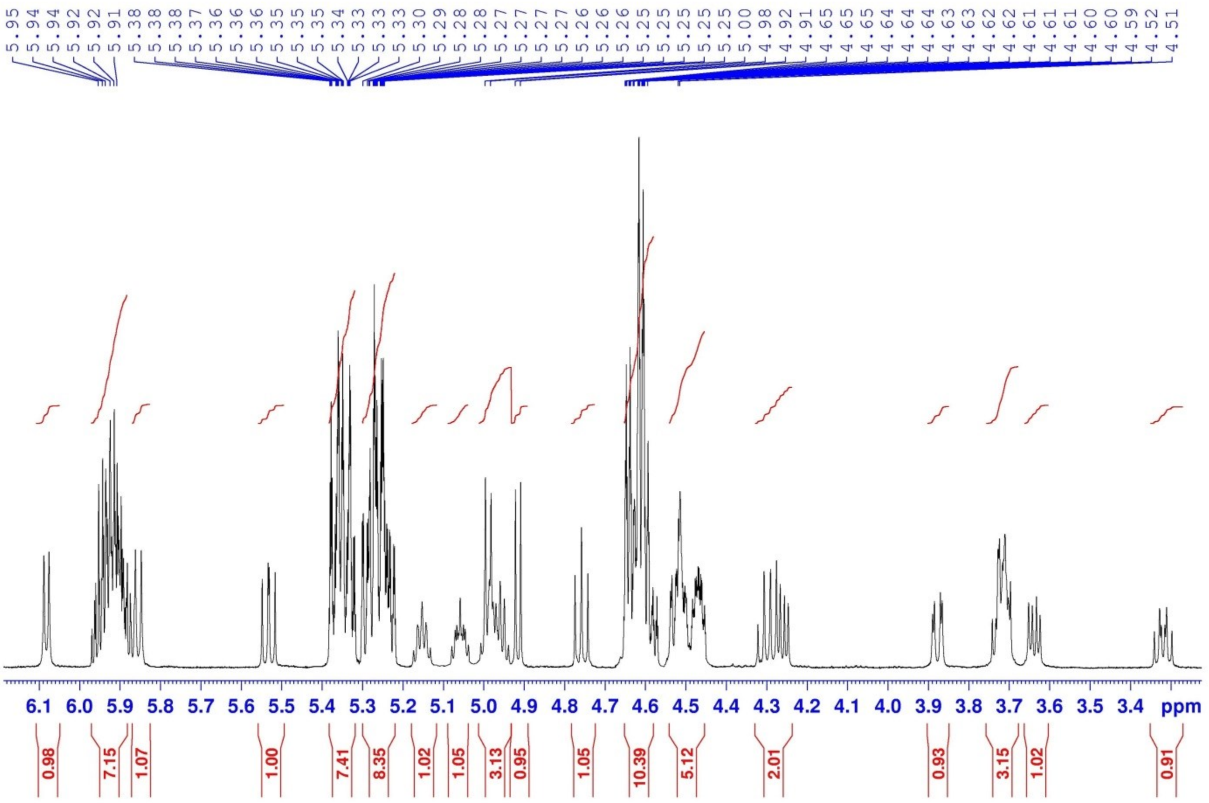

26, ^{13}C NMR, 150 MHz, $CDCl_3$

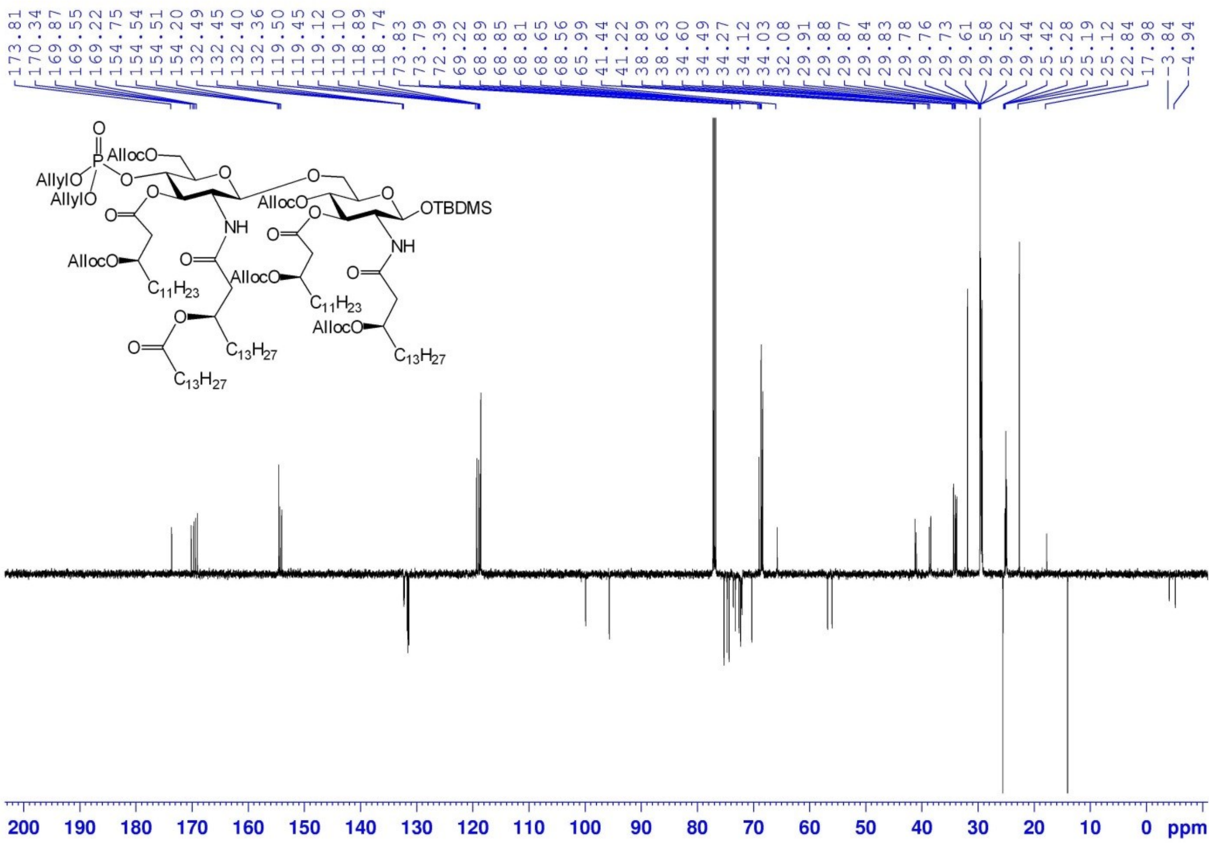

26, ^{13}C NMR, 150 MHz, $CDCl_3$, 55 -76 ppm

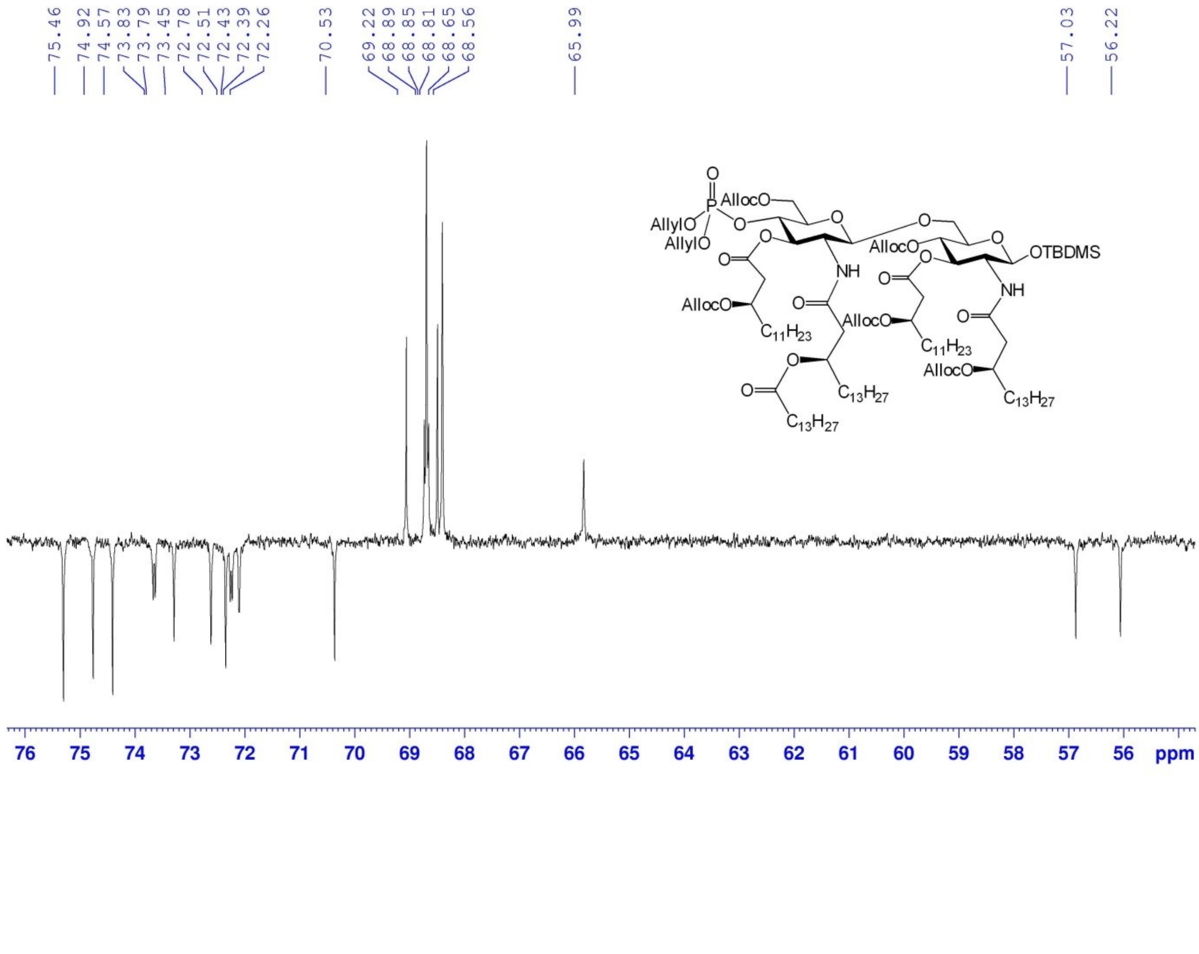

27, ^{1}H NMR, 600 MHz, $CDCl_3$

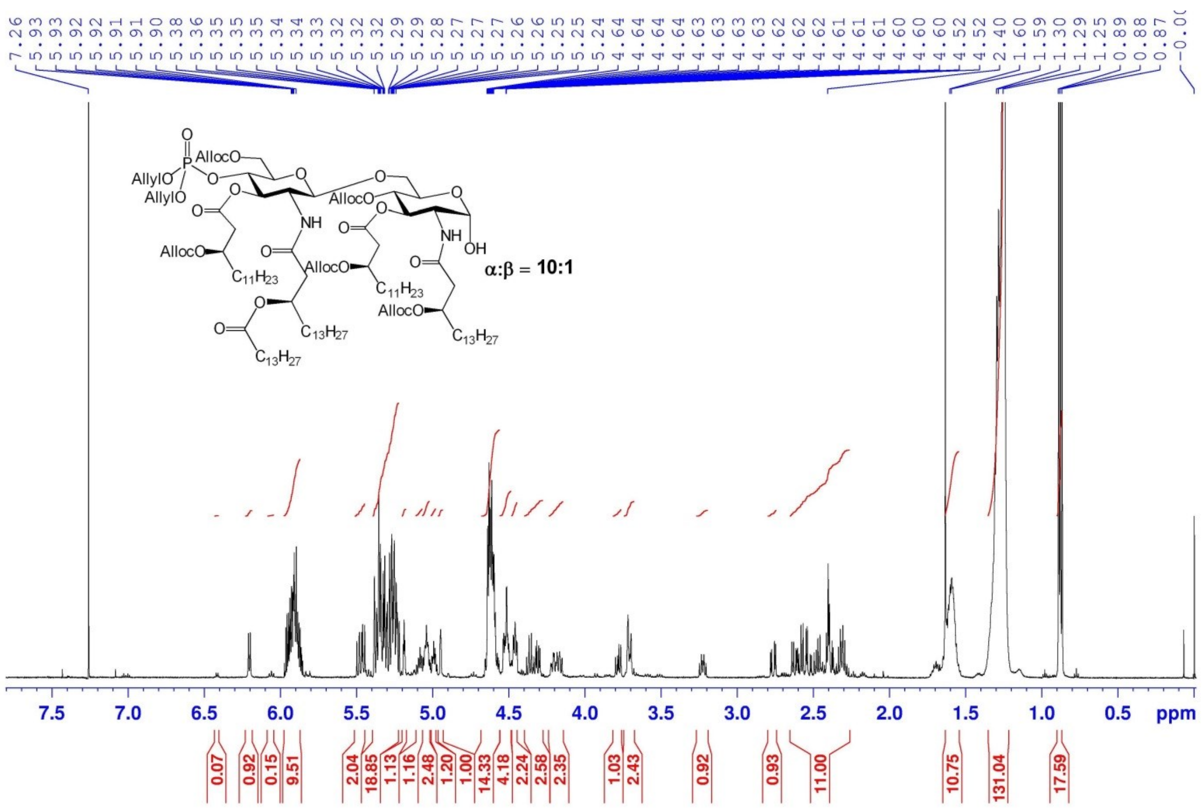

27, ^{13}C NMR, 150 MHz, $CDCl_3$

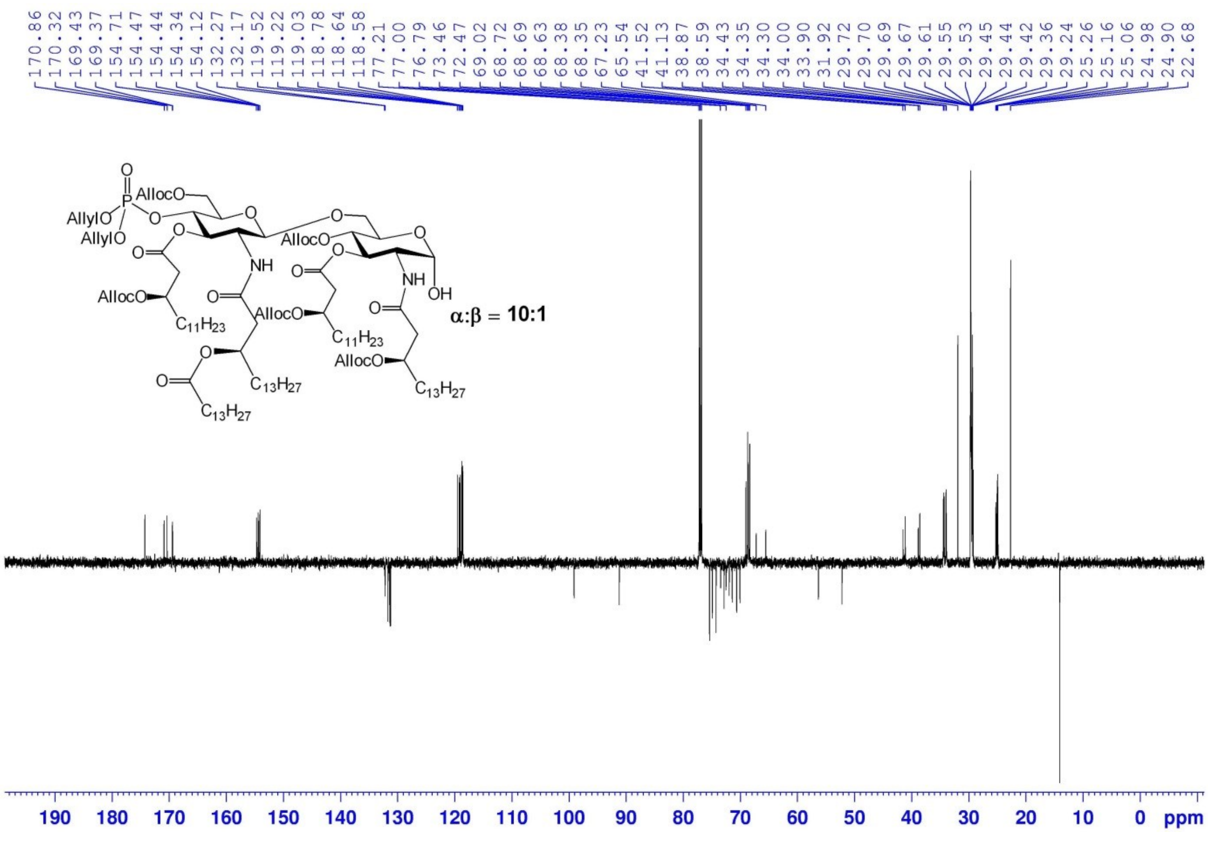

28, ^{1}H NMR, 600 MHz, $CDCl_3$ – MeOD, 4:1

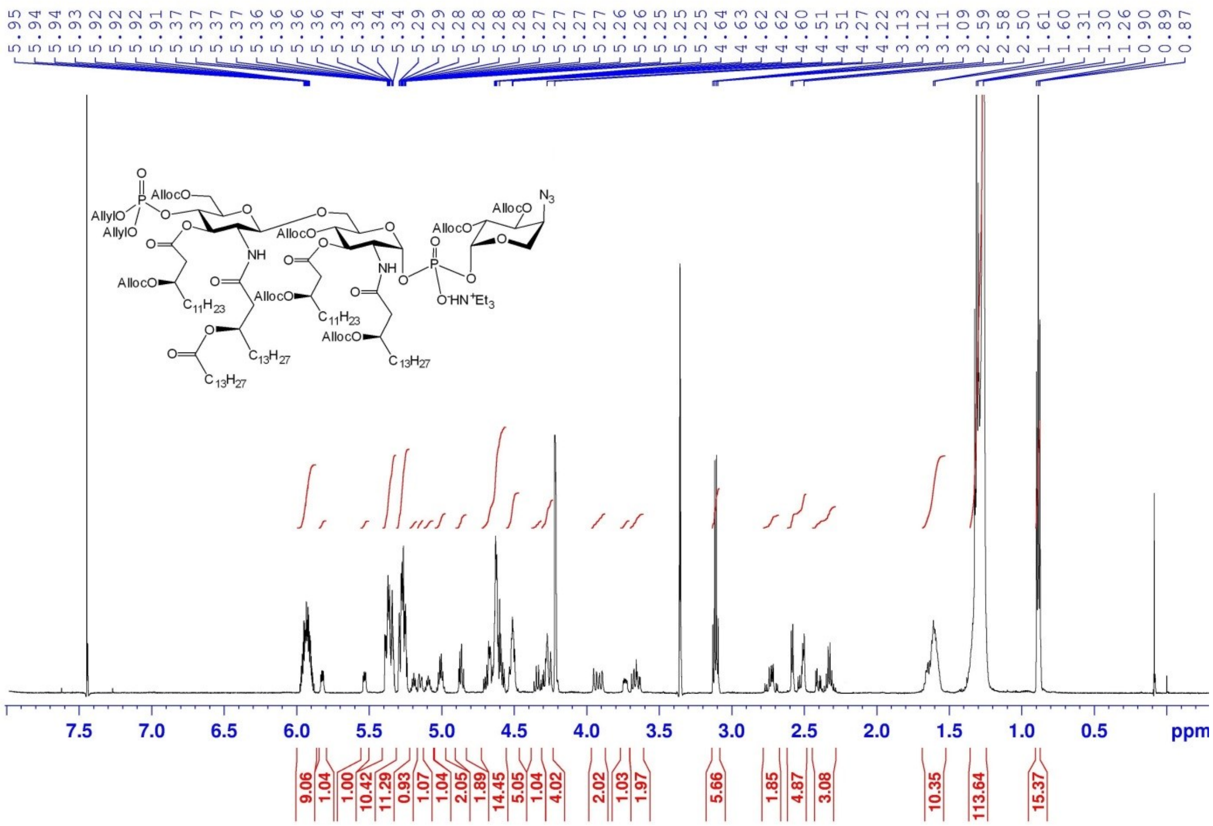

28, ^{1}H NMR, 600 MHz, $CDCl_3$ – MeOD, 4:1, 4.5 – 6.0 ppm

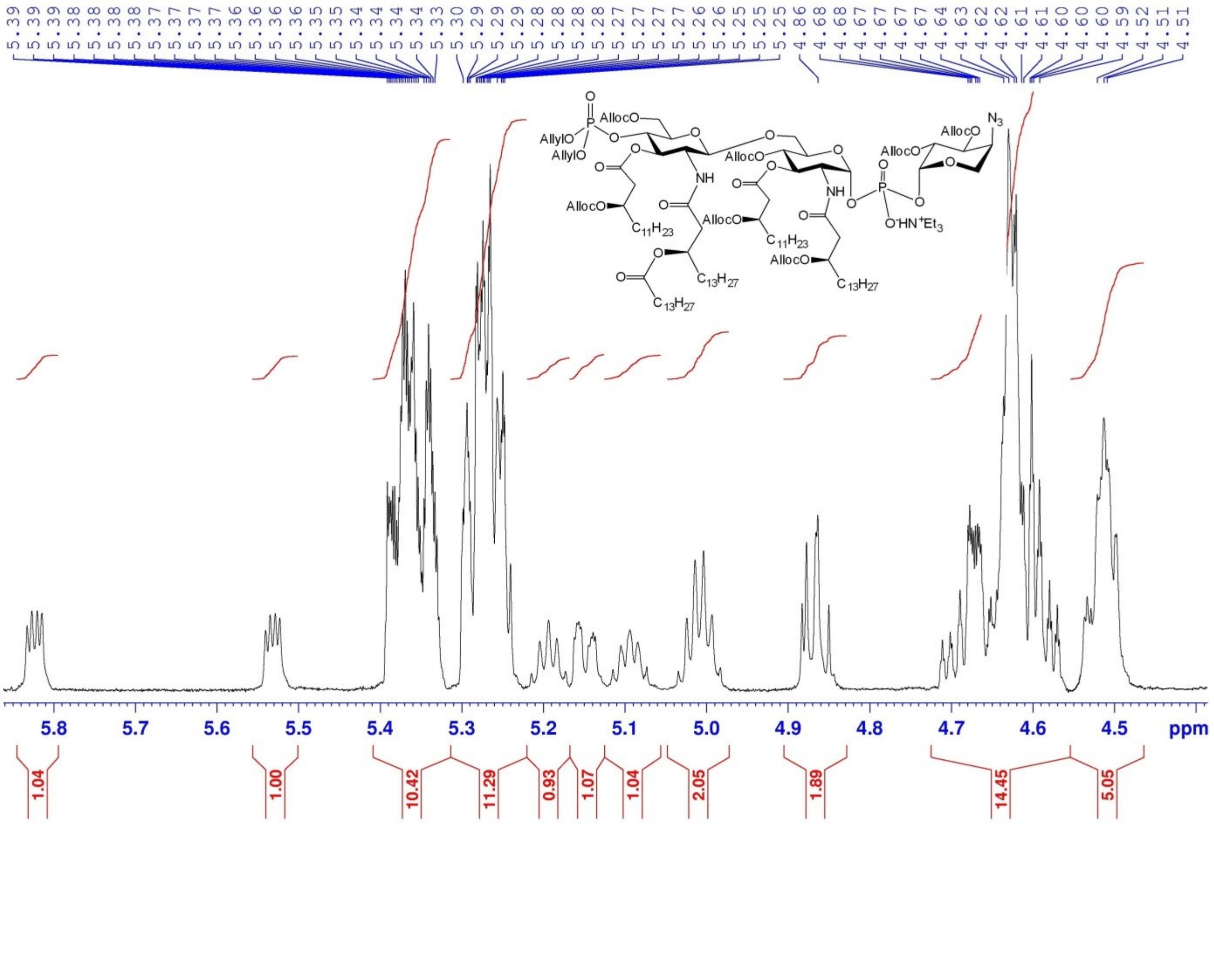

28, ^{13}C NMR, 150 MHz, $CDCl_3$ – MeOD, 4:1

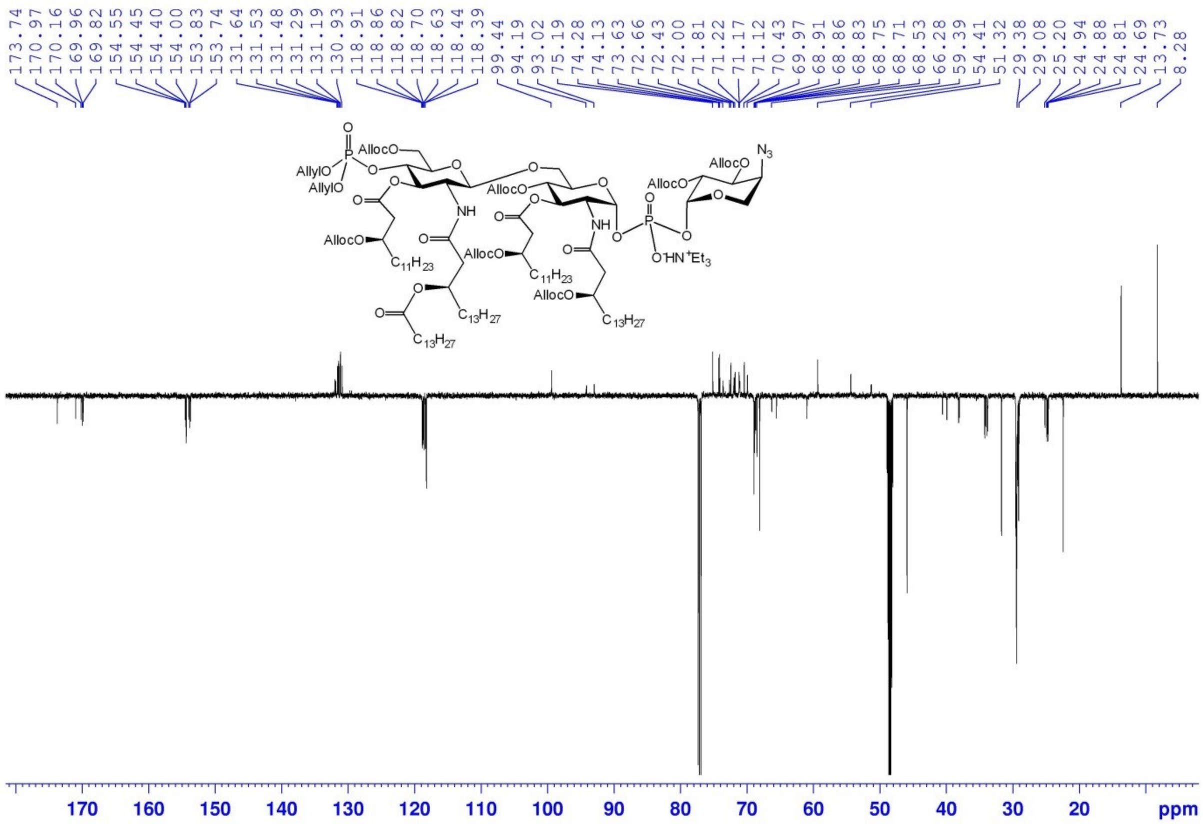

28, ^{13}C NMR, 150 MHz, $CDCl_3$ – MeOD, 4:1

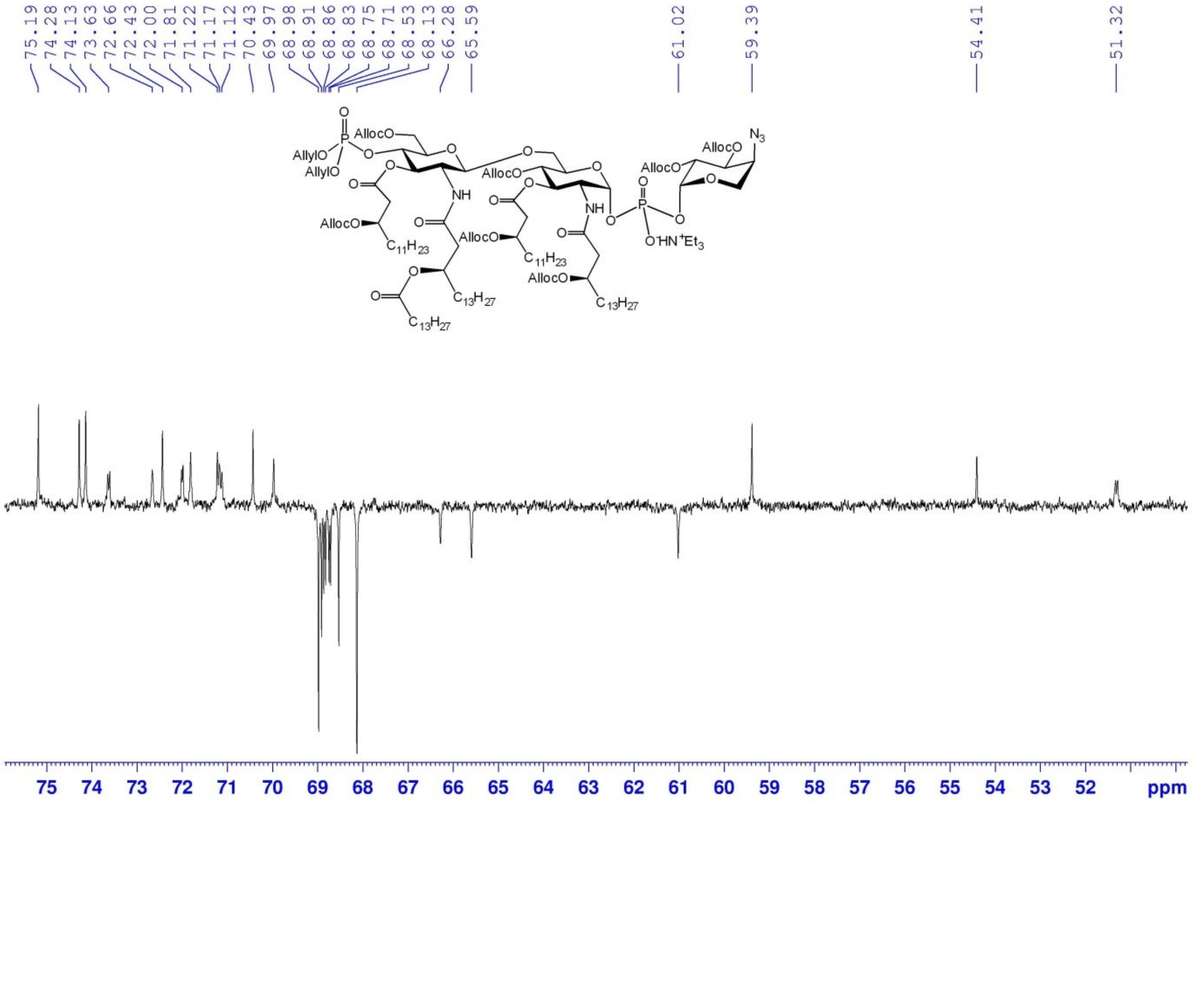

28, COSY, $CDCl_3$ – MeOD, 4:1, 3.5 – 6.0 ppm

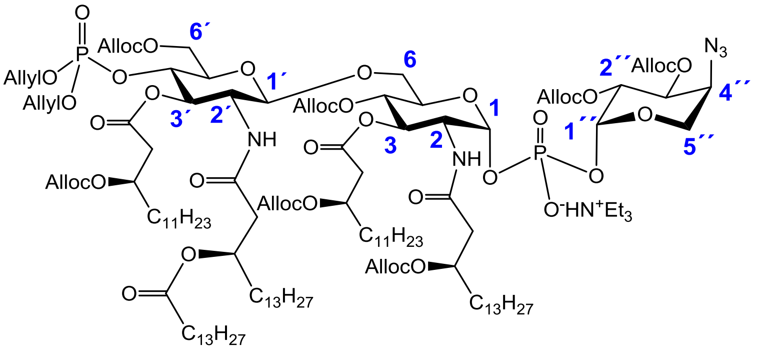

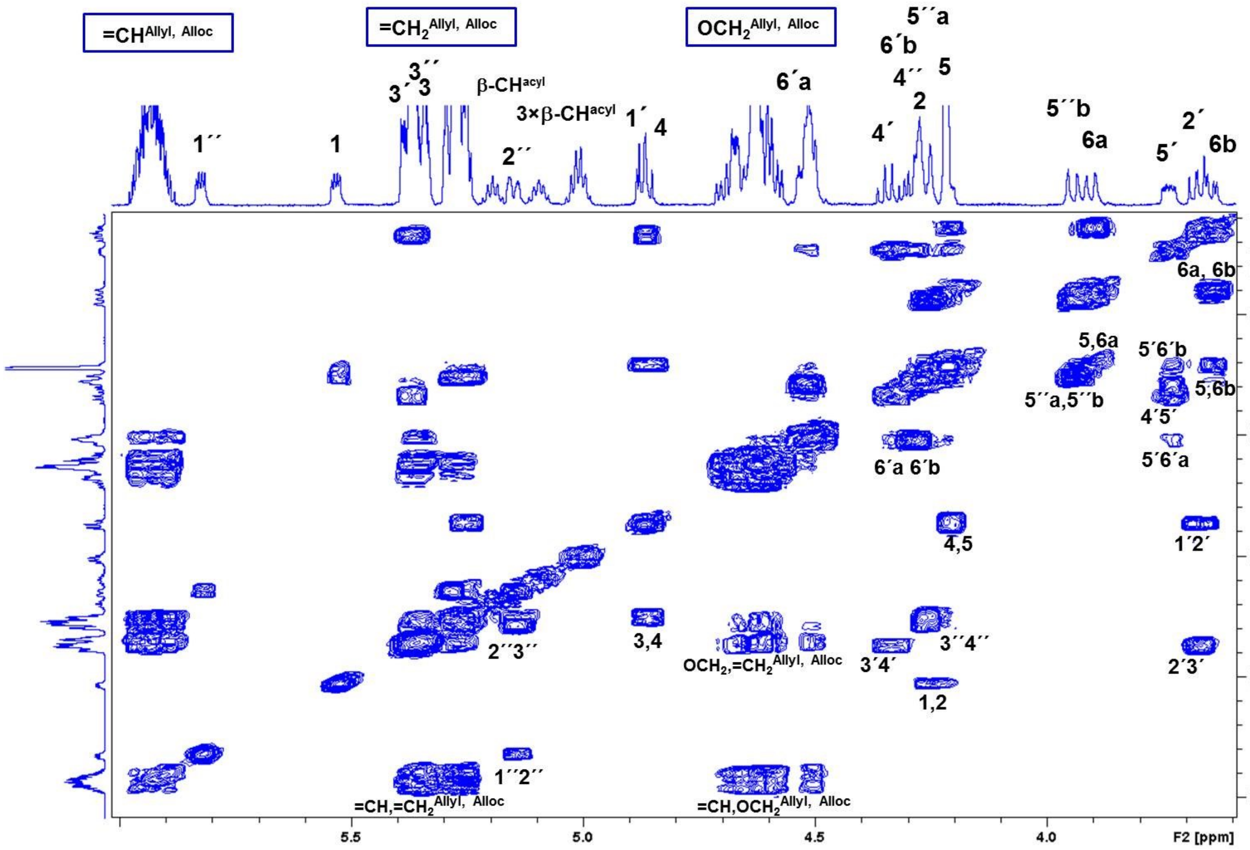

28, HSQC, $CDCl_3$ – MeOD, 4:1

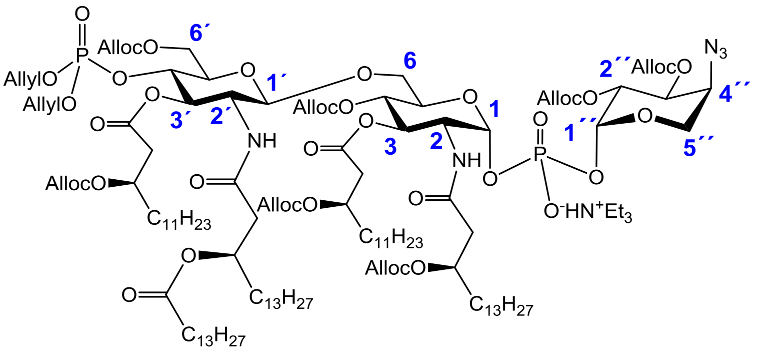

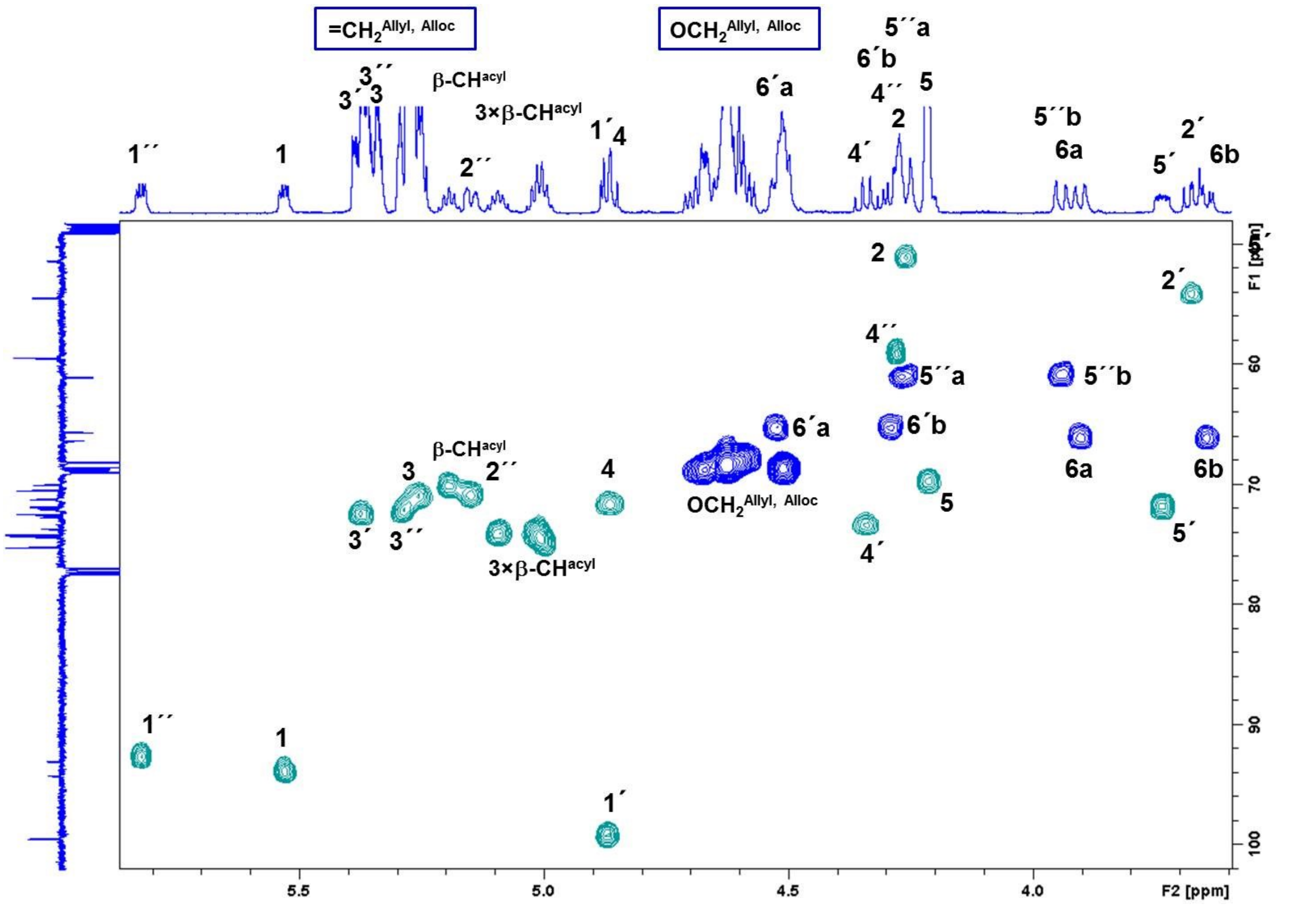

28, COSY, $CDCl_3$ – MeOD, 4:1, 2.0 – 6.0 ppm

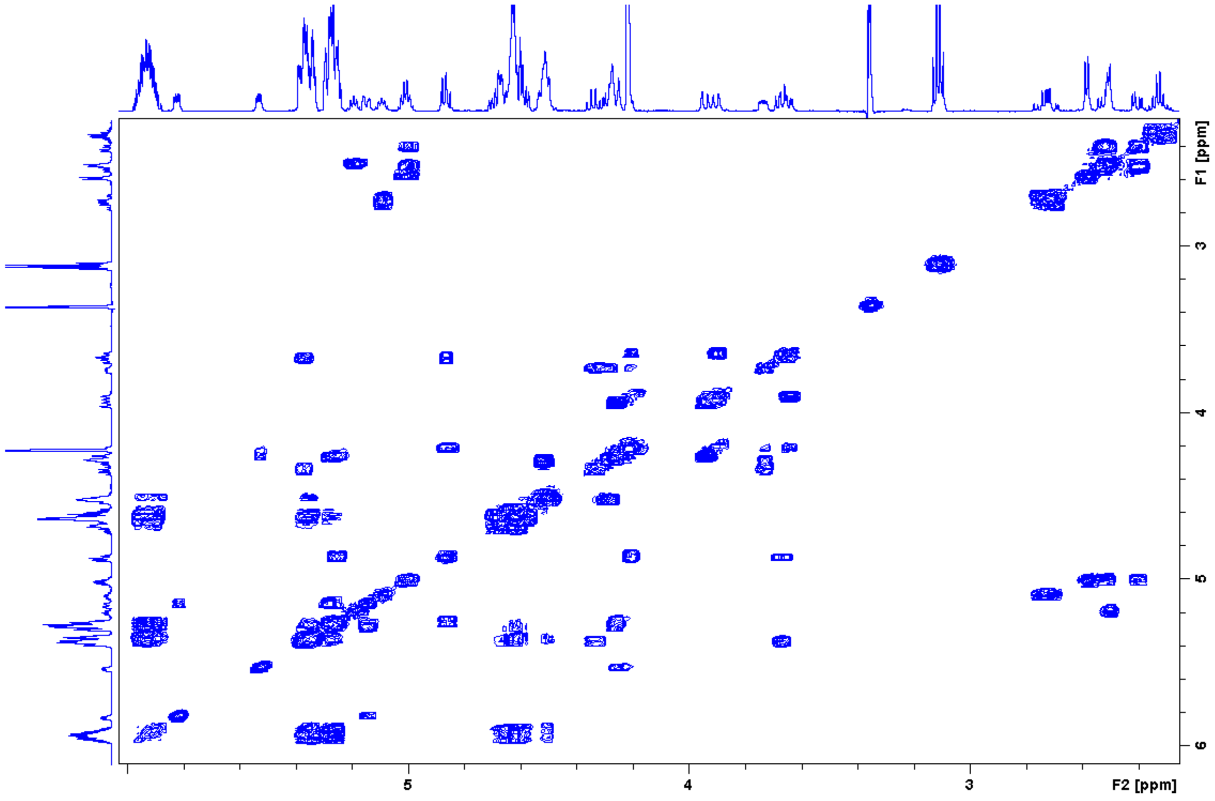

28, HSQC full, $CDCl_3$ – MeOD, 4:1

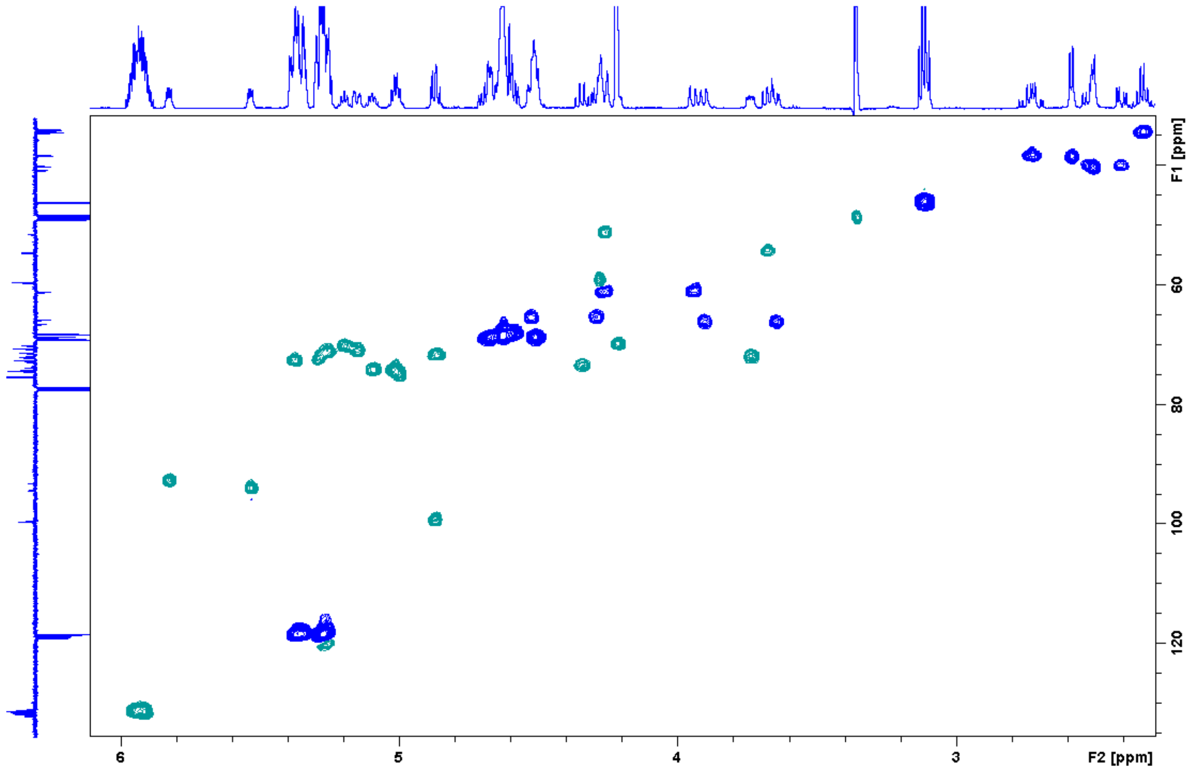

1, ^{1}H NMR, 600 MHz, $CDCl_3$ – MeOD, 4:1

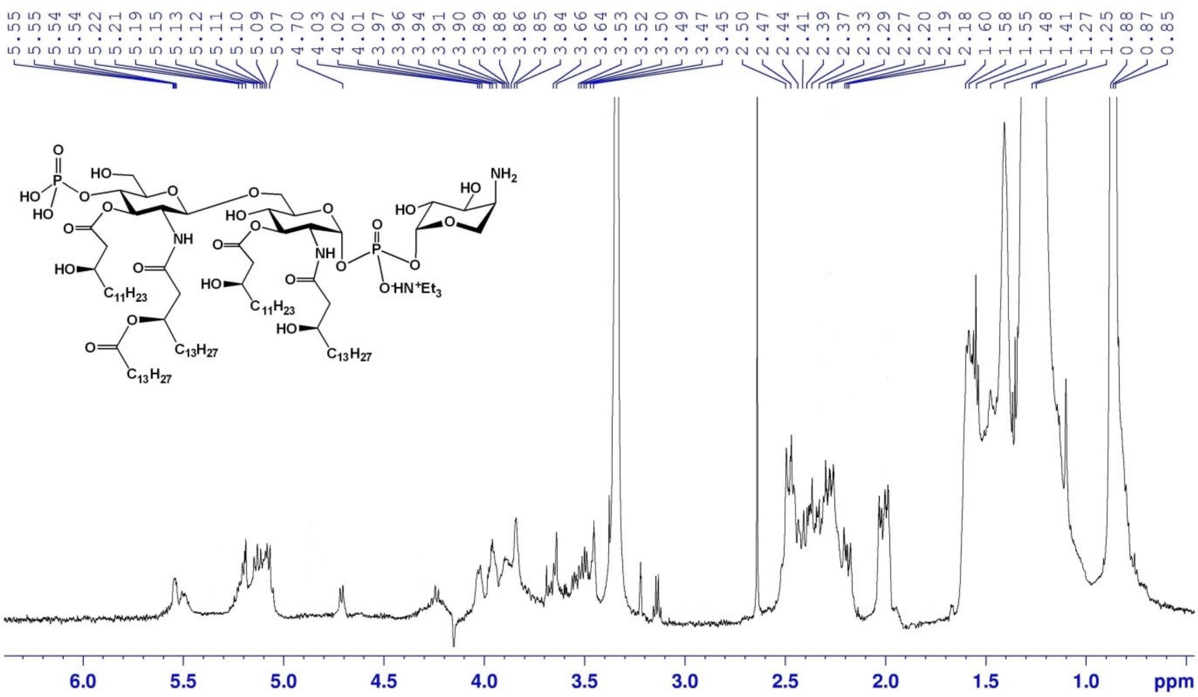

1, HSQC, $CDCl_3$ – MeOD, 4:1

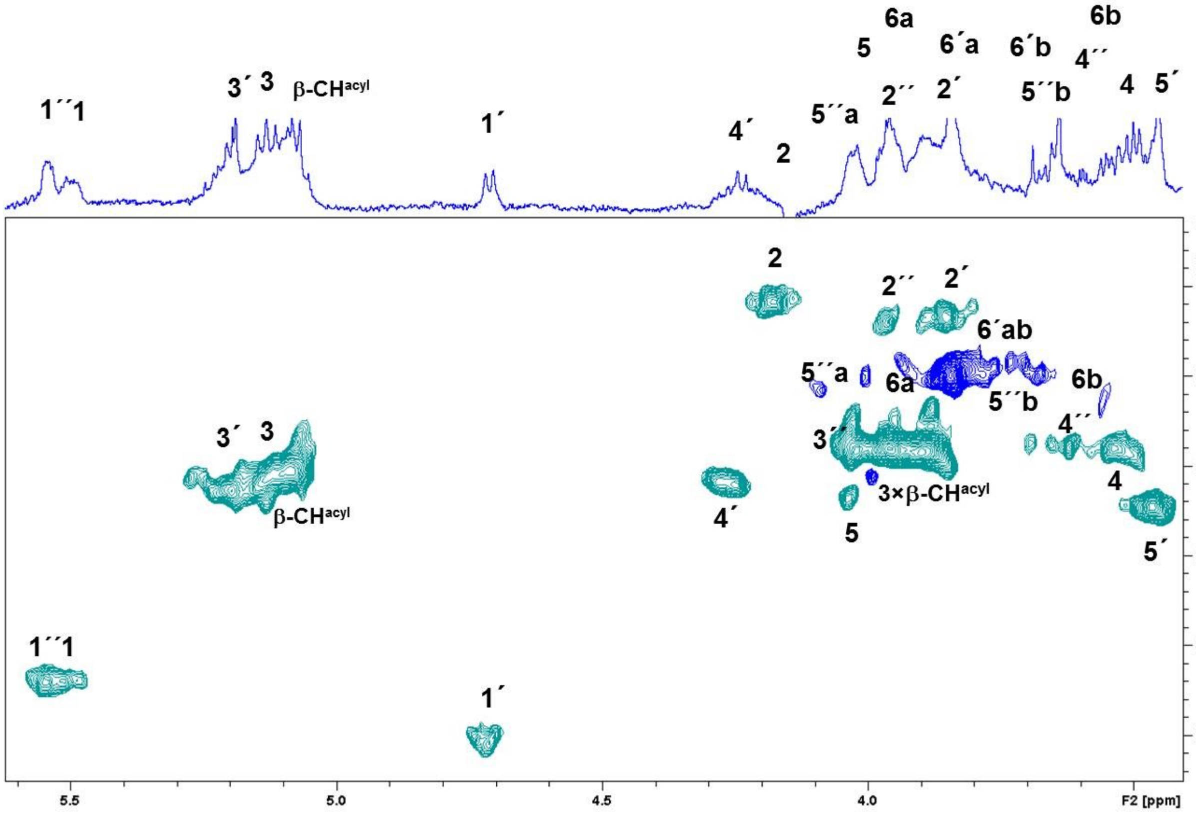

1, ESI-MS, positive mode:

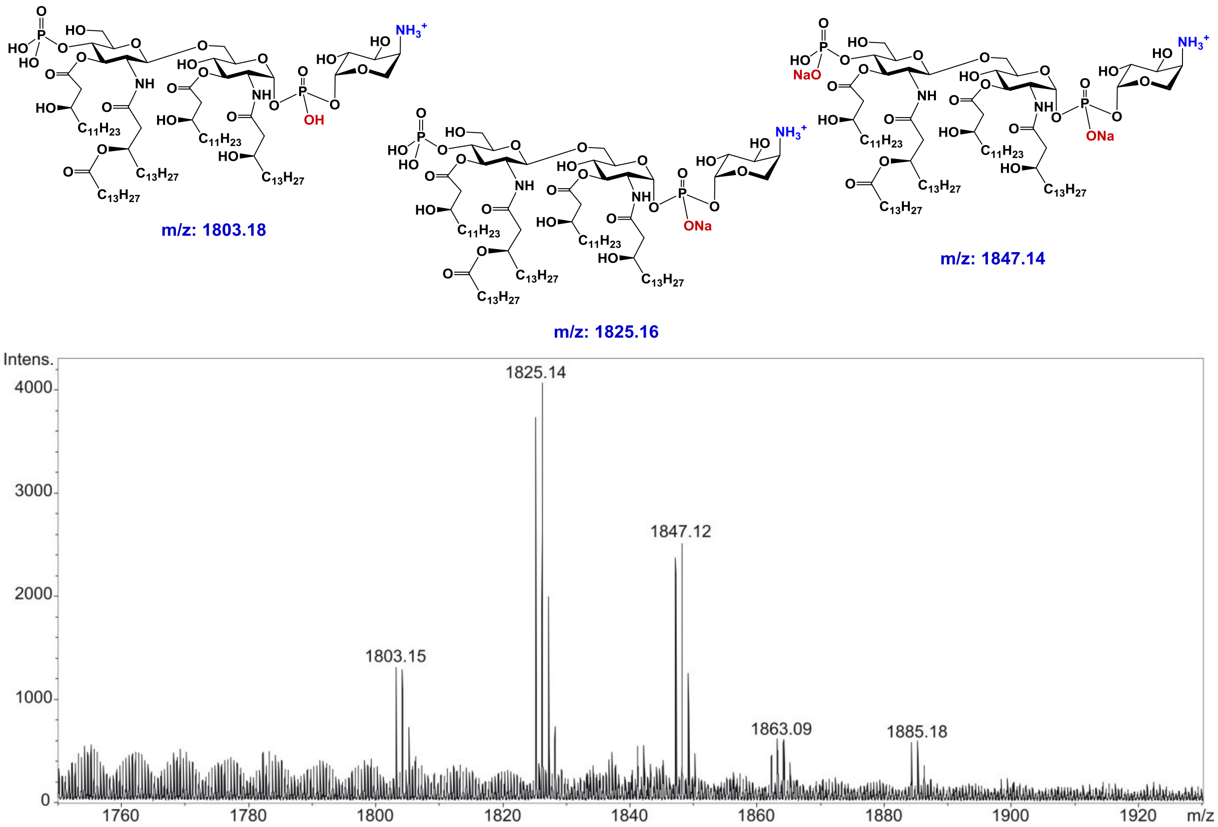

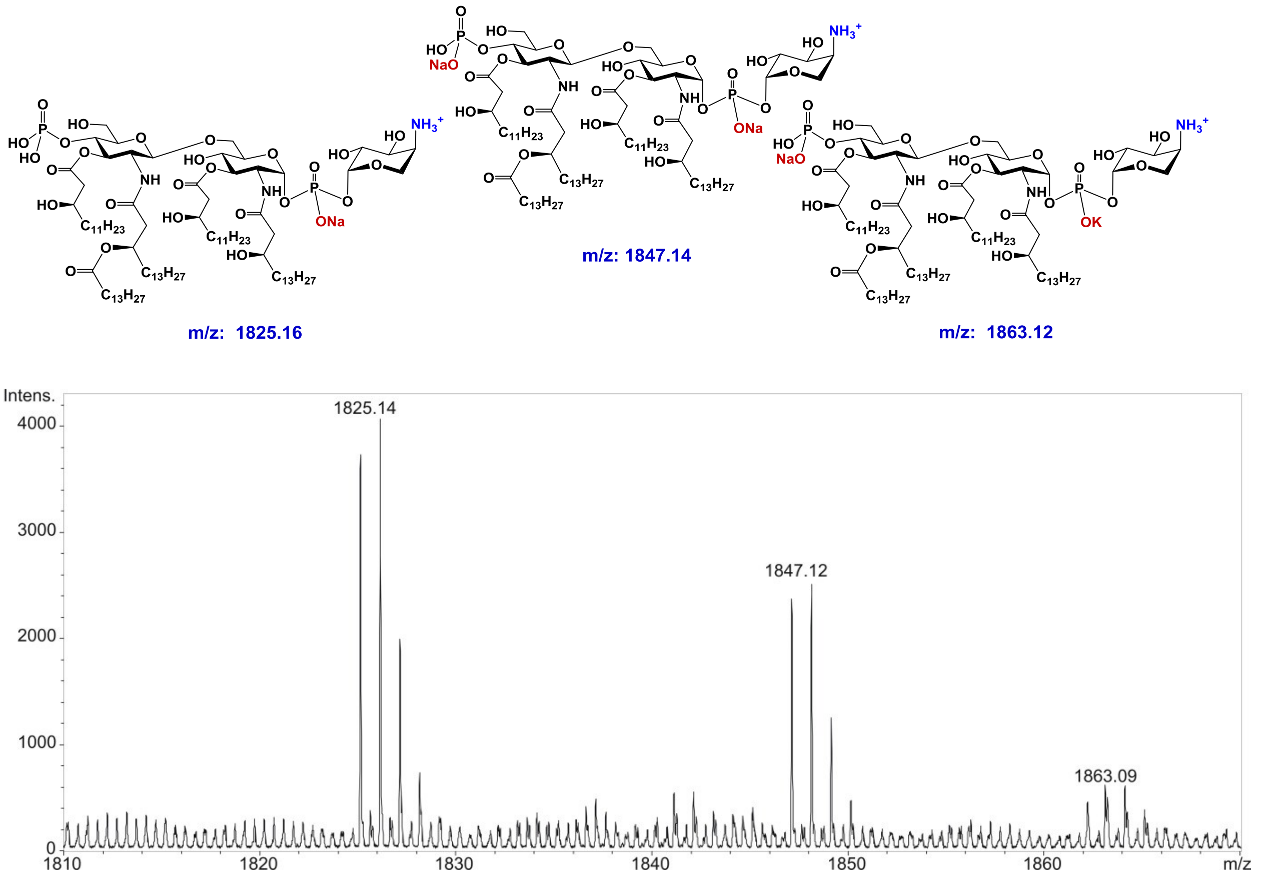

Supplement: Supplementary file 1 — miscellaneous_information [file chem0021-4102-sd1.pdf]
